# Comparative effectiveness and safety of pharmacological and non-pharmacological interventions for insomnia: an overview of reviews

**DOI:** 10.1186/s13643-019-1163-9

**Published:** 2019-11-15

**Authors:** Patricia Rios, Roberta Cardoso, Deanna Morra, Vera Nincic, Zahra Goodarzi, Bechara Farah, Sharada Harricharan, Charles M. Morin, Judith Leech, Sharon E. Straus, Andrea C. Tricco

**Affiliations:** 1 Knowledge Translation Program, Li Ka Shing Knowledge Institute, St. Michael’s Hospital, Unity Health Toronto, 209 Victoria Street, East Building, Toronto, Ontario M5B 1W8 Canada; 2 Division of Geriatrics, Cumming School of Medicine, University of Calgary, Calgary, Alberta Canada; 3 The Canadian Agency for Drugs and Technologies in Health, 865 Carling Ave., Suite 600, Ottawa, Ontario K1S 5S8 Canada; 4 École de Psychologie, 2325, rue des Bibliothèques, Québec, Québec G1V 0A6 Canada; 5 Division of Respirology, Department of Medicine, University of Ottawa, Ottawa, ON Canada; 6 Department of Geriatric Medicine, University of Toronto, Toronto, Ontario Canada; 7 Epidemiology Division, Dalla Lana School of Public Health, University of Toronto, Toronto, Ontario Canada

**Keywords:** Overview of reviews, Insomnia, Sleep disorders, Comparative effectiveness, Knowledge synthesis

## Abstract

**Background:**

This review aimed to assess the existing evidence regarding the clinical effectiveness and safety of pharmacological and non-pharmacological interventions in adults with insomnia and identify where research or policy development is needed.

**Methods:**

MEDLINE, Embase, PsycINFO, The Cochrane Library, and PubMed were searched from inception until June 14, 2017, along with relevant gray literature sites. Two reviewers independently screened titles/abstracts and full-text articles, and a single reviewer with an independent verifier completed charting, data abstraction, and quality appraisal.

**Results:**

A total of 64 systematic reviews (35 with meta-analysis) were included after screening 5024 titles and abstracts and 525 full-text articles. Eight of the included reviews were rated as high quality using the Assessment of Multiple Systematic Reviews 2 (AMSTAR2) tool, and over half of the included articles (*n* = 40) were rated as low or critically low quality. Consistent evidence of effectiveness across multiple outcomes based on more than one high- or moderate quality review with meta-analysis was found for zolpidem, suvorexant, doxepin, melatonin, and cognitive behavioral therapy (CBT), and evidence of effectiveness across multiple outcomes based on one high-quality review with meta-analysis was found for temazepam, triazolam, zopiclone, trazodone, and behavioral interventions. These interventions were mostly evaluated in the short term (< 16 weeks), and there was very little harms data available for the pharmacological interventions making it difficult to evaluate their risk-benefit ratio.

**Conclusions:**

Assuming non-pharmacological interventions are preferable from a safety perspective CBT can be considered an effective first-line therapy for adults with insomnia followed by other behavioral interventions. Short courses of pharmacological interventions can be supplements to CBT or behavioral therapy; however, no evidence regarding the appropriate duration of pharmacological therapy is available from these reviews.

**Systematic review registration:**

PROSPERO CRD42017072527.

## Background

Insomnia is a common disorder in the general population. While precise estimates vary, multiple population-based studies in different countries have consistently found that approximately one third of adults (> 18 years of age) reported dissatisfaction with their sleep and at least one symptom of insomnia [1, 2] and 6–10% of the adult population met stricter criteria for a diagnosis of insomnia such as the Diagnostic and Statistical Manual of Mental Disorders (DSM-5) [3] or International Classification of Sleep Disorders (ICSD) [4]. Insomnia can contribute to significant functional impairments at work or at home and is linked to reduced quality of life, problems with attention and memory, mood disturbances, and reduced ability to carry out normal daily activities [5]. Furthermore, studies have indicated that insomnia may be an important risk factor for the onset of mental health disorders such as depression, anxiety, and substance abuse [5].

Clinical practice guidelines published in the USA, Canada, and Europe unanimously recommend that non-pharmacological approaches, especially cognitive behavioral therapies, should be the first-line treatment for chronic insomnia (symptoms for > 3 months) and that pharmacological treatment should only be used in acute cases (< 3 months) or as a short-term supplement to non-pharmacological approaches [6–8]. Evidence for over-the-counter (e.g., diphenhydramine) or natural remedies (melatonin, valerian) is considered weak or inconclusive, and these approaches are not recommended for acute or chronic insomnia [6–8]. Despite this, the rate of prescription sleep aid use, particularly non-benzodiazepines and off-label use of antidepressants, has risen significantly over the last 20 years [9–11], in some cases outpacing the diagnosis of sleep disorders among the general population [10]. Furthermore, a large prospective study of former and current insomnia sufferers found that 70% of patients using a prescription sleep aid continued to do so at 1-year follow-up but did not demonstrate significant improvements in sleep compared to non-users [12]. The use of non-prescription sleep aids is also common alongside prescription drugs; up to 60% of sleep aids used by adults with insomnia are non-prescription [12, 13].

Evidence is needed to support the development of guidelines that encourage the appropriate use of pharmacological interventions to treat insomnia and increase access to and uptake of non-pharmacological approaches. The objective of this overview of systematic reviews was to assess what has been established regarding the clinical effectiveness and safety of pharmacological and non-pharmacological interventions in adults with insomnia and identify areas where further research or policy development is needed.

## Methods

### Protocol

This overview was commissioned by the Canadian Agency for Drugs and Technologies in Health (CADTH) as part of an assessment of the management of insomnia in adults in Canada. In accordance with guidance from the Cochrane Handbook, a protocol for the overview of systematic reviews was written a priori by the research team in consultation with the project owner and other stakeholders. The protocol was registered with the PROSPERO database (CRD42017072527) [14] and the full version can be found in Additional file 1. Results are reported using the Preferred Reporting Items for Overviews of Systematic Reviews Including Harms (PRIO-harms) checklist (Additional file 2: Appendix A) [15]. As the methods have been reported fully in our report that was produced for CADTH [16], they are outlined briefly here.

### Eligibility criteria

Eligibility criteria for the overview were established using the Population, Intervention, Comparator, Outcome, and Study design (PICOS) framework to include the following:

- Patients: adults > 18 years of age diagnosed with acute (< 3 months) or chronic (> 3 months) insomnia disorder according to the DSM diagnostic criteria, International Classification of Sleep Disorders, or Research Diagnostic Criteria for insomnia [17].
- Interventions: prescription or non-prescription pharmacological interventions used to treat insomnia approved for use or under review for approval in Canada; non-pharmacological interventions included cognitive behavioral therapy, sleep restriction, relaxation, meditation, etc.; or a combination of pharmacological and non-pharmacological interventions. Herbal remedies or complementary and alternative medicine (CAM) were ineligible; exceptions were made for melatonin and mindfulness-based therapies as they were of special interest to stakeholders.
- Comparator: inactive controls (e.g., placebo, wait-list control, self-monitoring) or active controls (e.g., another eligible intervention).
- Outcomes:
- Effectiveness: sleep onset latency (SOL), total sleep time (TST), wake after sleep onset (WASO), sleep quality (SQ), sleep satisfaction (SS), sleep efficiency (SE), Insomnia Severity Index (ISI) scores, fatigue severity, and health-related quality of life (HrQoL)
- Harms: hangover/morning sedation, accidental injuries, additional healthcare use related to harms of the intervention, delirium related to the intervention, sleep disordered breathing related to the intervention, addiction, dependence, or diversion of medications (A/D/D), and all-cause mortality related to the intervention
- Study design: systematic knowledge syntheses including primary studies of any design with or without a meta-analysis, using the Cochrane Collaboration definition [18]. Reviews were required to report that a literature search was carried out in at least one database in order to be eligible; articles identified as rapid reviews, literature reviews, narrative reviews, or other non-systematic knowledge syntheses were excluded from the overview.
- Other: Published or unpublished systematic reviews were eligible for inclusion, as well as publications in any language.

### Literature search

Published literature was identified by searching MEDLINE, Embase, PsycINFO, The Cochrane Library, and PubMed from inception until June 14, 2017. The search strategy contained both controlled vocabulary (MeSH terms) and relevant keywords (e.g., insomnia, sleep initiation disorder), and a methodological filter was applied to limit the search to systematic reviews and meta-analyses. No date or language restrictions were applied. The search strategy was developed by an experienced librarian (BS) and peer-reviewed by another librarian (SJ) using the PRESS Checklist [19]; searches were carried out by an experienced information specialist (AE); the full search strategy is available in Additional file 2: Appendix B. Unpublished (or gray) literature was identified by searching sites based on the *Gray Matters* checklist [20]; the full list is available in Additional file 2: Appendix B. The literature search was supplemented by reviewing the bibliographies of the included reviews and other key papers, as well as contacting the authors of relevant conference abstracts and review protocols for manuscripts or unpublished data.

### Study selection and data abstraction

Calibration exercises were completed with the review team prior to level 1 (title/abstract) and level 2 (full-text) screening, the charting exercise, and data abstraction to ensure reliability of the processes and revise forms as needed. Only one round of calibration using 25 citations was required prior to level 1 screening (> 75% agreement), charting (5 articles), and data abstraction (6 articles), while two rounds of calibration (> 75% agreement) were required prior to level 2 screening (15 and 25 articles, respectively). Level 1 and 2 screening was completed in duplicate by pairs of reviewers working independently and any discrepancies were resolved by a third reviewer, and charting and data abstraction were completed by a single reviewer and verified by a second. Screening was completed using synthesiSR, proprietary online software developed by the Knowledge Translation Program of St. Michael’s Hospital [21].

A charting exercise was completed prior to data abstraction to collect information on review characteristics, particularly how outcomes were reported and which outcome measures were used in the included reviews. Data abstraction items included review characteristics (e.g., year of conduct/literature search, type of included study designs), patient characteristics (e.g., type and number of patients, age mean, and standard deviation), interventions examined (e.g., type of intervention, dose/frequency), and outcomes examined (e.g., name of outcome, outcome measure/definition). A list of the primary studies included in all of the systematic reviews with meta-analysis (SR + MAs) was compiled and cross-referenced with the primary studies included in the SRs. Any SRs that completely overlapped with the primary studies included in the abstracted SR + MAs (e.g., did not contribute any new evidence) were excluded from the overview.

### Quality appraisal and assessment of evidence

Quality appraisal was completed concurrently with data abstraction using the Assessing the Methodological Quality of Systematic Reviews tool version 2 (AMSTAR2) [22]. The tool was tested in the same calibration exercises as the data abstraction form and assessments were completed by one reviewer and verified by a second. Additionally, a GRADE algorithm developed for Cochrane overviews of reviews was used to ascertain the strength of evidence of the reviews included in each treatment comparison for all outcomes [23]. In this algorithm, each review starts with a ranking of high certainty and is downgraded 1 level for serious methodological concerns (sample size between 100 and 199 participants; high risk of bias in randomization and blinding for > 75% included studies; high heterogeneity (*I*^2^ > 75%); and “No” on one of these AMSTAR2 items: a priori research design, comprehensive literature search, duplicate study selection, or duplicate study abstraction) or 2 levels for very serious concerns (sample size < 100 participants and “No” on two or more of these AMSTAR2 items: a priori research design, comprehensive literature search, duplicate study selection, or duplicate study abstraction) [23].

### Data synthesis

No formal statistical analysis was planned for this overview as substantial clinical and methodological heterogeneity was expected across the included reviews and pooling the data or conducting an indirect comparison would not be appropriate in this situation. Lists of the primary studies in each included review were collated and cross-referenced in a matrix of evidence tables to ascertain the degree of overlap between reviews for each treatment comparison and outcome to provide context for the results. Additionally, a matrix of evidence for the entire overview was prepared and used to calculate the “corrected covered area” (CCA) to quantify the degree of overlap between all of the reviews included in this work [24].

### Patient and public involvement

Patients and/or public were not involved in the development, design, or conduct of this research.

## Results

### Literature search

**Fig. 1:**
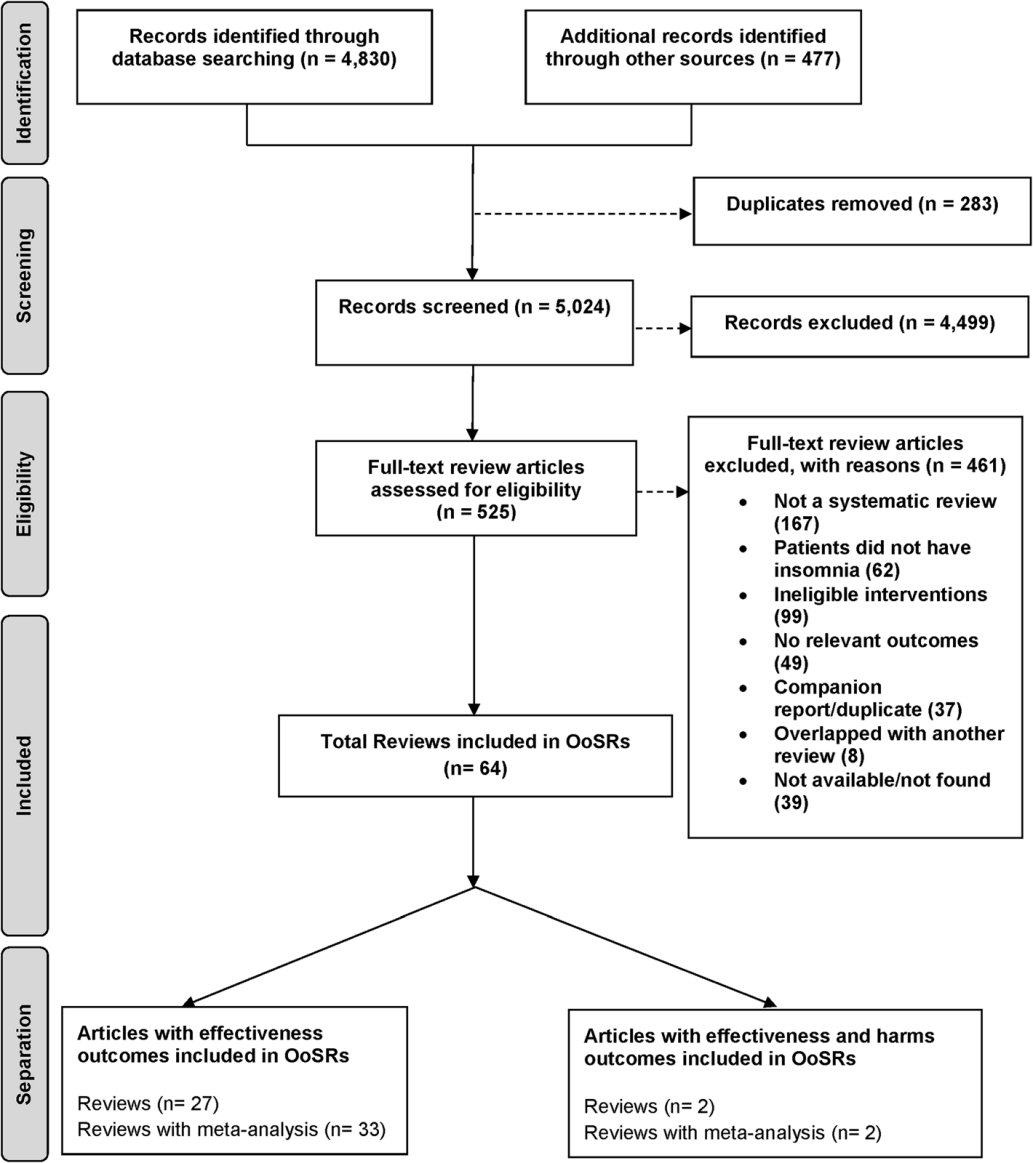
Flow chart for overview of systematic reviews (OoSRs)

The literature search resulted in 5024 titles and abstracts to be screened after de-duplication, 4499 of which were excluded after level 1 screening for not meeting eligibility criteria (Fig. 1). A total of 525 full-text articles were retrieved for screening at level 2 where a further 312 articles were excluded, leaving 213 articles eligible for data abstraction (the list of excluded studies is available upon request). After completion of the charting exercise and data abstraction, a total of 64 articles, 34 published SR + MAs [25–58] and one unpublished SR + MA (Dr. Hae Sun Suh, unpublished data 2018) and 29 SRs [59–87], were included in this overview. A total of 358 index publications (primary studies) were cited 612 times across the 64 SR + MAs and SRs included in this overview; resulting in a CCA of 0.011 indicating little to no overlap across the included reviews.

### Review characteristics

The included reviews were conducted between 1997 and 2017 with the majority (75%) published after 2010 (Table 1; Additional file 2: Appendix C). Literature search dates for the included reviews ranged from 1996 to 2016 with more than half (62%) being conducted after 2010 (Table 1; Additional file 2: Appendix C). Only 11 (17%) of the included reviews searched databases from inception, and a further 5 (7%) reviews ran searches going back more than 50 years. The first authors of the SR + MAs were predominantly based in Asia (43%), specifically China (7/35), while the majority of SR authors were based in North America (65%), predominantly in the US (17/29). An average of 27 primary studies (range 3–139) were included in the SR + MAs, and an average of 8 primary studies (2–22) were included in the SRs. Randomized controlled trials were the most commonly included primary study design, appearing in 33 SR + MAs (94%) and 23 SRs (79%). Non-randomized controlled trials (NRCTs) were the next most common (7 SRs, 24%) followed by quasi-experimental study designs (1 SR + MA, 3%; 3 SRs, 10%) and observational studies (4 SRs, 14%). Two SR + MAs, and 4 SRs did not report the specific study designs included for review.

**Table 1:** Summary of review and participant characteristics

Characteristic	Systematic reviews with meta-analysis (*n* = 35)	Systematic reviews without meta-analysis (*n* = 29)
Review characteristics		
Year of publication [*n* (%)]		
1990–1999	1 (3)	1 (3)
2000–2009	5 (14)	9 (31)
2010–2018	29 (83)	19 (66)
Literature search date [*n* (%)]		
1990–1999	1 (3)	2 (7)
2000–2009	7 (20)	7 (24)
2010–2019	24 (69)	16 (55)
Not reported	3 (9)	4 (14)
Literature search coverage [*n* (%)]		
From database inception	8 (23)	3 (10)
> 50 years prior to search date	2 (6)	3 (10)
30–49 years prior to search date	4 (11)	9 (31)
10–29 years prior to search date	1 (3)	1 (3)
Not reported	20 (57)	13 (45)
Region of publication [*n* (%)]		
Asia	15 (43)	2 (7)
Europe	8 (23)	6 (21)
North America	11 (31)	19 (65)^a^
Oceania	1 (3)	2 (7)
Number of included primary studies [mean (range)]	27 (3–139)	8 (2–22)
Included study designs [*n* (%)]^b^		
Randomized controlled trials	33 (94)	23 (79)
Non-randomized controlled trials	–	7 (24)
Quasi-experimental	1 (3)	3 (10)
Observational	–	4 (14)
Not reported	2 (6)	4 (14)
Population characteristics		
Overall sample size [mean (range)]^c^	1861 (171–6303)	566 (34–1794)
Mean age [range (years)]^d^	45–56.6	53.3^c^
Proportion of female participants [range (%)]^e^	35.6–74.2	–
Patients with co-morbidities [*n* (%)]		
No co-morbidities	12 (34)	6 (21)
Mix of patients with and without co-morbidities	9 (26)	9 (31)
All patients with co-morbidities	11 (31)	9 (31)
Not reported	3 (9)	5 (17)
Types of co-morbidities [*n* (%)]^b^		
Mental health conditions (various)	9 (26)	11 (38)
Cancer	8 (23)	6 (21)
Conditions causing chronic pain	5 (14)	–
Chronic illness (unspecified)	4 (11)	2 (7)
Other sleep disorders	4 (11)	–
Dementia	3 (9)	2 (7)
Physical disability	2 (6)	1 (3)
Treatment comparisons [*n* (%)]^b^ (v inactive controls) (v active controls)		
Benzodiazepines (flurazepam, temazepam, triazolam)	3 (9) --	2 (7) 1 (3)
Non-benzodiazepine receptor agonists (zolpidem, zopiclone)	4 (11) --	5 (17) 3 (10)
Suvorexant	3 (9) --	1 (3) --
Antidepressants (doxepin, trazodone)	5 (14) --	5 (17) 3 (10)
Antipsychotics (quetiapine)	-- --	4 (14) --
Melatonin	8 (23) --	4 (14) --
Diphenhydramine	1 (3) --	2 (7) --
Cognitive behavioral therapy (CBT) (CBT, CBT + behavioral intervention, multi-component CBT)	20 (57) 3 (9)	8 (28) 3 (10)
Behavioral interventions (relaxation, sleep restriction, multi-component behavioral)	4 (11) 1 (3)	7 (24) --
Mindfulness-based interventions	1 (3) --	-- --
Combination therapies (CBT + pharmacotherapy)	1 (3) --	1 (3) --

^a^Includes 17 SRs from the USA

^b^SR + MAs or SRs can contribute to multiple categories, totals will not equal 100%

^c^Sample size was not reported in 11 SR + MAs and 8 SRs

^d^Mean age was only reported in 1 SR and 7 SR + MAs

^e^The percentage of female patients was only reported in 7 SR + MAs

### Study and patient characteristics

The overall sample size was reported in 24/35 SR + MAs and 21/29 SRs, averaging 1861 patients (range 171–6303) and 566 patients (34–1794), respectively. Other population characteristics such as mean age and the proportion of female participants appeared in only 7 SR + MAs and 1 SR. The majority of included reviews included patients with insomnia and another co-morbid condition (20 SR + MAs, 57%; 18 SRs, 62%), 12 SR + MAs (34%), and 6 SRs (21%) included patients with insomnia alone; 3 SR + MAs (9%) and 5 SRs (17%) did not report on the presence or absence of co-morbidities in the patient population (Table 1; Additional file 2: Appendix C).

### Interventions and outcomes

The included SR + MAs and SRs examined a total of 32 different treatment comparisons across 11 different classes of interventions. All of the reported interventions were compared with at least one kind of inactive control (e.g., placebo/sham intervention, wait-list, symptom monitoring), and 8 of the reported interventions were compared with an active control (e.g., another eligible intervention—Table 2; Additional file 2: Appendix C).

**Table 2:** Active treatment comparisons

	Triazolam	Flurazepam	Temazepam	Zolpidem	Relaxation therapy	CBT	CBT + relaxation	Multi-component CBT
Zolpidem	1 SR			1 SR				
Zopiclone	1 SR	1 SR	1 SR	1 SR				
Trazodone				3 SR				
CBT					1 SR	2 SR + MA 1 SR	1 SR	
CBT + relaxation					1 SR + MA	1 SR + MA		
Multi-component CBT						2 SR + MA		
CBT + temazepam						1 SR		
Behavioral Therapy								1 SR + MA

*Abbreviations*: *CBT* cognitive behavioral therapy, *SR* systematic review, *SR + MA* systematic review with meta-analysis

Relevant SR + MAs or SRs that examined at least one eligible intervention could be identified for all of the effectiveness outcomes, but relevant SR + MAs or SRs could only be identified for three of the harms outcomes: hangover or morning sedation, accidental injuries, and addiction, dependence, or diversion related to an intervention.

### Quality appraisal and strength of evidence results

**Fig. 2:**
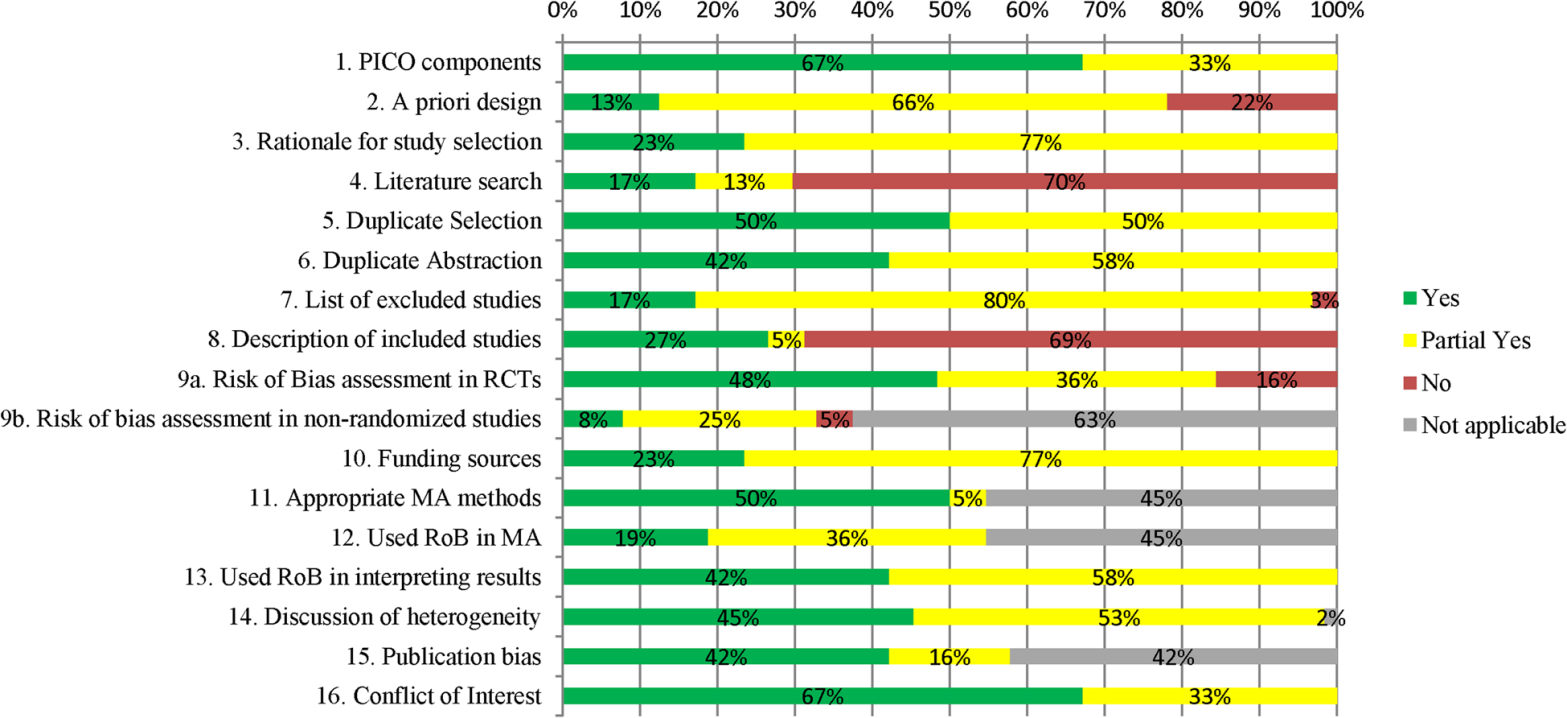
AMSTAR2 results

Only six SR + MAs (20%) and two SRs (7%) were rated as high quality using the AMSTAR 2 tool, and the majority were rated as moderate quality (11 SR + MAs, 31%; 5 SRs, 17%), low quality (8 SR + MAs, 23%; 5 SRs, 17%), or critically low quality (10 SR + MAs, 29%; 17 SRs, 59%; Fig. 2). The full AMSTAR2 results are available in Additional file 2: Appendix D.

Out of the 11 classes of interventions included in this review, only two comparisons (melatonin compared to inactive controls and CBT compared to inactive controls) included reviews rated with a high strength of evidence based on GRADE and nine comparisons (benzodiazepines, non-benzodiazepines, suvorexant, antidepressants, melatonin, CBT, behavioral interventions, and mindfulness-based interventions all compared to inactive controls; and CBT compared to active controls) included reviews rated with a medium strength of evidence (Table 3). Five comparisons included in this overview (antipsychotics, diphenhydramine, and combination therapies all compared to inactive controls; non-benzodiazepines and antidepressants compared to active controls), only included reviews rated as having a low or very low strength of evidence based on GRADE (Table 3).

**Table 3:** Summary of evidence across outcomes from SR + MAs and SRs

Intervention and comparator	Number of primary studies	Type of publication	Findings^a^ and certainty of evidence (GRADE)			
			High^b^	Medium^c^	Low^d^	Very low^e^
Outcome: sleep onset latency (SOL)						
Benzodiazepines vs inactive controls	54	SR + MA		+ 1	+ 1	+ 1
Non-benzodiazepines vs inactive controls	59	SR + MA		+ 2	+ 2	
		SR			+ 2	− 1
Non-benzodiazepines vs active controls	1	SR				− 1
Suvorexant vs inactive controls	3	SR + MA		+ 3		
		SR			+ 1	
Antidepressants vs inactive controls	21	SR + MA		+/− 2	+ 1	
		SR		+/− 1	+/− 2	+/− 1
Antidepressants vs active controls	1	SR			+ 3	
Antipsychotics vs inactive controls	5	SR + MA				
		SR			+ 1	+ 2
Melatonin vs inactive controls	24	SR + MA		+ 3		+ 1
		SR			+ 1/− 1	+/− 1
Diphenhydramine vs inactive controls	5	SR + MA			+/− 1	
		SR			+/− 2	
Cognitive behavioral therapy vs inactive controls	89	SR + MA	+ 3	+ 6	+ 6	− 2
		SR		+ 5	+ 2	+ 1
Cognitive behavioral therapy vs active controls	7	SR + MA			+ 1/− 1	
		SR				
Behavioral Interventions vs inactive controls	22	SR + MA			− 2	
		SR		+ 1	+ 1	
Mindfulness-based Interventions vs inactive controls	2	SR + MA		+ 1		
Combination Therapies vs inactive controls	1	SR			+ 1	
Outcome: total sleep time (TST)						
Benzodiazepines vs inactive controls	33	SR + MA			+ 1	+ 1
		SR				+ 1
Non-benzodiazepines vs inactive controls	41	SR + MA		+ 2	+ 1	
		SR			+ 1	+ 1
Non-benzodiazepines vs active controls	1	SR				+ 1
Suvorexant vs inactive controls	3	SR + MA		+ 2	+ 1	
		SR			+ 1	
Antidepressants vs inactive controls	24	SR + MA		+ 4		
		SR		+/− 1	+/− 2	+/− 1
Antidepressants vs active controls	1	SR			− 2	
Antipsychotics vs inactive controls	4	SR				+/− 3
Melatonin vs inactive controls	30	SR + MA	− 1	+ 2/− 1	− 1	− 1
		SR		+ 1	+/− 1	+/− 2
Diphenhydramine vs inactive controls	4	SR + MA			− 1	
		SR			+/− 2	
Cognitive behavioral therapy vs inactive controls	72	SR + MA	+ 1/− 1	+ 4/− 2	+ 1/− 4	+ 1/− 1
		SR		+/− 2	+/− 1	
Cognitive behavioral therapy vs active controls	2	SR + MA			− 2	
		SR		− 1		
Behavioral Interventions vs inactive controls	7	SR + MA			− 1	
		SR			− 1	− 1
Mindfulness-based Interventions vs inactive controls	2	SR + MA		− 1		
Combination therapies vs inactive controls	4	SR + MA			− 1	
		SR			+ 1	
Outcome: wake after sleep onset (WASO)						
Benzodiazepines vs inactive controls	4	SR + MA			+ 1	
Non-benzodiazepines vs inactive controls	16	SR + MA		− 1	+ 1	
		SR			+ 2	+ 1
Non-benzodiazepines vs active controls	3	SR + MA				
		SR			+ 1	
Suvorexant vs inactive controls	3	SR + MA		+ 1	+ 1	
		SR			+ 1	
Antidepressants vs inactive controls	13	SR + MA		+ 2		
		SR		+ 1	+ 2	+ 2
Antidepressants vs active controls	1	SR			+ 2	
Melatonin vs inactive controls	13	SR + MA		− 1	− 2	
		SR				+ 2
Diphenhydramine vs inactive controls	1	SR				− 1
Cognitive behavioral therapy vs inactive controls	70	SR + MA	+ 1/− 1	+ 5	+ 7/− 2	+ 1
		SR		+ 3	+ 3	
Cognitive behavioral therapy vs active controls	17	SR + MA			− 2	
		SR		+ 1		+ 1
Behavioral Interventions vs inactive controls	9	SR + MA		+/− 1	+/− 1	
		SR			+ 1	
Outcome: sleep quality						
Benzodiazepines vs inactive controls	2	SR + MA			− 1	
Non-benzodiazepines vs inactive controls	9	SR + MA		+ 1	+ 1	
		SR			+ 2	
Suvorexant vs inactive controls	3	SR + MA		+ 1		
Antidepressants vs inactive controls	13	SR + MA		+ 1		
		SR			+ 3	
Antidepressants vs active controls	1	SR			− 1	
Antipsychotics vs inactive controls	8	SR			+ 2	+ 2
Melatonin vs inactive controls	23	SR + MA		− 3	+ 1/− 1	
		SR			+/− 3	+ 2
Diphenhydramine vs inactive controls	1	SR				− 1
Cognitive behavioral therapy vs inactive controls	47	SR + MA		+ 5/− 1	+ 5/− 2	+ 1
		SR		+ 1	+ 2/− 1	
Cognitive behavioral therapy vs active controls	4	SR			− 2	
Behavioral Interventions vs inactive controls	12	SR + MA			+ 1	
		SR		+ 1/− 1	+ 3/− 1	
Behavioral Interventions vs active controls	8	SR + MA			+ 1	
Mindfulness-based Interventions vs inactive controls	4	SR + MA		+ 1		
		SR				+ 1
Combination Therapies vs inactive controls	1	SR			+ 1	
Outcome: sleep satisfaction						
Antipsychotics vs inactive controls	1	SR				− 1
Melatonin vs inactive controls	1	SR			+ 1	
Cognitive behavioral therapy vs inactive controls	2	SR		+ 1		
Behavioral Interventions vs inactive controls	1	SR			+ 1	
Outcome: sleep efficiency						
Non-benzodiazepines vs inactive controls	4	SR + MA		+ 1		
		SR				+/− 1
Non-benzodiazepines vs active controls	2	SR				+/− 1
Antidepressants vs inactive controls	15	SR + MA		+ 2		
		SR		+ 1	+ 1	+/− 2
Antipsychotics vs inactive controls	2	SR				+ 3
Melatonin vs inactive controls	19	SR + MA	− 1	+ 1/− 2	− 1	
		SR			+/− 1	+ 1/− 1
Diphenhydramine vs inactive controls	2	SR			+/− 1	
Cognitive behavioral therapy vs inactive controls	83	SR + MA	+ 1	+ 7/− 1	+ 5	+ 1
		SR		+ 4	+ 5	
Cognitive behavioral therapy vs active controls	4	SR + MA			− 2	
		SR		+ 1	+ 1	
Behavioral Interventions vs inactive controls	4	SR			+ 2	
Mindfulness-based Interventions vs inactive controls	5	SR + MA		− 1		
		SR			+/− 1	
Outcome: Insomnia Severity Index (ISI)						
Suvorexant vs inactive controls	3	SR + MA		+ 1	+ 1	
		SR			+ 1	
Antidepressants vs inactive controls	2	SR + MA		+ 1		
Antipsychotics vs inactive controls	1	SR				+ 1
Diphenhydramine vs inactive controls	1	SR			+ 1	
Cognitive behavioral therapy vs inactive controls	46	SR + MA		+ 4/− 1	+ 5	
		SR		+ 3	+ 2	
Cognitive behavioral therapy vs active controls	2	SR + MA			− 1	
Outcome: fatigue severity						
Cognitive behavioral therapy vs inactive controls	26	SR + MA	+/− 1	+/− 2		
		SR		+ 2		
Behavioral Interventions vs inactive controls	2	SR			− 1	
Outcome: health-related quality of life						
Non-benzodiazepines vs inactive controls	4	SR		+/− 1		
Melatonin vs inactive controls	1	SR				+ 1
Cognitive behavioral therapy vs inactive controls	7	SR		+ 2	+ 2	
Cognitive behavioral therapy vs active controls	1	SR			+ 1	
Outcome: hangover/morning sedation						
Non-benzodiazepines vs inactive controls	1	SR				− 1
Non-benzodiazepines vs active controls	1	SR				− 1
Suvorexant vs inactive controls	3	SR + MA		+ 2		
Antipsychotics vs inactive controls	2	SR				+ 1
Outcome: accidental injury						
Suvorexant vs inactive controls	3	SR + MA		+ 2		
Outcome: addiction, dependence, or diversion						
Non-benzodiazepines vs inactive controls	3	SR			+/− 1	
Non-benzodiazepines vs active controls	3	SR			+/− 1	
Suvorexant vs inactive controls	3	SR + MA		+ 2		

*Abbreviations*: *SR* systematic review, *SR + MA* systematic review and meta-analysis, *GRADE* Grading of Recommendations Assessment, Development and Evaluation

^a^The number indicates how many SR + MAs or SRs contributed to that result

^b^Reviews that received no downgrades

^c^Reviews that received 1 or 2 downgrades

^d^Reviews that received 3 or 4 downgrades

^e^Reviews that received 5 or 6 downgrades

^+^Signifies statistically significant improvement in effectiveness outcomes or significantly increased risk of harms for safety outcomes

^−^Signifies non-statistically significant change

^+/−^Signifies mixed or discordant results within SR + MAs and SRs

### Outcome results

All of our results have been transparently reported in our report for CADTH that is available on their website [16], as well as in Additional file 2: Tables E1-E11 Appendix E. To focus our results for this publication, only the statistically significant results from SR + MAs are included in the text. For outcomes where no evidence from SR + MAs could be identified, positive results from individual studies included in relevant SRs are reported. Tables with the overlap in the primary studies included in the SRs and SR + MAs can be found in Tables F1-F11 Additional file 2: Appendix F and in Additional file 3.

#### Benzodiazepines

One high-quality SR + MA [26] compared flurazepam to placebo and found improvements in SOL (10 RCTs, 532 patients) compared to placebo (Table 3; Table E1 Additional file 2: Appendix E). One high-quality [26] and one critically low-quality [45] SR + MA compared temazepam to placebo and found statistically significant improvements in SOL (2 RCTs, 72 patients), TST (2 RCTs, 72 patients), WASO (2 RCTs, 77 patients), and SQ (2 RCTs, 78 patients; Table 3; Table E1 Appendix E, Table F1 Additional file 2: Appendix F). One high-quality [26] and one critically low-quality [48] SR + MA compared triazolam to placebo and found significant improvements in SOL (8 RCTs, 539 patients and 28 RCTs, sample size not reported [NR]), TST (12 RCTs, sample size NR), and WASO (2 RCTs, 57 patients; Table 3).

#### Non-benzodiazepine receptor agonists

Two high-quality [26, 41] and two critically low-quality [45, 48] SR + MAs compared zolpidem to placebo and found improvements in SOL (5 to 29 RCTs, 355 to 1805 patients), TST (2 to 23 RCTs, 112 to 890 patients), WASO (8 RCTs, 896 patients), SQ (3 RCTs, 557 patients and 6 RCTs, 638 patients), and SE (4 RCTs, 226 patients; Table 3; Table E2 Appendix E, Table F2 Additional file 2: Appendix F). Also, one critically low-quality SR [73] compared nightly zolpidem doses to zolpidem “as needed” and found an increase in HRQoL for both groups (1 study, 789 patients; Table 3; Table E2 Additional file 2: Appendix E). One critically low-quality SR [79] compared zolpidem to triazolam and found improvements in TST (1 study, 16 patients), WASO (3 studies, 102 patients), and SE (2 studies, 86 patients; Table 3; Table E2 Additional file 2: Appendix E). One high-quality [26] and one critically low-quality [48] SR + MA compared zopiclone to placebo and found improvements in SOL (5 RCTs, 356 patients and 15 RCTs, sample size NR), and TST (13 RCTs, sample size NR). One critically low-quality SR [65] compared zolpidem, zopiclone, triazolam, temazepam, and placebo and found slightly increased risks of dependency or withdrawal symptoms in patients taking zopiclone compared to the other medications (7 studies, 450 patients; Table 3; Table E2 Additional file 2: Appendix E).

#### Suvorexant

One high-quality [41] and two moderate quality [36, 38] SR + MAs compared suvorexant to placebo and found improvements in SOL, TST, WASO, SQ, and ISI scores as well as increased risks of hangover or morning sedation effects, accidental injury, and addiction or dependence (Table 3; Table E3 Appendix E, Table F3 Additional file 2: Appendix F).

#### Antidepressants

Two high-quality [26, 41], one low-quality [56], and two critically low-quality [39, 45] SR + MAs compared doxepin to placebo and found improvements in SOL (2 to 3 RCTs, 60 to 415 patients), TST (2 to 7 RCTs, 60 to 1476 patients), WASO (2 to 4 RCTs, 60 to 558 patients), SQ (2 RCTs, 291 patients and 2 RCTs, 404 patients), SE (2 to 3 RCTs, 60 to 425 patients), and ISI scores (2 RCTs, 494 patients; Table 3; Additional file 2: Appendix E, Table E4). One high-quality SR + MA [26] and four critically low-quality SRs [74, 75, 77, 82] compared trazodone to placebo and found improvements in SOL (2 RCTs, 208 patients), TST (1 to 5 studies, 39 to 323 patients), WASO (1 to 2 studies, 15 to 306 patients), SQ (1 to 5 studies, 9 to 767 patients), and SE (2 to 3 studies, 20 to 56 patients; Table 3; Additional file 2: Appendix E, Table E4). Three critically low-quality SRs [75, 77, 82] all reported on the same RCT that compared trazodone and zolpidem to placebo (306 patients) and only found greater improvements in SOL for patients in the zolpidem group (Table 3; Table E4, Additional file 2: Appendix E and Table F4, Appendix F).

#### Antipsychotics

Four critically low-quality SRs [59, 67, 74, 86] compared quetiapine to placebo and found improvements in SOL (2 studies, 52 patients and 2 studies, 32 patients), TST (1 study 18 patients), SQ (1 to 3 studies, 18 to 84 patients), SE (1 study, 18 patients and 1 study, 27 patients), and ISI scores (1 study, 6 patients) as well as increased risk of hangover or morning sedation effects compared to placebo (2 studies, sample size NR; Table 3; Additional file 2: Table E5 Appendix E, Table F5, Appendix F).

#### Melatonin

Three high-quality [26, 27, 40], one moderate quality [53], three published critically low-quality [29, 45, 58], and one unpublished critically low-quality (Dr. Hae Sun Suh, unpublished data 2018) SR + MAs compared melatonin to placebo and found improvements in SOL (8 to 12 RCTs, 206 to 346 patients), TST (8 RCTs, 497 patients and 11 RCTs, sample size NR), and SQ (14 RCTs, sample size NR; Table 3; Additional file 2: Table E6 Appendix E, Table F6, Appendix F). Additionally, one critically low-quality SR [84] compared melatonin to placebo and found improvements in SS (1 study, 112 patients) and HRQoL (1 study, 42 patients).

#### Diphenhydramine

Two critically low-quality SRs [69, 82] compared diphenhydramine to placebo and found improvements in SOL (3 studies, 226 patients and 4 studies, 332 patients), SE (1 study, 204 patients), and ISI scores (1 study, 184 patients; Table 3; Table E7, Additional file 2: Appendix E, Table F7, Appendix F).

#### Cognitive behavioral therapy

Four high-quality [25, 26, 41, 42], seven moderate quality [35, 43, 49–51, 55, 57], five low-quality [28, 31, 32, 47, 52], and three critically low-quality [34, 37, 44] SR + MAs compared CBT to inactive controls (e.g., wait-list control, symptom monitoring) and found improvements in SOL (2 to 108 RCTs, 122 to 2010 patients), TST (2 to 91 RCTs, 59 to 2009 patients), WASO (2 to 71 RCTs, 59 to 1655 patients), SQ (2 to 40 RCTs, 580 to 965 patients), SE (2 to 79 RCTs, 59 to 2009 patients), ISI scores (2 to 38 RCTs, 131 to 1655 patients), and fatigue symptoms (6 to 7 RCTs, 398 to 1098 patients; Table 3; Additional file 2: Table E8 Appendix E, Table F8 Appendix F). Additionally, one moderate quality and one low-quality SR [73] compared CBT to inactive controls and found improvements in HRQoL (1 study, 81 patients and 4 studies, 706 patients; Table 3; Additional file 2: Table E8 Appendix E). One moderate quality SR + MA [51] compared two different delivery methods of CBT and found greater improvements in SOL for self-help CBT compared to in-person CBT (3 RCTs, sample size NR), one moderate quality SR compared CBT to relaxation techniques and found improvements in WASO (1 study, 46 patients), one low-quality SR compared individual CBT to group CBT and found improvements in HRQoL for both groups (1 study, 58 patients), and one critically low-quality SR [76] compared CBT alone to CBT plus temazepam and found improvements in WASO for both group and improvements in SE for the CBT plus temazepam group only (1 study, 78 patients; Table 3; Additional file 2: Table E8 Appendix E, Table F8 Appendix F). Finally, one high-quality [26] and one moderate quality [54] SR + MA compared CBT plus relaxation techniques to inactive controls and found improvements for SOL (4 RCTs, 101 patients and 1 RCT, 26 patients) and SQ (3 RCTs, 184 patients; Table 3; Additional file 2: Table E8 Appendix E, Table F8 Appendix F).

#### Behavioral interventions

One high-quality [41] and one critically low-quality [33] SR + MA compared behavioral therapy or brief behavioral interventions to inactive controls (unspecified) and found improvements in SOL (3 RCTs, 146 patients), WASO (3 studies, 146 patients), and SQ (5 studies, sample size NR; Table 3; Additional file 2: Table E9 Appendix E, Table F9 Appendix F). Additionally, one critically low-quality SR [76] compared sleep restriction to inactive controls and found improvements in SE (2 studies, 129 patients; Table 3; Additional file 2: Table E9 Appendix E).

#### Mindfulness

One low-quality SR + MA [30] and one critically low-quality SR [83] compared mindfulness-based interventions (stress reduction, meditation) to inactive controls (wait-list, symptom monitoring, sleep hygiene education) and found improvements in SOL (2 studies, 83 patients), SQ (2 studies, 83 patients), and SE (3 studies, 205 patients; Table 3; Additional file 2: Table E10 Appendix E, Table F10 Appendix F).

#### Combination therapy

One low-quality SR [64] examined mindfulness-based cognitive therapy plus pharmacotherapy (unspecified) and found improvements in TST (mindfulness + pharmacotherapy; 2 studies, 30 patients) and SQ (1 study, 14 patients) compared with baseline values (Table 3; Additional file 2: Table E11 Appendix E, Table F11 Appendix F).

## Discussion

This comprehensive overview of reviews included 64 systematic reviews representing 358 unique primary studies and found consistent evidence of effectiveness for both pharmacological and non-pharmacological interventions based on data from moderate to high quality SR + MAs. There was evidence of effectiveness across multiple outcomes reported in more than one high- or moderate quality SR + MA for zolpidem, suvorexant, doxepin, and melatonin, and evidence of effectiveness across multiple outcomes reported in one high-quality SR + MA for temazepam, triazolam, zopiclone, and trazodone. Additionally, the evidence for these interventions included reviews rated as having a high (melatonin) or medium (temazepam, triazolam, zolpidem, zopiclone, suvorexant, doxepin, and trazodone) strength of evidence based on GRADE. However, there was very little harms data available for these interventions. There was little to no evidence of effectiveness or no high- or moderate quality evidence available for flurazepam, quetiapine, or diphenhydramine. Moreover, most interventions were studied in the short term (< 12 weeks) and the primary studies included in the reviews tended to have small sample sizes. The lack of harms data and small study sizes are concerning given that a large proportion of the general population are on these medications. Likewise, there was evidence of effectiveness across multiple outcomes reported in multiple high- or moderate quality SR + MAs for CBT and reported in one high-quality SR + MA for BT; there were no high-quality SR + MAs that examined mindfulness-based or combination therapies. The evidence for these interventions also included reviews rated as a high (CBT) or medium (CBT and behavioral therapy) strength of evidence based on GRADE. The studies that examined CBT and BT were often conducted in the short term, and only one SR + MA examined the effect of online versus in-person CBT, which is an important question for future research given the cost of and difficulties accessing in-person CBT [88].

This overview of reviews identified several evidence gaps in the field of insomnia research, particularly the lack of harms data for pharmacological interventions, the effects of different doses, the effectiveness of sequencing or combining drug and non-drug interventions, and a dearth of head-to-head studies directly comparing pharmacological or non-pharmacological interventions. Additionally, the clinical significance of symptomatic changes in insomnia is poorly understood and standards that allow researchers to interpret whether a statistically significant change translates to a clinically significant one are needed (e.g., the minimal clinically important difference).

There are limitations of the included systematic reviews worth noting, particularly the low quality of the included evidence with more than 50% of the included reviews receiving a low- or critically low-quality score on the AMSTAR2 tool. This suggests that substantial improvements in the methods used to synthesize knowledge in this field are needed and that current results should be interpreted with caution. Systematic reviews in this field could be improved by increasing the use of a priori protocols, providing a rationale for including or excluding certain study designs, providing a list of excluded studies with reasons for exclusion, and transparently reporting the funding sources of primary studies included in the review.

There are also some limitations to the conduct of this overview that should be taken into consideration. Due to time and resource constraints, targeted searches for primary studies reporting harms outcomes could not be conducted, which is a deviation from our original protocol [14]. Additionally, although the literature search attempted to find unpublished research and reviews in multiple languages, only one unpublished review and 2 reviews in languages other than English were identified, suggesting that these results are not generalizable beyond systematic reviews published in English. Additionally, the definition of inactive controls used in this overview included standard care interventions such as sleep hygiene and patient education, which may have resulted in underestimation of the effectiveness of some of the non-drug interventions as they were largely compared with these types of controls rather than true control conditions such as placebo or sham interventions. Also, the behavioral, mindfulness and cognitive behavioral interventions included in this review were categorized as reported by review authors. In the interest of capturing a comprehensive evidence base, we did not put any limitations on the eligibility of these interventions leading to a high degree of variability across the reviews. Finally, as stated previously, due to a lack of clinical standards for interpretation, none of the changes in outcomes reported here could be evaluated in terms of their clinical or symptomatic relevance.

There are several strengths of this overview that are worth noting, particularly the use of the Cochrane handbook [18] and an a priori protocol to guide the conduct of the overview, as well as the use of the AMSTAR2 [22] tool for quality appraisal. The literature search was comprehensive and included both published and unpublished sources of information and had no restrictions on publication date or language of publication. The final list of eligible interventions and outcomes was developed in consultation with project stakeholders and clinical experts who were consulted throughout the overview process. Finally, the 64 included systematic reviews were closely examined for overlaps in the primary evidence which was found to be extensive and which we clearly highlighted throughout the “Results” section.

## Conclusions

Based on the results of this overview, clinicians and patients with insomnia can consider CBT as a first-line intervention due to its consistent evidence of effectiveness and a high strength of evidence across multiple outcomes and because it is likely associated with few or no serious harms though there is insufficient evidence to properly evaluate the benefit to harm ratio for this intervention. If CBT is not effective, then other behavioral interventions can be considered or short courses of melatonin, zolpidem, suvorexant, or doxepin can be added to non-pharmacological therapy. However, these agents have only been tested in short-term studies and there is little evidence for their effectiveness or safety beyond 16 weeks of treatment.

## Supplementary information

**Additional file 1.** PROSPERO Registration. The additional file includes the PROSPERO registration for the study.

**Additional file 2.** The appendices include all supplemental data and information. Appendix A: PRIO-harms Checklist. Appendix B: Database Search Strategy and List of Gray Literature search sites. Appendix C: Review, participant, and intervention characteristics. Appendix D: AMSTAR Results. Appendix E: Detailed Tables of Results. Appendix F: Tables of primary studies by treatment comparison for outcomes with more than one included SR or SR + MA.

**Additional file 3.** Matrix of Evidence. The matrix of evidence of primary studies across all included reviews.

## Data Availability

The datasets used and/or analyzed during the current study are available from the corresponding author upon reasonable request.

## Abbreviations

AMSTAR: A Measurement Tool to Assess Systematic Reviews
BT: Behavioral therapy
CBT/CBT-I: Cognitive Behavioral Therapy/Cognitive Behavioral Therapy for Insomnia
ISI: Insomnia Severity Index
MA: Meta-analysis
PSG: Polysomnography
PSQI: Pittsburgh Sleep Quality Index
SE: Sleep efficiency
SOL/SL: Sleep onset latency/sleep latency
SQ: Sleep quality
SR: Systematic review
SS: Sleep satisfaction
TST: Total sleep time
WASO: Wake after sleep onset
