# Supplementary material for: Comparative effectiveness and safety of pharmacological and non-pharmacological interventions for insomnia: an overview of reviews
Source: Syst Rev. 2019 Nov 15;8:281. doi: 10.1186/s13643-019-1163-9 (PMC6857325; doi:10.1186/s13643-019-1163-9)
Supplement: Supplementary file 1 — Additional file 1. PROSPERO Registration. The additional file includes the PROSPERO registration for the study. [file 13643_2019_1163_MOESM1_ESM.pdf]

NHS
National Institute for Health Research

Comparative effectiveness and safety of pharmacological and non-pharmacological interventions for insomnia: an overview of reviews

Andrea Tricco, Patricia Rios, Roberta Cardoso, Deanna Morra, Zahra Goodarzi, Bechara Farah, Sharada Harricharan, Sharon Straus

Citation

Andrea Tricco, Patricia Rios, Roberta Cardoso, Deanna Morra, Zahra Goodarzi, Bechara Farah, Sharada Harricharan, Sharon Straus. Comparative effectiveness and safety of pharmacological and non-pharmacological interventions for insomnia: an overview of reviews. PROSPERO 2017 CRD42017072527 Available from:
http://www.crd.york.ac.uk/PROSPERO/display_record.php?ID=CRD42017072527

Review question

1. What are the comparative efficacies of available interventions for insomnia disorder?
2. What are the long-term safety implications of interventions for insomnia disorder?

Searches

Comprehensive literature searches will be conducted by an experienced librarian (Becky Skidmore) in PubMed, EMBASE, PsycINFO, MEDLINE, and The Cochrane library, and another expert librarian will peer review the search strategy using the PRESS checklist.
The search strategy will consist of both controlled vocabulary, such as the National Library of Medicine's MeSH (Medical Subject Headings) terms, and keywords. For safety, whenever systematic reviews of relevant interventions are not identified, targeted literature searches will be conducted to identify randomized trials and observational studies.
Relevant grey literature (literature that is not published or widely available) will be identified by searching public health websites (e.g., Public Health Agency of Canada, Health Canada), drug regulatory websites (e.g., Food and Drug Administration; FDA), and the PROSPERO systematic review registry. In addition, clinical trial registries (e.g., World Health Organization International Clinical Trials Search Portal, www.clinicaltrials.gov) will also be searched to identify unpublished studies for safety outcomes whenever systematic reviews are not available.
The reference lists of included reviews will also be searched to identify any further reviews of interest.

Types of study to be included

Systematic reviews on the safety of pharmacological and non-pharmacological interventions for insomnia.

Condition or domain being studied

Chronic and acute (<3 months) insomnia.

Participants/population

Adults aged 18 and older with insomnia disorder (e.g., DSM diagnostic criteria, international classification of sleep disorders, and research diagnostic criteria for insomnia) including patients with acute (<3 months), as well as chronic symptoms.
Subpopulations:
• Age groups (18 to 64 years; 65 years and above);
• Patients in long-term care facilities;
• Patients in correctional facilities.

Intervention(s), exposure(s)

Pharmacologic interventions (prescription and non-prescription); non-pharmacologic interventions (e.g., cognitive behavioural therapy [such as group or individual therapy, phone counselling, or self-directed

therapy], sleep hygiene, sleep restriction, sleep consolidation, stimulus control, meditation, mindfulness-based therapies, or relaxation therapies); and combination pharmacologic and non-pharmacologic interventions.

Comparator(s)/control

Placebo, sham intervention, wait-list control, or other interventions within the scope of the review.

Context

Primary outcome(s)

Efficacy outcomes:
Measured with polysomnography (PSG):
1. Sleep onset latency;
2. Total sleep time;
3. Wake after sleep onset.
Measured with tools, scales/questionnaires, diaries:
4. Sleep quality;
5. Sleep satisfaction;
6. Insomnia severity index (ISI);
7. Fatigue severity - using any measure;
8. Health-related of quality of life - measure of daytime functioning.
Safety outcomes:
Short-term/transient outcomes:
1. Hangover/morning sedation.
Safety outcomes recorded after a minimum of 30 days of continuous drug therapy:
2. Accidental injuries (falls, fractures, traffic injuries);
3. Additional healthcare resource use related to safety of the intervention (hospitalizations, ER visits, doctor’s visits);
4. Delirium related to the intervention;
5. Sleep-disordered breathing related to the intervention;
6. Addiction, dependence, diversion;
7. All-cause mortality related to the intervention.

Timing and effect measures
Not applicable.

Secondary outcome(s)

Not applicable.

Data extraction (selection and coding)

We will conduct Level 1 and 2 screening (citations and full-text articles, respectively) of the literature search results using our online Synthesi.SR software (breakthroughkt.ca). Synthesi.SR is a proprietary tool developed by the Knowledge Translation Program used to manage reviews and to complete title/abstract and full-text screening. To ensure reliability in the use of this software, a training exercise will be conducted using the pre-defined eligibility criteria. If a high percentage agreement (>80%) is observed, two team members will proceed to independently screen each title and abstract for inclusion, and after the pilot-testing the full-text screening criteria, two reviewers will independently review the full-texts of potentially relevant articles. Conflicts will be resolved through discussion, or by a third reviewer, and study selection will be reported using the PRISMA flow diagram.
A charting exercise will be completed to assess how outcomes are reported in the included reviews, and to refine which measurement tools and scales will be abstracted. The results of the charting exercise will be discussed with project stakeholders and clinical experts to help select the most relevant outcome measures for inclusion in the review.
Data will be abstracted based on review characteristics (e.g., year of conduct/literature search, number of

included studies, type of included study designs), patient characteristics (e.g., type and number of patients, age mean and standard deviation), interventions examined (e.g., type of intervention, dose/frequency) and outcomes examined (e.g., name of outcome, outcome measure/definition). A draft data abstraction form will be established after consultation with our methodologist and/or clinical experts. Prior to data abstraction, a calibration exercise of the data abstraction form will be completed on a random sample of five articles, and subsequently, all of the included studies will be abstracted by two reviewers, independently. A third reviewer will compile the files, will ensure that the data are correct, and will also resolve any conflicts.

Risk of bias (quality) assessment

The methodological quality of the included reviews will be appraised using the AMSTAR tool by two reviewers, independently.

Strategy for data synthesis

The results of the literature search and screening will be summarized descriptively. Abstracted data will be compiled in tables to enable an in depth comparison of the literature, and descriptive syntheses of interventions and outcomes. Conclusion statements reported in systematic reviews in which a meta-analysis has not been conducted will be categorized by two reviewers according to a pre-existing framework: positive (there is evidence of effectiveness), neutral (no evidence of effectiveness or no opinion), or negative (advised against use of the intervention or stated there is insufficient evidence). Contrasting insomnia interventions examined in the literature will help to determine the specific features required to improve care, as well as to identify gaps in the literature in order to target future research initiatives. Safety outcomes reported in primary research studies will be summarized using descriptive statistics (e.g., frequency, proportions), and a meta-analysis will not be conducted for these findings.

Analysis of subgroups or subsets

None planned.

Contact details for further information

Andrea Tricco
triccoa@smh.ca

Organisational affiliation of the review

Li Ka Shing Knowledge Institute of St. Michael's Hospital
knowledgetranslation.net

Review team members and their organisational affiliations

Dr Andrea Tricco. Knowledge Translation Program, Li Ka Shing Knowledge Institute, St. Michael’s Hospital; Division of Epidemiology, Dalla Lana School of Public Health, University of Toronto, Toronto, Canada
Ms Patricia Rios. Knowledge Translation Program, Li Ka Shing Knowledge Institute, St. Michael’s Hospital, Toronto, Ontario, Canada
Dr Roberta Cardoso. Knowledge Translation Program, Li Ka Shing Knowledge Institute, St. Michael’s Hospital, Toronto, Ontario, Canada
Ms Deanna Morra. Knowledge Translation Program, Li Ka Shing Knowledge Institute, St. Michael’s Hospital, Toronto, Ontario, Canada
Dr Zahra Goodarzi. Division of Geriatrics, Cumming School of Medicine, University of Calgary, Calgary, Alberta, Canada
Mr Bechara Farah. Canadian Agency for Drugs and Technology in Health (CADTH), Ottawa, Ontario, Canada
Ms Sharada Harricharan. Canadian Agency for Drugs and Technology in Health (CADTH), Ottawa, Ontario, Canada
Dr Sharon Straus. Knowledge Translation Program, Li Ka Shing Knowledge Institute, Department of Geriatric Medicine, University of Toronto, Toronto, Ontario, Canada

Collaborators

Mrs Becky Skidmore. Ottawa Hospital Research Institute, Ottawa, Ontario, Canada
Ms Alissa Epworth. Knowledge Translation Program, Li Ka Shing Knowledge Institute, St. Michael's Hospital, Toronto, Ontario, Canada
Dr Judith Leech. Respirology and Sleep Medicine, The Ottawa Hospital, Ottawa, Ontario, Canada
Dr Charles Morin. School of Psychology, Université Laval, Laval, Quebec, Canada
Dr Paul Salomon. General Medicine, Eastern Health Outpatients, St. John's, Newfoundland, Canada
Dr Louis de Léséleuc. Canadian Agency for Drugs and Technology in Health (CADTH), Ottawa, Ontario, Canada
Dr Mike Innes. Canadian Agency for Drugs and Technology in Health (CADTH), Ottawa, Ontario, Canada

Anticipated or actual start date

12 June 2017

Anticipated completion date

30 March 2018

Funding sources/sponsors

Canadian Institutes of Health Research (CIHR) Drug Safety and Effectiveness Network (DSEN); ACT is funded by a Tier 2 Canada Research Chair in Knowledge Synthesis and an Ontario Ministry of Research, Innovation, and Science Early Researcher Award; SES is funded by a Tier 1 Canada Research Chair in Knowledge Translation

Conflicts of interest

None known

Language

English

Country

Canada

Stage of review

Review_Ongoing

Subject index terms status

Subject indexing assigned by CRD

Subject index terms

Humans; Patient Safety; Safety; Sleep; Sleep Initiation and Maintenance Disorders; Treatment Outcome

Date of registration in PROSPERO

20 July 2017

Date of publication of this version

20 July 2017

Details of any existing review of the same topic by the same authors

Stage of review at time of this submission

NHS National Institute for Health Research

Stage	Started	Completed
Preliminary searches	Yes	Yes
Piloting of the study selection process	Yes	Yes
Formal screening of search results against eligibility criteria	Yes	No
Data extraction	No	No
Risk of bias (quality) assessment	No	No
Data analysis	No	No

Versions

20 July 2017

PROSPERO
This information has been provided by the named contact for this review. CRD has accepted this information in good faith and registered the review in PROSPERO. CRD bears no responsibility or liability for the content of this registration record, any associated files or external websites.
