# Supplementary material for: Comparative effectiveness and safety of pharmacological and non-pharmacological interventions for insomnia: an overview of reviews
Source: Syst Rev. 2019 Nov 15;8:281. doi: 10.1186/s13643-019-1163-9 (PMC6857325; doi:10.1186/s13643-019-1163-9)
Supplement: Supplementary file 2 — Additional file 2. The appendices include all supplemental data and information. Appendix A: PRIO-harms Checklist. Appendix B: Database Search Strategy and List of Gray Literature search sites. Appendix C: Review, participant, and intervention characteristics. Appendix D: AMSTAR Results. Appendix E: Detailed Tables of Results. Appendix F: Tables of primary studies by treatment comparison for outcomes with more than one included SR or SR + MA. [file 13643_2019_1163_MOESM2_ESM.pdf]

Additional File 2

Appendix A: PRIO-harms Checklist 3

Appendix B: Database Search Strategy and List of Grey Literature search sites 8

Appendix C: Review, participant, and intervention characteristics 11

Appendix D: AMSTAR Results 28

Appendix E: Detailed Tables of Results..35

Table E1. Benzodiazepines..35

Table E2. Non-benzodiazepines 36

Table E3. Suvorexant..40

Table E4. Antidepressants 43

Table E5. Anti-psychotics...50

Table E6. Melatonin...52

Table E7. Diphenhydramine 56

Table E8. Cognitive Behavioural Interventions...58

Table E9. Behavioural Interventions 92

Table E10. Mindfulness..96

Table E11. Combination Therapy...98

Appendix F: Tables of primary studies by treatment comparison for outcomes with more than one included SR or SR+MA 99

Table F1. Primary studies across included systematic reviews that examined Benzodiazepines 99

Table F2. Primary studies across included systematic reviews that examined non-benzodiazepines.................101

Table F3. Primary studies across included systematic reviews that examined Suvorexant 104

Table F4. Primary studies across included systematic reviews that examined antidepressants 104

Table F5. Primary studies across included systematic reviews that examined anti-psychotics 106

Table F6. Primary studies across included systematic reviews that examined melatonin 107

Table F7. Primary studies across included systematic reviews that compared diphenhydramine to inactive controls...109

Table F8. Primary studies across included systematic reviews that compared cognitive behavioural interventions to inactive controls 110

Table F9. Primary studies across systematic reviews that compared behavioural interventions to inactive controls 131

Table F10. Primary studies across included systematic reviews that examined mindfulness based interventions 133

Table F11. Primary studies across included systematic reviews that compared combination therapy to inactive controls....133

Appendix A: PRIO-harms Checklist

Section/Topic	(Sub-) item #	Checklist item	Reported on page #
**TITLE**			
1. Title	1a	Specify the study design with terms such as "overview of (systematic) reviews," "umbrella review," "(systematic) review of systematic reviews," or "(systematic) meta-review" in the title of the OoSRs.	1
	1b	Mention "safety" or harms related terms, or the adverse event(s) of interest in the title of the OoSRs.	1
**ABSTRACT**			
2. Structured-like summary	2a	Provide a structured-like abstract, as applicable: background, objective, data sources, selection criteria, data extraction, review appraisal, data synthesis methods, results, limitations, conclusions.	3-4
	2b	Report the main findings of analysis of harms undertaken in the OoSRs or/and in the included SRs.	3-4
**INTRODUCTION**			
3. Rationale	3a	Specify the rationale and the scope (wide or narrow agendas) for the overview in the context of an existing body of knowledge on the topic.	5-6
	3b	Provide a balanced presentation of potential benefits and harms of the intervention(s).	5-6
	3c[a]	Define which events are considered harms according to previous literature and provide a clear rationale for the specific harms included in the OoSRs.	N/A
4. Objectives (PICOS)	4	Provide an explicit statement of research question(s) that specifies PICOS:	6
		Participants [y] Interventions [y] Comparators [y] Outcomes [y] Study design [y]	
**METHODS**			
5. Protocol and registration	5a	Indicate clearly if a protocol exists or not.	6
	5b	If registered, provide the name of the registry (such as a valid Web address, PROSPERO).	6
6. Eligibility criteria & outcomes of	6a	Specify inclusion and exclusion criteria for study design, participants, interventions and comparators in detail.	7
	6b	List (and define whenever it is necessary) the outcomes for which data were recorded, ideally include prioritization of main and additional outcomes.	7

Section/Topic	(Sub-) item #	Checklist item	Reported on page #
interest	6c	Include adverse events as (primary or secondary) outcome of interest. Define them and grade their severity (such as mild, moderate, severe, fatal; severity could also be described in the appendix), if appropriate.	7-8
	6d[b]	Specify report characteristics (such as language restrictions, publication status, and years considered) used as criteria for eligibility for the OoSRs (see also item 7).	8
7. Information sources	7a	Search at least two electronic bases.	8
	7b	Search supplementary sources (e.g. hand-searching, reference lists, related reviews and guidelines, protocol registries, conference abstracts, and other gray literature).	8
	7c	Report the date last searched and/or dates of coverage for each database.	8
8. Search strategy [c]	8a	Specify full electronic search strategy (algorithm) for at least one database including any limits used (e.g. language and date restrictions-see also subitems 6d and 7c) such that it could be repeated.	Appendix B
	8b	Present any additional search process (e.g. algorithm or filter for adverse events, searches in pertinent websites) specifically to identify adverse events that have been investigated.	Appendix B
9. Data management & selection process	9a[d]	Describe the software that was used to manage records and data throughout the OoSRs.	9
	9b	Define what is a SR and provide the process for selecting SRs and its relevant details (screening the title and abstract or full text by at least two reviewers, selection by multiple independent investigators and resolving disagreements by consensus).	9
	9c	Report any attempt to handle overlapping (include one review among multiple potential candidates by choosing for example the most updated SR, the most methodologically rigorous SR or the SR with larger number of primary studies).	9
10. Additional search for primary studies	10	Report additional search to identify eligible primary studies (e.g. searching in more databases or update the search) and its relevant details.	N/A
11. Data collection process	11a	Describe the method of data extraction from included SRs (e.g. data collection form, extraction in duplicate and independently, resolving disagreements by consensus).	9
	11b	Report any processes for obtaining, confirming or updating data from investigators (e.g. contact with authors of included reviews, obtain data from primary studies of included reviews).	N/A

Section/Topic	(Sub-) item #	Checklist item	Reported on page #
12. Data items	12	List (and define whenever is necessary) the specific variables for which data were recorded (e.g. PICOS items, number of included studies and participants, dose, length of follow up, results, funding sources) and any data assumptions and simplifications made.	9
13. Assessment of methodological quality & quality of evidence	13a	State the evaluation of reporting or/and methodological quality (eg. using PRISMA or PRISMA-harms, AMSTAR or R-AMSTAR) of the included reviews.	10
	13b[e]	State the evaluation of quality for individual studies that were included in the SRs (inform whether tools such as Jadad or RoB of Cochrane were used by the included reviews) and for the additional primary studies.	N/A
	13c	State the evaluation of quality of evidence (e.g. using GRADE approach).	N/A
	13d	Describe the methods (e.g. piloted forms, independently, in duplicate) used for the quality assessment.	10
14. Meta-bias(es)	14	Specify any planned assessment of meta-bias(es) (such as publication bias or selective reporting across studies, ROBIS tool).	N/A
15. Data synthesis	15a	Specify clearly the method (narrative, meta-analysis or network meta-analysis) of handling or synthesizing data and their details (e.g. state the principal summary measures that were extracted or calculated, how heterogeneity was assessed, what statistical approaches were used if a quantitative synthesis has been conducted).	10
	15b	Describe the software that was used to analyze the data if a quantitative synthesis has been conducted.	N/A
	15c	Report if zero events are included in the studies and how they were handled in statistical analyses, if relevant.	N/A
	15d	Describe methods of any pre-specified additional analyses (such as sensitivity or subgroup analyses, meta-regression).	N/A
**RESULTS**			
16. Review & primary study selection	16a	Provide the details of review selection (e.g. numbers of reviews screened, retrieved, and included and excluded in the overview) and the number of the additional eligible primary studies that were included, ideally with a flow diagram of the overview process.	10
	16b	Present a flow diagram that gives separately the number of studies focused on harms outcomes.	Figure 1
	16c[c]	List the studies (full citation) that were excluded after reading the full text and provide reasons.	N/A
17. Review & primary study characteristics	17a[c]	Describe characteristics of each included SR in tables (such as title or author, search date, PICOS, design and number of studies included, number and age range of participants, dose/frequency, follow up period [treatment duration], review limitations, results or conclusion) and of each additional primary study.	10-11, Table 1-2, Appendix C
	17b	For each included SR report language and publication status restrictions that have been used.	N/A

Section/Topic	(Sub-) item #	Checklist item	Reported on page #
18. Overlapping	18	Present or/and discuss about overlapping of studies within SRs (at least one of the following):	12-17
		• Present measures of overlap (such as CCA).	N/A
		• Provide citation matrix.[c]	Appendix F
		• Give the number of index publications or/and discuss about overlapping.[f]	12-17
19. Present assessment of methodological quality & quality of evidence	19	Present results in text or/and tables[c] of any quality assessment (see also subitems 13a-c):	12, Figure 2, Appendix D
		• Reporting or/and methodological quality of the included SRs.	12, Figure 2, Appendix D
		• Inform for the quality of the individual studies that were included in the SRs (report results for sequence generation, allocation concealment, blinding, withdrawals, bias etc.) and for the additional included primary studies.	N/A
		• Quality of evidence.	N/A
20. Present meta-bias(es)	20	Present results of any assessment of meta-bias(es) (such as publication bias or selective reporting across studies, ROBIS assessment).	N/A
21. Synthesis of results	21a	Summarize and present the main findings of the overview for benefits and harms. If a quantitative synthesis has been conducted, present each summary measure with a confidence interval, prediction interval or a credible interval and measures of heterogeneity or inconsistency.	13-17, Table 3, Appendix E
	21b	Give results of any additional analyses (such as sensitivity, subgroup analyses, or meta-regression).	N/A
	21c	Report results for adverse events separately for each intervention.	13-17
**DISCUSSION**			
22. Summary of evidence	22	Provide a concise summary of the main findings with the strength and shortcomings of evidence for each main outcome.	18-19
23. Limitations	23a	Discuss limitations of either the overview or included studies (or both) (e.g. different eligibility criteria, limitations of searching reviews, language restrictions, publication and selection bias).	19-20

Section/Topic	(Sub-) item #	Checklist item	Reported on page #
	23b	Report possible limitations of the included reviews related to harms (issues of missing data and information, definitions of harms, rare adverse effects).	18-19
24. Conclusions	24a	Provide a general interpretation of the results in coherence with the review findings and present implications for practice; consider the harms equally as carefully as the benefits and in the context of other evidence.	20
	24b	Present implications for future research.	18-20
**AUTHORSHIP**			
25. Contributions of authors	25	Provide contributions of authors.	26
26. Dual (co-)authorship	26	Report about dual (co-)authorship in the limitation or declarations of interest section.	N/A
**FUNDING**			
27. Funding or other support	27a	Indicate sources of financial and other support for the OoSRs (direct funding) or for the authors (indirect funding), or report no funding.	26
	27b	Provide name for the overview funder and/or sponsor, or for the authors' supporters.	6, 26
	27c	Describe roles of funder(s), sponsor(s), and/or institution(s), if any, in conducted the OoSRs.	6, 26

Appendix B: Database Search Strategy and List of Grey Literature search sites

Multi-database search Strategy

1. exp "Sleep Initiation and Maintenance Disorders"/
2. (insomni* or hyposomni*).tw,kw.
3. (sleep* adj3 initiat* adj3 (disorder* or dysfunction* or problem*)).tw,kw.
4. (sleep* adj3 (mainten* or maintain*) adj3 (disorder* or dysfunction* or problem*)).tw,kw.
5. ((difficult* or disturb* or inabilit* or unable* or problem* or reduced) adj3 (asleep or sleep*)).tw,kw.
6. sleepless*.tw,kw.
7. (early adj1 (awake* or wake or wakes or waking)).tw,kw.
8. or/1-7 [INSOMNIA]
9. exp Child/ not (exp Adult/ and exp Child/)
10. exp Infant/ not (exp Adult/ and exp Infant/)
11. 8 not (9 or 10) [CHILD-ONLY REMOVED]
12. exp Animals/ not (exp Animals/ and Humans/)
13. 11 not 12 [ANIMAL-ONLY REMOVED]
14. (comment or editorial or interview or news).pt.
15. (letter not (letter and randomized controlled trial)).pt.
16. 13 not (14 or 15) [OPINION PIECES REMOVED]
17. limit 16 to systematic reviews
18. meta analysis.pt.
19. exp meta-analysis as topic/
20. (meta-analy* or metanaly* or metaanaly* or met analy* or integrative research or integrative review* or integrative review* or research integration or research review* or collaborative review*).tw,kw.
21. (systematic review* or systematic review* or evidence-based review* or evidence-based review* or (evidence adj3 (review* or review*)) or meta-review* or meta-review* or meta-synthes* or "review of reviews" or technology assessment* or HTA or HTAs).tw,kw.
22. exp Technology assessment, biomedical/
23. (cochrane or health technology assessment or evidence report).jw.
24. (network adj (MA or MAs)).tw,kw.
25. (NMA or NMAs).tw,kw.
26. indirect comparison?.tw,kw.
27. (indirect treatment* adj1 comparison?).tw,kw.
28. (mixed treatment* adj1 comparison?).tw,kw.
29. (multiple treatment* adj1 comparison?).tw,kw.
30. (multi-treatment* adj1 comparison?).tw,kw.
31. simultaneous comparison?.tw,kw.
32. mixed comparison?.tw,kw.
33. or/18-32
34. 16 and 33
35. 17 or 34 [SYSTEMATIC REVIEWS]

36. 35 use ppez [MEDLINE RECORDS]
37. exp insomnia/
38. (insomni* or hyposomni*).tw,kw.
39. (sleep* adj3 initiat* adj3 (disorder* or dysfunction* or problem*)).tw,kw.
40. (sleep* adj3 (mainten* or maintain*) adj3 (disorder* or dysfunction* or problem*)).tw,kw.
41. ((difficult* or disturb* or inabilit* or unable* or problem* or reduced) adj3 (asleep or sleep*)).tw,kw.
42. sleepless*.tw,kw.
43. (early adj1 (awake* or wake or wakes or waking)).tw,kw.
44. or/37-43 [INSOMNIA]
45. exp juvenile/ not (exp juvenile/ and exp adult/)
46. exp Child/ not (exp Adult/ and exp Child/)
47. exp Infant/ not (exp Adult/ and exp Infant/)
48. or/45-47
49. 44 not 48 [CHILD-ONLY REMOVED]
50. exp animal experimentation/ or exp models animal/ or exp animal experiment/ or nonhuman/ or exp vertebrate/
51. exp human/ or exp human experimentation/ or exp human experiment/
52. 50 not 51
53. 49 not 52 [ANIMAL-ONLY REMOVED]
54. editorial.pt.
55. letter.pt. not (letter.pt. and randomized controlled trial/)
56. 53 not (54 or 55) [OPINION PIECES REMOVED]
57. meta-analysis/
58. "systematic review"/
59. "meta analysis (topic)"/
60. (meta-analy* or metanaly* or metaanaly* or met analy* or integrative research or integrative review* or integrative review* or research integration or research review* or collaborative review*).tw,kw.
61. (systematic review* or systematic review* or evidence-based review* or evidence-based review* or (evidence adj3 (review* or review*)) or meta-review* or meta-review* or meta-synthes* or "review of reviews" or technology assessment* or HTA or HTAs).tw,kw.
62. biomedical technology assessment/
63. (cochrane or health technology assessment or evidence report).jw.
64. (network adj (MA or MAs)).tw,kw.
65. (NMA or NMAs).tw,kw.
66. indirect comparison?.tw,kw.
67. (indirect treatment* adj1 comparison?).tw,kw.
68. (mixed treatment* adj1 comparison?).tw,kw.
69. (multiple treatment* adj1 comparison?).tw,kw.
70. (multi-treatment* adj1 comparison?).tw,kw.
71. simultaneous comparison?.tw,kw.
72. mixed comparison?.tw,kw.
73. or/57-72

74. 56 and 73 [SYSTEMATIC REVIEWS]
75. 74 use emczd [EMBASE RECORDS]
76. Insomnia/
77. (insomni* or hyposomni*).tw,kw.
78. (sleep* adj3 initiat* adj3 (disorder* or dysfunction* or problem*)).tw,kw.
79. (sleep* adj3 (mainten* or maintain*) adj3 (disorder* or dysfunction* or problem*)).tw,kw.
80. ((difficult* or disturb* or inabilit* or unable* or problem* or reduced) adj3 (asleep or sleep*)).tw,kw.
81. sleepless*.tw,kw.
82. (early adj1 (awake* or wake or wakes or waking)).tw,kw.
83. or/76-82 [INSOMNIA]
84. Meta Analysis/
85. (meta-analy* or metanaly* or metaanaly* or met analy* or integrative research or integrative review* or integrative review* or research integration or research review* or collaborative review*).tw,kw.
86. (systematic review* or systematic review* or evidence-based review* or evidence-based review* or (evidence adj3 (review* or review*)) or meta-review* or meta-review* or meta-synthes* or "review of reviews" or technology assessment* or HTA or HTAs).tw,kw.
87. (network adj (MA or MAs)).tw,kw.
88. (NMA or NMAs).tw,kw.
89. indirect comparison?.tw,kw.
90. (indirect treatment* adj1 comparison?).tw,kw.
91. (mixed treatment* adj1 comparison?).tw,kw.
92. (multiple treatment* adj1 comparison?).tw,kw.
93. (multi-treatment* adj1 comparison?).tw,kw.
94. simultaneous comparison?.tw,kw.
95. mixed comparison?.tw,kw.
96. or/84-95
97. 83 and 96 [SYSTEMATIC REVIEWS]
98. 97 use ppez
99. 97 use emczd
100. 97 not (98 or 99) [PSYCINFO RECORDS]
101. 36 or 75 or 100 [ALL DATABASES]

List of Grey Literature search sites

- Health Technology Assessment Sites
    - Canadian Agency for Drugs and Technologies in Health (www.cadth.ca)
    - Agency for Healthcare Research and Quality (www.ahrq.gov)
    - The International Network of Agencies for Health Technology Assessment (www.inahta.org)
    - National Institute for Health and Care Excellence (www.nice.org.uk)
- International prospective register of systematic reviews (PROSPERO: https://www.crd.york.ac.uk/prospero/)

Appendix C: Review, participant, and intervention characteristics

Author, Year<br><br>Country	# and type of included studies<br><br>Sample size<br><br>Literature search dates	Age and Sex<br><br>[mean (SD)]; % female<br><br>Co-morbidities	Treatment comparison (doses [mg])<br><br>Delivery method and setting	Frequency;<br><br>duration of treatment; length of follow-up (range, weeks)
		Systematic Reviews with Meta-analysis (n=35)		
Ballesio, 2017<br>Germany	47 RCTs<br><br>4,317<br>Intervention: 2,448<br>Control: 1,869<br><br>1986-2014	51.9 (NR); 62.8%<br><br>None	CBT-I group v control<br><br>CBT-I individual v control<br><br>CBT-I self-help v control<br><br>Group behavioural therapy v control<br><br>Control conditions: sleep hygiene, wait-list control, placebo	NR; NR; NR
Brasure, 2015<br><br>USA	46 RCTs<br><br>5764<br><br>2004-2015	NR<br>Pain, chronic low back pain, hearing impairment	Zolpidem (10 or 15mg) v placebo<br>outpatient	"as needed"; NR; 4 to 34.76
			Doxepin (1, 3, or 6 mg) v placebo<br>outpatient	NR; NR; 4 to 12
			Suvorexant (15 or 20mg) v placebo	NR; NR; 4
			CBT-I v control (placebo/sham treatment, wait-list, no treatment, or sleep hygiene/sleep education)<br><br>Individual or group (in-person), Phone, Self-help (books, handouts, or electronic resources); outpatient	Once a week; ≤1 hour; 4 to 104

Author, Year<br>Country	# and type of included studies<br>Sample size<br>Literature search dates	Age and Sex<br>[mean (SD)]; % female<br>Co-morbidities	Treatment comparison (doses [mg])<br>Delivery method and setting	Frequency;<br>duration of treatment; length of follow-up (range, weeks)
			Sleep restriction v control<br>Relaxation therapy v control<br>Behavioural intervention or brief behavioral therapy v control<br>Outpatient<br>Control conditions: sham treatment/placebo, wait-list control, no treatment, or sleep hygiene/sleep education	NR; NR; 4 to 26<br>NR; NR; 4<br>NR; NR; 4
Buscemi, 2004<br>Canada	139<br>NR<br>NR-2003	NR<br>NR	Melatonin (0.1, 0.3, 0.5, 1, 2, 3, 5, or 6 mg) v placebo<br>Oral - sustained-release, fast-release, or immediate-release	Nightly; 16 days; NR
Buscemi, 2005<br>Canada	97 RCTs<br>NR<br>NR-2004	NR<br>NR	Flurazepam (15 or 30 mg) v placebo<br>Temazepam (15, 20, or 30 mg) v placebo<br>Triazolam (0.125, 0.25, or 0.5 mg) v placebo	NR; NR; NR
			Zolpidem (5, 10, 15, or 20 mg) v placebo<br>Zopiclone (7.5 mg) v placebo	NR; NR; NR
			Melatonin (0.1, 0.3, 0.5, 1, 2, 3, or 5 mg) v placebo	NR; NR; NR
			Doxepin (25 mg or 25 -50 mg) v placebo<br>Trazodone (50 mg; 150 -250 mg) v placebo	NR; NR; NR
			CBT v placebo	NR; NR; NR

**Author, Year**<br>**Country**	**# and type of included studies**<br>**Sample size**<br>**Literature search dates**	**Age and Sex**<br>**[mean (SD)]; % female**<br>**Co-morbidities**	**Treatment comparison (doses [mg])**<br>**Delivery method and setting**	**Frequency;**<br>**duration of treatment; length of follow-up (range, weeks)**
			CBT + relaxation v placebo<br>CBT + relaxation v relaxation<br>CBT + relaxation v CBT<br>Relaxation v placebo<br>Triazolam (0.25 mg) or Temazepam (7.5 -30mg) + CBT v placebo<br>CBT components: cognitive control, stimulus control, cognitive distraction, cognitive therapy, sleep restriction/compression, sleep hygiene, paradoxical intention<br>Relaxation components: relaxation training, group relaxation, progressive muscle relaxation, Autogenic training, breathing process training, EMG biofeedback, hypnotic relaxation	
Cheng, 2012<br>Hong Kong	6 RCTs<br>431<br>1990-2011	NR<br>Cancer-related insomnia	CBT-I (sleep hygiene, stimulus control, relaxation training, sleep restriction, cognitive restructuring, relapse prevention, psychoeducation) v control (wait-list control, sleep-monitoring)<br>Computer or mobile phone; Home-based	NR; 5 to 9; 3 to 26
Ferracioli-Oda, 2013<br>USA	19 RCTs<br>1683<br>NR	NR<br>Delayed sleep phase disorder, REM sleep behaviour disorder	Melatonin (0.1, 0.3, 0.5, 1, 2, 3, or 5 mg) v placebo	NR; NR; 1 to 26

Author, Year<br>Country	# and type of included studies<br>Sample size<br>Literature search dates	Age and Sex<br>[mean (SD)]; % female<br>Co-morbidities	Treatment comparison (doses [mg])<br>Delivery method and setting	Frequency;<br>duration of treatment; length of follow-up (range, weeks)
Gong, 2016<br>China	6 RCTs<br>330<br>Inception-2015	NR<br>Depression, cancer	Mindfulness based stress reduction, mindfulness meditation, or mindfulness based therapy for insomnia v control (wait-list control, sleep hygiene education, self-monitoring)	NR; NR; 6 -8
Ho, 2015<br>Hong Kong	20 RCTs<br>2411<br>Inception-2013	49.3 (NR); 74.2%<br>None	CBT (stimulus control, sleep restriction, sleep hygiene, relaxation, cognitive therapy) v control (wait-list control, routine care, or no treatment)<br>Self-help; home	NR; NR; 4 to 52
Ho, 2016<br>Hong Kong	11 RCTs<br>Intervention: 303<br>Control: 290<br>Inception-2014	45.3 (NR); 35.6%<br>PTSD, depression	CBT (image rehearsal therapy, exposure, rescripting and relaxation therapy, mind-body bridging, behavioural sleep intervention) v control (wait list control, sleep hygiene, or placebo)<br>Individual or group delivery	NR; 2 -12; 1 to 26
Hwang, 2016<br>South Korea	37<br>13 RCTs<br>24 Quasi-experimental<br>2150<br>NR	NR<br>NR	Behavioural therapy v control (unspecified)	NR; NR; NR
Irwin, 2006<br>United States	23 RCTs<br>NR<br>1966-2004	NR<br>None	CBT (relaxation/biofeedback/hypnosis, sleep compression/restriction, paradoxical intention) v control (unspecified)	NR; NR; NR
Johnson, 2016	8 RCTs	NR	CBT-I v control (wait-list control, treatment as usual, sleep	NR; NR;13 to 52

Author, Year<br><br>Country	# and type of included studies<br><br>Sample size<br><br>Literature search dates	Age and Sex<br><br>[mean (SD)]; % female<br><br>Co-morbidities	Treatment comparison (doses [mg])<br><br>Delivery method and setting	Frequency;<br><br>duration of treatment; length of follow-up (range, weeks)
Canada	752<br>Intervention: 434<br>Control: 318<br><br>Inception-2014	Cancer	education, behavioural placebo, mindfulness-based stress reduction)<br><br>Individual, group, video or online-based	
Kishi, 2015<br><br>Japan	4 RCTs<br><br>3076<br><br>NR-2015	56.6 (NR); 61.8%<br><br>None	Suvorexant (10 -80mg/day) v placebo<br><br>Outpatient	NR; NR; 4 to 52
Koffel, 2015<br><br>USA	8 RCTs<br><br>NR<br><br>NR-2013	NR<br><br>Chronic pain, cancer, fibromyalgia, chronic illness, arthritis	CBT-I (stimulus control, sleep restriction, addressing dysfunctional beliefs about sleep) v control (wait-list, treatment as usual, placebo)<br><br>Group delivery; community-based	>1 session; NR; 13.04 to 52
Kuriyama, 2017<br><br>Japan	4 RCTs<br><br>3076<br><br>NR-2016	56.3 (15.3); 61.5%<br><br>None	Suvorexant (10, 15, 20, 30, 40, or 80mg/d) v placebo	NR; NR; 4 to 52
Lee, NA [unpublished]<br><br>South Korea	18 RCTs<br><br>NR<br><br>NR-2014	NR<br><br>None	Melatonin (0.1, 0.3, 1, 2, 5, 12, or 75 mg; 0.05, 0.1, or 0.15 mg/kg) v placebo<br>Transbuccal; sustained release, fast release, controlled release, or prolonged release	NR; 4 days to 26 weeks; NR
Liu, 2017<br><br>China	7 RCTs<br><br>Intervention: 743	NR<br><br>None	Doxepin (1, 3, or 6 mg) v placebo	NR; NR; NR

Author, Year<br>Country	# and type of included studies<br>Sample size<br>Literature search dates	Age and Sex<br>[mean (SD)]; % female<br>Co-morbidities	Treatment comparison (doses [mg])<br>Delivery method and setting	Frequency;<br>duration of treatment; length of follow-up (range, weeks)
	Control: 733<br>NR-2016			
McCleery, 2016<br>UK	3 RCTs<br>222<br>NR-2016	NR<br>Dementia	Melatonin (5 or 1 mg immediate-release, 2or 2.5 mg slow-release) v placebo<br>Tablet; long-term care and community care	Nightly (1-2 hours before bedtime); NR; 8 to 28
Montgomery, 2003<br>USA	6 RCTs<br>282<br>224 in MA<br>Inception -2002	NR<br>Parasomnia, sleep apnea	CBT (sleep hygiene, stimulus control, muscle relaxation, sleep restriction, cognitive therapy, education, imagery training) v control (wait-list control, placebo)<br>Group or individual delivery	NR; NR; 13 to 104
Navarro-Bravo, 2015<br>Spain	9 RCTs<br>699<br>Intervention: 352<br>Control: 347<br>NR-2012	NR<br>Cancer survivor, depression, restless leg syndrome, osteoarthritis	CBT (sleep restriction, stimulus control, sleep education/hygiene) v control (placebo, wait-list control, stress management and wellness training, treatment as usual, sleep hygiene/education)	NR; NR; 5 to 8
Okajima, 2011<br>Japan	14 RCTs<br>Intervention: 454<br>Control: 384<br>1990-2009	NR<br>None	CBT (sleep hygiene education, sleep restriction, stimulus control, cognitive therapy, relaxation, paradoxical intention) v control (placebo, wait-list control, treatment as usual, sleep hygiene education)<br>Individual or group delivery	1 – 8 sessions; NR; 4 to 104
Sateia, 2017<br>USA	129 RCTs<br>46 in MA	NR<br>None	Temazepam (15 mg) v placebo	NR; 5 days to 8 weeks; NR
			Zolpidem (10 mg) v placebo	NR; 2 to 32; NR
			Melatonin (2mg) v placebo	Nightly; 3; NR

Author, Year<br><br>Country	# and type of included studies<br><br>Sample size<br><br>Literature search dates	Age and Sex<br><br>[mean (SD)]; % female<br><br>Co-morbidities	Treatment comparison (doses [mg])<br><br>Delivery method and setting	Frequency;<br><br>duration of treatment; length of follow-up (range, weeks)
	NR<br><br>NR-2016		Doxepin (3 mg) v placebo<br>Doxepin (6 mg) v placebo	NR; 2 nights to 12 weeks; NR<br>NR; 2 nights to 5 weeks; NR
			Diphenhydramine (50 mg) v placebo	Nightly; 2; NR
Seda, 2015<br><br>USA	8 RCTs<br>NR<br><br>NR	NR<br><br>PTSD, nightmares	Imagery rehearsal therapy v CBT (stimulus control, sleep restriction therapy) + Imagery rehearsal therapy<br><br>Face-to-face, group, self-help; outpatient	1 to 8 sessions; NR; NR
Seyffert, 2016<br><br>USA	15 RCTs<br><br>2392<br><br>NR-2015	NR<br><br>Major depression	CBT (sleep education, stimulus control, sleep restriction, relaxation, sleep hygiene, cognitive techniques) v control (wait-list control, internet control, treatment as usual)<br><br>Internet or at home delivery	Weekly; NR; 4 to 48
Soldatos, 1999<br><br>Greece	75 (designs NR)<br><br>1276<br><br>1966-1997	NR<br><br>None	Triazolam (0.25 or 0.5 mg) v placebo	NR; 1 to 42 nights; NR
			Zopiclone (7.5, 10, or 15 mg) v placebo<br>Zolpidem (10, 15, or 20mg) v placebo	NR; 5 to 113 nights; NR<br>NR; 1 to 35 nights; NR
Tang, 2015<br><br>UK	11 RCTs<br><br>1066<br>965 in MA<br><br>Inception-2014	45 -61 (NR); 55 - 100%<br><br>Chronic pain, cancer, back pain, arthritis	CBT-I (psychoeducation, sleep hygiene, stimulus control, sleep restriction, cognitive therapy, relaxation) v control (waitlist, treatment as usual, sleep hygiene advice, healthy eating/nutrition advice)<br><br>Face-to-face, phone, internet, group or individual delivery	3 to 7 sessions averaging 69 to 120 minutes; 60 days; 13 to 52
Trauer, 2015<br><br>Australia	20 RCTs<br><br>1162<br><br>Inception-2015	55.6 (NR); 64.3%<br><br>None	CBT (cognitive therapy, stimulus control, sleep restriction, relaxation, sleep hygiene) v control (wait-list, treatment as usual, sleep hygiene, sham, placebo)<br><br>Group or individual delivery with aids such as telephone,	NR; NR; 4 to 48

**Author, Year**<br><br>**Country**	**# and type of included studies**<br><br>**Sample size**<br><br>**Literature search dates**	**Age and Sex**<br><br>**[mean (SD)]; % female**<br><br>**Co-morbidities**	**Treatment comparison (doses [mg])**<br><br>**Delivery method and setting**	**Frequency;**<br><br>**duration of treatment; length of follow-up (range, weeks)**
			audiocassettes, or written material	
van Straten, 2009<br><br>Netherlands	10 RCTs<br>9 in MA<br><br>1000<br>Intervention: 580<br>Control: 420<br><br>NR-2007	NR<br><br>Alcohol dependence, chronic disease	CBT (self-help, stimulus control, sleep restriction, cognitive therapy, sleep hygiene, relaxation, in-bed exercises) v wait-list control<br><br>Audiotape, videotape, written materials; internet + face-to-face, telephone, or e-mail support; home delivery (self-help)	Weekly; NR; 17 to 43.5
van Straten, 2007<br><br>Netherlands	87 RCTs<br><br>6303<br>Intervention: 3724<br>Control: 2579<br><br>NR-2015	NR<br><br>chronic pain; cancer; alcohol dependence; hearing problems; post-traumatic stress disorder; chronic obstructive pulmonary disease	CBT (relaxation, sleep restriction, stimulus control, paradoxical intention, identifying and challenging dysfunctional thought) v control (wait-list, no treatment, psycho-education, placebo)<br><br>Group, individual, phone, or self-help delivery	NR; 2 to 16 sessions; NR
Xu, 2015<br><br>China	6 RCTs<br><br>484<br><br>NR-2013	NR<br><br>Dementia	Melatonin (1.5, 2.5, 2.9, 3, 5, 6, 8.5, or 10mg) v placebo sustained- or immediate- release	NR; NR; 1.43 to 10
Yang, 2014<br><br>China	3 RCTs<br><br>184	NR<br><br>Dialysis-dependent patients with end stage renal disease	CBT + relaxation v control (sleep hygiene, treatment as usual)<br><br>Relaxation training delivered via CD	Daily (relaxation), 3 to 4 sessions weekly (CBT); NR; 4 to 8

Author, Year<br><br>Country	# and type of included studies<br><br>Sample size<br><br>Literature search dates	Age and Sex<br><br>[mean (SD)]; % female<br><br>Co-morbidities	Treatment comparison (doses [mg])<br><br>Delivery method and setting	Frequency;<br><br>duration of treatment; length of follow-up (range, weeks)
Ye, 2016<br><br>China	14 RCTs<br><br>1604<br>Intervention: 1013<br>Control: 591<br><br>NR-2106	NR<br><br>Cancer, depression	CBT (sleep hygiene education, cognitive restructuring, stimulus control, sleep restriction, relaxation therapy, hierarchy development, imagery training, scheduled pseudo desensitization, breathing control) v control (wait-list, treatment as usual, internet/email/telephone based control)<br><br>CBT delivered over the internet	NR; NR; 4 to 52
Yuan, 2010<br><br>China	4 RCTs<br><br>Intervention: 171<br>Control: 169<br><br>NR-2009	NR<br><br>None	Doxepin (1 mg) v placebo<br>Doxepin (3 mg) v placebo<br>Doxepin (6 mg) v placebo<br>Doxepin (25 mg) v placebo	NR; NR; NR<br>NR; NR; NR<br>NR; NR; NR<br>NR; NR; NR
Zachariae, 2016<br><br>Denmark	11 RCTs<br><br>1460<br>Intervention: 790<br>Control: 670<br><br>1991-2015	NR<br><br>Cancer	CBT (stimulus control, sleep hygiene, cognitive therapy, sleep restriction, relaxation technique) v control (wait-list control, treatment as usual, active control)<br><br>Internet/home delivery	NR; NR; 4 to 48
Zhang, 2016<br><br>China	9 RCTs<br><br>NR<br><br>NR-2015	NR<br><br>Alzheimer's disease, Parkinson's disease, REM sleep behavior disorder	Melatonin (2, 2.5, 3, 5, 6, 8.5, or 50 mg/d) v placebo	NR; 2 to 24; NR
		Systematic reviews without meta-analysis (n=29)		
Anderson, 2014<br><br>USA	12<br>4 RCTs<br>8 design unspecified	NR<br><br>depression/major	Quetiapine (25mg; increased to 50 or 75 mg) – pre-/post-intervention comparison	Daily; 2 to 12 weeks; NR

Author, Year<br><br>Country	# and type of included studies<br><br>Sample size<br><br>Literature search dates	Age and Sex<br><br>[mean (SD)]; % female<br><br>Co-morbidities	Treatment comparison (doses [mg])<br><br>Delivery method and setting	Frequency;<br><br>duration of treatment; length of follow-up (range, weeks)
	NR	depressive disorder, bipolar disorder, breast cancer, Parkinson's disease, schizophrenia, polysubstance abuse (withdrawal)	Oral; inpatient<br><br>Quetiapine (25 -100mg; 340 mg) vs Placebo<br><br>Oral	
Bellon, 2006<br><br>USA	15 RCTs<br><br>452	NR<br><br>Schizophrenia, dementia, medically ill patients, Alzheimer's disease	Melatonin (0.3, 0.5, 1, 2, 2.5, 5, 6, 10, or 75 mg)<br>Fast sustained release	Nightly/bedtime (4 hrs. after bedtime); 1 to 4weeks; NR
Bogdanov, 2017<br><br>Australia	4<br>1 RCT<br>3 Quasi-experimental<br><br>NR	Mean range 27 – 54<br><br>Traumatic brain injury	CBT v control (unspecified)<br><br>Problem solving therapy v sleep education<br><br>Phone call	NR; 60 minutes; 4 to 13<br><br>Fortnightly; NR; NR
Brooks, 2014<br><br>USA	4<br>3 RCTs<br>1 Quasi-experimental<br><br>NR	NR<br><br>Alcohol use	CBT-I vs control (unspecified)<br><br>Individual, in-person<br><br>Progressive relaxation training v control (unspecified)<br><br>Individual, delivered by psychologist	5 to 9 sessions; NR; 26<br><br>10 sessions; NR; NR
Chase, 1997	5 RCTs	NR	Melatonin (1, 2, 5, or 75 mg)	NR; NR; NR

Author, Year<br><br>Country	# and type of included studies<br><br>Sample size<br><br>Literature search dates	Age and Sex<br><br>[mean (SD)]; % female<br><br>Co-morbidities	Treatment comparison (doses [mg])<br><br>Delivery method and setting	Frequency;<br><br>duration of treatment; length of follow-up (range, weeks)
USA	66	Patients with at least one chronic disease	Controlled sustained release	
Chiesa, 2009<br><br>Italy	3 Observational<br><br>63	NR<br><br>NR	Pharmacotherapy (unspecified) + Mindfulness (unspecified)	NR; NR; NR
Cimolai, 2007<br><br>Canada	8<br>2 RCTs<br>6 NRCTs<br><br>474	NR<br><br>NR	Zopiclone (7.5 mg) – pre-/post- intervention comparison<br>Zopiclone (7.5 mg) vs Triazolam<br>Zopiclone (7.5 mg) vs Zolpidem<br>Zopiclone (7.5 mg) vs Flurazepam<br>Zopiclone (7.5 mg) vs. Temazepam	NR; NR; NR
Citrome, 2014<br><br>USA	4 RCTs<br><br>Intervention: 1279<br>Control: 1274	NR<br><br>None	Suvorexant (15 or 20 mg) v placebo<br><br>Suvorexant (20 or 40 mg) v placebo<br><br>Oral	NR; NR; 12
Coe, 2012<br><br>USA	2<br>1 RCT<br>1 NRCT<br><br>34	NR<br><br>None	Quetiapine (25 mg titrated up to 75mg) – pre-/post-intervention comparison<br><br>Quetiapine (25 mg) vs Placebo	NR; NR; 2 to 6
Costello, 2014<br><br>USA	4 RCTs<br><br>845	NR<br><br>None	Melatonin (0.3, 1, 2, or 5 mg) v placebo<br>Oral; sustained release	Daily; NR; NR
Culpepper, 2015<br><br>USA	11 RCTs<br><br>1590	NR<br><br>None	Melatonin (0.3, 1, 2, or 5 mg) v placebo<br>Fast release capsule; sustained-release synthetic tablet; prolonged-release tablet	Daily; NR; 1 to 29
			Diphenhydramine (50 mg) v placebo<br><br>Tablet	Daily; NR; 1 day to 4 weeks

Author, Year<br><br>Country	# and type of included studies<br><br>Sample size<br><br>Literature search dates	Age and Sex<br><br>[mean (SD)]; % female<br><br>Co-morbidities	Treatment comparison (doses [mg])<br><br>Delivery method and setting	Frequency;<br><br>duration of treatment; length of follow-up (range, weeks)
Dickerson, 2014<br><br>USA	7 RCTs<br><br>352<br>Intervention: 220<br>Control:132	NR<br><br>Cancer	CBT – pre-/post- intervention comparison<br><br>CBT vs control (usual treatment, waitlist crossover, waitlist control)<br><br>EEG biofeedback v wait-list control	Weekly; NR; 4 to 8<br><br>NR; NR; NR
Hellstrom, 2011<br><br>Sweden	3 RCTs<br><br>209<br>Intervention: 103<br>Control: 106	NR<br><br>NR	Mental imagery v usual care<br><br>In-person; inpatient	Daily; NR; NR
Howell, 2014<br><br>Canada	7 RCTs<br><br>NR	NR<br><br>Cancer	CBT (sleep hygiene, sleep restriction, stimulus control, cognitive restructuring, relaxation therapies) v control (usual care, wait-list control, health eating and nutrition, sleep education and hygiene, no treatment)<br><br>Individual delivery	Weekly; NR; 8 to 74
Ishak, 2012<br><br>USA	7<br><br>NR	NR<br><br>Breast, prostate gynecological, bowel cancer	Zopiclone (NR) vs Placebo<br>Zolpidem (10 mg) vs Zolpidem (10 mg)	NR; NR; NR<br>5 nights/week + placebo 2 nights/week or once-daily; NR; 2

**Author, Year**<br><br>**Country**	**# and type of included studies**<br><br>**Sample size**<br><br>**Literature search dates**	**Age and Sex**<br><br>**[mean (SD)]; % female**<br><br>**Co-morbidities**	**Treatment comparison (doses [mg])**<br><br>**Delivery method and setting**	**Frequency;**<br><br>**duration of treatment; length of follow-up (range, weeks)**
			CBT vs control (placebo, no treatment, unspecified, usual care)<br><br>CBT group vs CBT individual:<br><br>CBT components: psychoeducation, sleep hygiene, stimulus control, sleep restriction, relaxation exercises, cognitive restructuring	NR; NR; 14 to 52<br><br>Weekly; NR; 4 to 24
Kolla, 2011<br><br>USA	4<br>2 RCTs<br>2 NRCTs<br><br>240	NR<br><br>Alcohol recovery	Triazolam (0.5 -1 mg) – pre-/post- intervention comparison<br>Oral	NR; NR; 4
			Trazodone (50 -200 mg) v placebo<br>Oral	NR; NR; 4 to 24
			Quetiapine (300 -800mg) – pre-/post- intervention comparison<br><br>Oral	NR; NR; 16
Mayers, 2005<br><br>UK	6 RCTs<br><br>466	NR<br><br>Depression, dysthymia	Zolpidem (10 mg) v placebo<br>Oral	NR; NR; NR
			Trazodone (50, 75, or 100 mg) v placebo<br>Doxepin (25 – 50 mg) vs placebo<br>Trazodone (50 mg) vs Zolpidem (10 mg)<br>Oral	NR; NR; NR<br>NR; NR; NR<br>NR; 2; NR

Author, Year<br><br>Country	# and type of included studies<br><br>Sample size<br><br>Literature search dates	Age and Sex<br><br>[mean (SD)]; % female<br><br>Co-morbidities	Treatment comparison (doses [mg])<br><br>Delivery method and setting	Frequency;<br><br>duration of treatment; length of follow-up (range, weeks)
McCurry, 2007<br><br>USA	11 (designs unspecified)<br><br>NR	NR<br><br>Medical Illness	CBT vs control (delayed treatment, wait-list control, placebo, stress management)<br><br>Individual delivery<br><br>CBT vs CBT + temazepam<br><br>Group delivery<br><br>CBT components: sleep hygiene/education, relaxation, sleep restriction/compression, stimulus control	Weekly; NR; NR<br><br>Weekly; NR; NR
			Sleep restriction therapy, nap sleep restriction therapy, sleep compression, or sleep compression guidance v control (sleep hygiene, placebo, wait-list control)<br><br>Individual delivery	Weekly; NR; NR
Mendelson, 2005<br><br>USA	18 (designs unspecified)<br><br>1667<br>Intervention: 1195<br>Control: 472	NR<br><br>Depression	Zolpidem (10 mg) v placebo	Nightly; NR; NR
			Trazodone (50 to 600 mg) – pre-/post- intervention comparison<br>Trazodone (50 to 300 mg) vs placebo<br>Trazodone (50 mg) vs Zolpidem (10 mg)	NR; NR; NR<br>Nightly; NR; NR<br>Nightly; NR; NR
Miller, 2014	9<br>4 RCTs	53.3 (10.2); NR	Sleep restriction therapy v control (relaxation therapy,	NR; NR; 13 to 52

Author, Year<br><br>Country	# and type of included studies<br><br>Sample size<br><br>Literature search dates	Age and Sex<br><br>[mean (SD)]; % female<br><br>Co-morbidities	Treatment comparison (doses [mg])<br><br>Delivery method and setting	Frequency;<br><br>duration of treatment; length of follow-up (range, weeks)
UK	1 NRCT<br>4 Observational<br><br>380	None	wait-list control, sleep hygiene instructions)	
Swainston Harrison, 2005<br><br>New Zealand	3 RCTs<br><br>145<br>Intervention: 102<br>Control: 43	NR<br><br>None	Triazolam (0.5 mg) v placebo<br>Tablet; bedtime	NR; NR; 4
			Zolpidem (10 mg) vs Placebo<br>Zolpidem (10 mg) vs Triazolam<br>Tablet; bedtime	NR; NR; 7<br>NR; NR; 2 to 7
Tamrat, 2013<br><br>USA	8<br>4 RCTs<br>2 NRCTs<br>2 Observational<br><br>508	NR<br><br>Cancer, post-coronary artery bypass grafting, psychiatric disease [all hospitalized patients]	Relaxation techniques, audiotape guided imagery, or relaxation tapes v control (usual care, solitary activity, baseline)<br><br>Inpatient setting	Nightly; NR; NR
Taylor, 2014<br><br>USA	16 RCTs<br><br>571	NR<br><br>Depression, post-traumatic stress disorder, alcohol dependence, hypnotic	CBT (stimulus control, sleep restriction, relaxation therapy, cognitive therapy, image rehearsal therapy, medication withdrawal) v control (wait-list, usual care, sleep hygiene, hypnotic/medication withdrawal)<br><br>In-person delivery	NR; 2 -10 sessions; NR
Vande Griend, 2012<br><br>USA	16 RCTs<br><br>NR	NR<br><br>Alcohol detoxification,	Trazodone (50 mg) vs Placebo<br>Doxepin (1, 3, 6, 25, or 50 mg) vs placebo<br>Trazodone (50 mg) vs Zolpidem (NR)	NR; 1 to 4 weeks; NR<br>NR; 1 night to 12 weeks ;NR<br>NR; 2; NR

Author, Year<br><br>Country	# and type of included studies<br><br>Sample size<br><br>Literature search dates	Age and Sex<br><br>[mean (SD)]; % female<br><br>Co-morbidities	Treatment comparison (doses [mg])<br><br>Delivery method and setting	Frequency;<br><br>duration of treatment; length of follow-up (range, weeks)
		methadone-maintained	Diphenhydramine (50 mg) v placebo	NR; 5 to 28 days; NR
Venables, 2014<br><br>UK	22<br>16 RCTs<br>5 Quasi-experimental<br>1 Observational<br><br>1794	NR<br><br>adult cancer patients undergoing curative treatment	Self-help CBT – pre-/post- intervention comparison<br><br>Video; home<br><br>Professionally administered CBT – pre-/post- intervention comparison<br><br>Group CBT – pre-/post- intervention comparison	NR; NR; NR<br><br>NR; NR; NR<br><br>NR; NR; NR
Vural, 2014<br><br>Netherlands	5<br>4 RCTs<br>1 NRCTs<br><br>207	NR<br><br>NR	Melatonin (0.4, 0.5, 2, or 4 mg) v control (unspecified)<br>sustained release transbuccal patch or immediate release	Nightly (bedtime); 4 to 42 days; 1 to 24
Wang, 2005<br><br>Taiwan	6 RCTs<br><br>255	NR<br><br>NR	CBT (stimulus control, sleep restriction, cognitive therapy, sleep hygiene education, sleep scheduling) vs control (quasi desensitization, self-monitoring, sleep hygiene, wait list)<br><br>CBT (stimulus control, sleep restriction, sleep hygiene education) vs relaxation	NR; NR; 12 to 104<br><br>NR; NR; 24
			Behavioural therapy (stimulus control, relaxation therapy, sleep education) v stimulus control	NR: NR; 4
Wine, 2009	3 NRCTs	NR	Quetiapine (25 -75, or 12.5 -50mg) – pre-/post-	Bedtime, as needed; NR; 6

**Author, Year**<br><br>**Country**	**# and type of included studies**<br><br>**Sample size**<br><br>**Literature search dates**	**Age and Sex**<br><br>**[mean (SD)]; % female**<br><br>**Co-morbidities**	**Treatment comparison (doses [mg])**<br><br>**Delivery method and setting**	**Frequency;**<br><br>**duration of treatment; length of follow-up (range, weeks)**
USA	Total:50	Posttraumatic stress disorder,<br>Parkinson's disease	intervention comparison<br><br>Quetiapine (25 -300mg) v untreated control<br><br>Tablet	to 12<br><br>Bedtime, as needed; NR; 6
Yeung, 2015<br><br>Hong Kong	8 RCTs<br><br>Total:1513	NR<br><br>Anxiety, depression	Doxepin (3, 6, or 25-300 mg) v placebo<br>Oral	NR; NR; 4 to 12

Appendix D: AMSTAR Results

**Author, Year**	1. PICO components	2. A priori design	3. Rationale for study selection	4. Literature search	5. Duplicate Selection	6. Duplicate Abstraction	7. List of excluded studies	8. Description of included studies	9a. RoB in RCTs	9b. RoB in non-randomized studies	10. Funding sources	11. Appropriate MA methods	12. Used RoB in MA	13. Used RoB in interpreting results	14. Discussion of heterogeneity	15. Publication bias	16. Conflict of Interest	**Overall Rating**
Systematic Reviews with meta-analysis (n=35)																		
Ballesio, 2017	Y	Y	Y	Partial Y	Y	Y	Y	Y	Y	Includes only RCTs	Y	Y	Y	Y	Y	Y	Y	High
Brasure, 2015	Y	Y	Y	Y	Y	N	Y	Y	Y	Includes only RCTs	Y	Y	Y	Y	Y	N	Y	High
Buscemi, 2004	Y	Partial Y	Y	Y	Y	Y	Y	Y	Partial Y	Includes only RCTs	Y	Y	N	Y	Y	Y	Y	High
Buscemi, 2005	Y	N	N	Partial Y	Y	N	Y	Partial Y	Y	Includes only RCTs	Y	Y	Y	N	Y	Y	Y	High
Cheng, 2012	Y	N	N	Y	N	N	N	Y	Y	Includes only RCTs	N	Y	Y	Y	N	N	Y	Low
Ferracioli, 2013	Y	N	N	N	Y	N	N	Partial Y	N	Includes only RCTs	N	Y	N	N	Y	Y	Y	Critically Low
Gong, 2016	Y	N	N	Partial Y	Y	Y	N	Partial Y	Y	Includes only RCTs	N	Y	N	N	Y	Y	Y	Low

Author, Year	1. PICO components	2. A priori design	3. Rationale for study selection	4. Literature search	5. Duplicate Selection	6. Duplicate Abstraction	7. List of excluded studies	8. Description of included studies	9a. RoB in RCTs	9b. RoB in non-randomized studies	10. Funding sources	11. Appropriate MA methods	12. Used RoB in MA	13. Used RoB in interpreting results	14. Discussion of heterogeneity	15. Publication bias	16. Conflict of Interest	Overall Rating
Ho, 2015	Y	N	N	Partial Y	Y	Y	N	Y	Y	Includes only RCTs	N	Y	Y	Y	N	Y	Y	Low
Ho, 2016	Y	N	Y	Partial Y	Y	Y	N	Partial Y	Y	Includes only RCTs	N	Y	N	N	N	N	Y	Low
Hwang, 2016	Y	N	N	Partial Y	Y	Y	N	N	Partial Y	N	N	Y	N	N	N	Y	N	Critically Low
Irwin, 2006	Y	N	N	Partial Y	N	N	N	Partial Y	N	Includes only RCTs	N	Y	N	N	Y	N	N	Critically Low
Johnson, 2016	Y	Y	Y	Y	Y	Y	N	Partial Y	Y	Includes only RCTs	N	Y	N	Y	Y	Y	Y	Moderate
Kishi, 2015	N	N	N	Partial Y	N	Y	N	Partial Y	Y	Includes only RCTs	N	Y	Y	Y	Y	Y	Y	Moderate
Koffel, 2015	N	N	Y	Partial Y	N	N	N	N	Y	Includes only RCTs	Y	N	N	N	N	Y	N	Critically Low
Kuriyama, 2017	N	N	N	Y	Y	Y	N	Y	Y	Includes only RCTs	N	Y	Y	Y	N	N	Y	Moderate
Lee, NR	Y	N	N	Y	Y	Y	N	Partial Y	Y	Includes only RCTs	N	Y	N	N	Y	N	Y	Critically Low

Author, Year	1. PICO components	2. A priori design	3. Rationale for study selection	4. Literature search	5. Duplicate Selection	6. Duplicate Abstraction	7. List of excluded studies	8. Description of included studies	9a. RoB in RCTs	9b. RoB in non-randomized studies	10. Funding sources	11. Appropriate MA methods	12. Used RoB in MA	13. Used RoB in interpreting results	14. Discussion of heterogeneity	15. Publication bias	16. Conflict of Interest	Overall Rating
Liu, 2017	Y	Partial Y	N	Partial Y	Y	N	N	Y	Y	Includes only RCTs	N	Y	N	N	Y	Y	Y	Critically Low
McCleery, 2016	Y	Partial Y	Y	Partial Y	Y	Y	Y	Partial Y	Partial Y	Includes only RCTs	N	Y	Y	Y	Y	N	Y	High
Montgomery, 2003	Y	Y	N	Y	Y	Y	Y	Y	Y	Includes only RCTs	N	Y	N	Y	Y	Y	Y	High
Navarro-Bravo, 2015	Y	N	N	Partial Y	Y	Y	N	Partial Y	Y	Includes only RCTs	N	Y	N	N	Y	Y	Y	Moderate
Okajima, 2011	Y	N	N	N	N	N	N	Y	N	Includes only RCTs	N	N	N	N	N	Y	N	Critically Low
Sateia, 2017	Y	N	N	N	Y	N	N	Partial Y	Partial Y	Y	Y	Y	N	Y	N	Y	Y	Critically Low
Seda, 2015	Y	N	N	Partial Y	N	N	N	Partial Y	N	Includes only RCTs	N	Y	N	N	Y	Y	N	Low
Seyffert, 2016	Y	Partial Y	N	Y	N	Y	N	Partial Y	Y	Includes only RCTs	Y	Y	Y	Y	N	Y	Y	Low
Soldatos, 2009	Y	N	N	N	N	N	Y	Partial Y	N	N	Y	Y	N	N	N	N	N	Critically Low

**Author, Year**	1. PICO components	2. A priori design	3. Rationale for study selection	4. Literature search	5. Duplicate Selection	6. Duplicate Abstraction	7. List of excluded studies	8. Description of included studies	9a. RoB in RCTs	9b. RoB in non-randomized studies	10. Funding sources	11. Appropriate MA methods	12. Used RoB in MA	13. Used RoB in interpreting results	14. Discussion of heterogeneity	15. Publication bias	16. Conflict of Interest	**Overall Rating**
Tang, 2015	Y	Y	N	Partial Y	Y	Y	N	Y	Y	Includes only RCTs	N	Y	N	Y	Y	Y	Y	Moderate
Trauer, 2015	Y	Y	N	Y	Y	Y	N	Partial Y	Y	Includes only RCTs	Y	Y	Y	Y	Y	Y	Y	Moderate
van Straten, 2007	Y	N	N	Y	Y	Y	N	Y	Y	Includes only RCTs	Y	N	Y	Y	Y	Y	Y	Low
van Straten, 2009	Y	N	N	Partial Y	N	N	N	Y	Partial Y	Includes only RCTs	N	Y	N	Y	Y	Y	N	Moderate
Xu, 2015	Y	N	N	Partial Y	Y	Y	N	Partial Y	Y	Includes only RCTs	N	Y	N	N	Y	Y	Y	Moderate
Yang, 2014	Y	Y	N	Partial Y	Y	Y	N	Y	Partial Y	Partial Y	N	Y	N	Y	Y	Y	Y	Moderate
Ye, 2016	Y	Partial Y	N	Partial Y	Y	Y	Y	Partial Y	Partial Y	Includes only RCTs	Y	Y	N	Y	Y	Y	Y	Moderate
Yuan, 2010	Y	N	N	Partial Y	Y	Y	N	Partial Y	Y	Includes only RCTs	Y	Y	N	N	Y	Y	N	Low
Zachariae, 2016	Y	Y	N	Partial Y	Y	N	Y	Partial Y	Y	Includes only RCTs	N	Y	Y	Y	Y	Y	Y	Moderate

Author, Year	1. PICO components	2. A priori design	3. Rationale for study selection	4. Literature search	5. Duplicate Selection	6. Duplicate Abstraction	7. List of excluded studies	8. Description of included studies	9a. RoB in RCTs	9b. RoB in non-randomized studies	10. Funding sources	11. Appropriate MA methods	12. Used RoB in MA	13. Used RoB in interpreting results	14. Discussion of heterogeneity	15. Publication bias	16. Conflict of Interest	Overall Rating
Zhang, 2016	Y	N	N	Partial Y	N	Y	N	Partial Y	Y	Includes only RCTs	N	Y	N	N	N	Y	Y	Critically Low
**Systematic reviews without meta-analysis (n=29)**																		
Anderson, 2014	N	N	N	Partial Y	N	N	N	Partial Y	N	N	N	No MA	No MA	N	N	No MA	N	Critically Low
Bellon, 2006	N	N	N	Partial Y	N	N	N	Partial Y	N	N	N	No MA	No MA	N	N	No MA	N	Critically Low
Bogdanov, 2017	N	N	N	Partial Y	N	N	N	Partial Y	Y	Y	N	No MA	No MA	N	N	No MA	Y	Low
Brooks, 2014	N	N	N	Partial Y	N	N	N	Partial Y	Partial Y	Partial Y	N	No MA	No MA	Y	N	No MA	Y	Low
Chase, 1997	N	N	N	N	N	N	N	Partial Y	N	N	N	No MA	No MA	N	N	No MA	N	Critically Low
Chiesa, 2009	Y	N	Y	Partial Y	N	N	N	Partial Y	Y	Y	N	No MA	No MA	N	N	No MA	N	Low
Cimolai, 2007	N	N	N	N	N	N	N	Partial Y	N	N	N	No MA	No MA	N	N	No MA	Y	Critically Low
Citrome, 2014	N	N	N	Partial Y	N	N	N	Partial Y	N	N	N	No MA	No MA	N	No MA	No MA	Y	Critically Low
Coe, 2012	N	N	N	Partial Y	N	N	N	Partial Y	N	N	N	No MA	No MA	N	N	No MA	Y	Critically Low
Costello, 2014	Y	N	Y	Partial Y	Y	N	N	Y	Y	Includes only RCTs	Y	No MA	No MA	Y	Y	No MA	Y	Moderate
Culpepper, 2015	Y	Partial Y	Y	Partial Y	N	N	N	Partial Y	N	Includes only RCTs	N	No MA	No MA	N	N	No MA	Y	Critically Low

**Author, Year**	1. PICO components	2. A priori design	3. Rationale for study selection	4. Literature search	5. Duplicate Selection	6. Duplicate Abstraction	7. List of excluded studies	8. Description of included studies	9a. RoB in RCTs	9b. RoB in non-randomized studies	10. Funding sources	11. Appropriate MA methods	12. Used RoB in MA	13. Used RoB in interpreting results	14. Discussion of heterogeneity	15. Publication bias	16. Conflict of Interest	**Overall Rating**
Dickerson, 2014	Y	Partial Y	Y	Partial Y	Y	Y	Partial Y	Partial Y	Partial Y	Includes only RCTs	N	No MA	No MA	Y	Y	No MA	Y	High
Hellstrom, 2011	N	Partial Y	N	Partial Y	N	N	Y	Y	Y	Includes only RCTs	N	No MA	No MA	Y	N	No MA	N	Moderate
Howell, 2014	N	Partial Y	N	Partial Y	Y	N	N	Partial Y	Y	Partial Y	Y	No MA	No MA	N	N	No MA	Y	Moderate
Ishak, 2012	N	Partial Y	N	Partial Y	Y	N	N	Partial Y	N	N	N	No MA	No MA	N	N	No MA	Y	Low
Kolla, 2011	N	N	N	Partial Y	N	N	N	Partial Y	N	N	N	No MA	No MA	N	N	No MA	N	Critically Low
Mayers, 2005	Y	N	Y	Partial Y	N	N	N	Partial Y	N	Includes only RCTs	N	No MA	No MA	N	N	No MA	N	Critically Low
McCurry, 2007	Y	N	N	N	N	Y	N	Partial Y	N	N	N	No MA	No MA	N	N	No MA	N	Critically Low
Mendelson, 2005	N	N	N	Partial Y	N	N	N	Partial Y	N	N	N	No MA	No MA	N	N	No MA	N	Critically Low
Miller, 2014	Y	N	Y	Partial Y	N	N	Y	Partial Y	Y	Y	N	No MA	No MA	Y	N	No MA	Y	High
Swainston Harrison, 2005	Y	Partial Y	Y	Partial Y	N	N	N	Partial Y	N	N	N	No MA	No MA	N	N	No MA	N	Critically Low
Tamrat, 2013	Y	Partial Y	N	N	Y	Y	N	Partial Y	Y	Y	N	No MA	No MA	Y	Y	No MA	Y	Low
Taylor, 2014	N	N	N	Partial Y	N	N	N	Partial Y	N	Includes only RCTs	N	No MA	No MA	N	N	N	Y	Critically Low

Author, Year	1. PICO components	2. A priori design	3. Rationale for study selection	4. Literature search	5. Duplicate Selection	6. Duplicate Abstraction	7. List of excluded studies	8. Description of included studies	9a. RoB in RCTs	9b. RoB in non-randomized studies	10. Funding sources	11. Appropriate MA methods	12. Used RoB in MA	13. Used RoB in interpreting results	14. Discussion of heterogeneity	15. Publication bias	16. Conflict of Interest	Overall Rating
Vande Griend, 2012	N	N	N	Partial Y	N	N	N	Partial Y	N	Includes only RCTs	N	No MA	No MA	N	N	N	Y	Critically Low
Venables, 2014	N	N	N	Partial Y	N	N	N	Y	N	N	N	No MA	No MA	N	N	No MA	N	Critically Low
Vural, 2014	Y	N	N	Partial Y	N	N	Partial Y	N	N	N	N	No MA	No MA	N	N	No MA	Y	Critically Low
Wang, 2005	Y	Partial Y	Y	Partial Y	Y	Y	N	Partial Y	Partial Y	Includes only RCTs	N	No MA	No MA	Y	Y	No MA	N	Moderate
Wine, 2009	N	N	N	Partial Y	N	N	N	Partial Y	N	N	N	No MA	No MA	N	N	No MA	N	Critically Low
Yeung, 2015	Y	Partial Y	N	Y	Y	Y	N	Y	Y	Includes only RCTs	Y	No MA	No MA	Y	Y	No MA	Y	Moderate

Appendix E: Detailed Tables of Results

Table E1. Benzodiazepines

Author, Year<br>Synthesis type	AMSTAR rating	Intervention and sample size	# of studies	Measurement method	Pooled Estimates (MA) or<br>Narrative Results (from SR with no MA)*
Sleep onset latency					
Buscemi, 2005<br>Meta-analysis	High	Flurazepam: 317<br><br>Placebo: 215	10	sleep diary, polysomnography	Mean difference [95% CI]:<br>**-23.21[-34.26, -12.16]** I-squared: 51.8%
		Temazepam: 128<br><br>Placebo: 78	4	sleep diary, polysomnography	Mean difference [95% CI]:<br>-11.61 [-23.64, 0.42] I-squared: 84%
		Triazolam: 290<br><br>Placebo: 249	8	sleep diary, polysomnography	Mean difference [95% CI]:<br>**-19.69 [-28.36, -11.01]** I-squared: 69.2%
Sateia, 2017<br>Meta-analysis	Critically Low	Temazepam: 36<br><br>Placebo: 36	2	subjective measure	Mean difference [95% CI]:<br>**-20.06 [-39.05, -1.07]** I-squared: 68%
Soldatos, 1999<br>Meta-analysis	Critically Low	Triazolam: NR<br><br>Placebo: NR	28	sleep laboratory	Mean difference [95% CI]:<br>**-15.5 [-19.5, -11.4]** I-squared: NR
Total sleep time					
Sateia, 2017<br>Meta-analysis	Critically Low	Temazepam: 36<br><br>Placebo: 36	2	subjective measure	Mean difference [95% CI]:<br>**64.41 [8.07, 120.76]** I-squared: 59%
Soldatos, 1999<br>Meta-analysis	Critically Low	Triazolam: NR<br><br>Placebo: NR	12	sleep laboratory	Mean difference [95% CI]:<br>**49.2 [36, 62.5]** I-squared: NR
Kolla, 2011<br>Systematic Review	Critically Low	Triazolam:<br>23 (enrolled); 12 (analysis)<br><br>No comparator (pre- and post-intervention)	1	sleep diaries	**Significant improvement** in depth and duration of sleep

Author, Year Synthesis type	AMSTAR rating	Intervention and sample size	# of studies	Measurement method	Pooled Estimates (MA) or Narrative Results (from SR with no MA)*
Swainston Harrison, 2005 Systematic Review	Critically Low	Triazolam<br>Placebo<br>Total sample: 16	1	NR	Change in outcome (p-value): **+41 vs +25 minutes (p<0.05)**
Wake after sleep onset					
Buscemi, 2005 Meta-analysis	High	Temazepam: 38<br>Placebo: 39	2	sleep diary, polysomnography	Mean difference [95% CI]: **-23.66 [-36.57, -10.76]** I-squared: 0%
		Triazolam: 30<br>Placebo: 27	2	sleep diary, polysomnography	Mean difference [95% CI]: **-39.96 [-64.47, -15.45]** I-squared: 0%
Sleep quality					
Sateia, 2017 Meta-analysis	Critically Low	Temazepam: 39<br>Placebo: 39	2	subjective measure	Mean difference [95% CI]: 0.25 [-0.20, 0.70] I-squared: 0%

* bolded results indicate statistical significance as reported by review

Table E2. Non-benzodiazepines

Author, Year Synthesis type	AMSTAR rating	Intervention and sample size	# of studies	Measurement method	Pooled Estimates (MA) or Narrative Results (from SR with no MA)
Sleep onset latency					
Brasure, 2015 Meta-analysis	High	Zolpidem: 181<br>Placebo: 192	4	subjective sleep latency, minutes	Mean difference [95% CI]: **-14.95 [-22.10, -7.80]** I-squared: 0%
		Zolpidem: 177<br>Placebo: 178	2	subjective report, minutes	Mean difference [95% CI]: **-14.8 [-23.41, -6.19]** I-squared: 0%
Buscemi, 2005 Meta-analysis	High	Zolpidem: 997<br>Placebo: 808	17	sleep diary, polysomnography	Mean difference [95% CI]: **-12.75 [-16.42, -9.08]** I-squared: 4.5%
		Zopiclone: 178<br>Placebo: 178	5	sleep diary, polysomnography	Mean difference [95% CI]: **-30.91 [-49.37, -12.44]** I-squared: 73.9%
Sateia, 2017 Meta-analysis	Critically Low	Zolpidem: 181<br>Placebo: 185	5	polysomnography	Mean difference [95% CI]: **-11.65 [-19.15, -4.15]** I-squared: 78%

Author, Year Synthesis type	AMSTAR rating	Intervention and sample size	# of studies	Measurement method	Pooled Estimates (MA) or Narrative Results (from SR with no MA)
		Zolpidem: 543<br><br>Placebo: 558	10	subjective measure	Mean difference [95% CI]:<br>**-19.55 [-24.90, -14.20]** I-squared: 95%
Soldatos, 1999<br>Meta-analysis	Critically Low	Zolpidem: NR<br><br>Placebo: NR	29	sleep laboratory	Mean difference [95% CI]:<br>**-17.6 [-23.2, -12]** I-squared: NR
		Zopiclone: NR<br><br>Placebo: NR	14	sleep laboratory	Mean difference [95% CI]:<br>**-19.1 [-26.7, -11.5]** I-squared: NR
Mayers, 2005<br>Systematic Review	Critically Low	Zolpidem<br>Placebo<br><br>Total sample: 306	1	NR	**Significant decrease** in sleep latency compared to placebo **(p<0.05)**
Mendelson, 2005<br>**Systematic Review**	Critically Low	Zolpidem<br>Placebo<br><br>Total sample: 306	1	self-reported	The zolpidem group demonstrated **significant improvemen**t compared with placebo for sleep latency **(p = 0.037)**
Swainston Harrison, 2005<br>**Systematic Review**	Critically Low	Zolpidem<br>Triazolam<br><br>Total sample: 22	1	NR	Change in outcome (p-value):<br>-23 v -15 minutes (p=NS)
Total sleep time					
Brasure, 2015<br>Meta-analysis	High	Zolpidem: 82<br>Placebo: 85	3	subjective report	Mean difference [95% CI]:<br>**22.95 [2.01, 43.88]** I-squared: 0%
Sateia, 2017<br>Meta-analysis	Critically Low	Zolpidem: 55<br>Placebo: 57	2	PSG	Mean difference [95% CI]:<br>**28.91 [10.85, 46.97]** I-squared: 49%
		Zolpidem: 435<br>Placebo: 455	8	subjective measure	Mean difference [95% CI]:<br>**30.04 [15.12, 44.96]** I-squared: 71%
Soldatos, 1999<br>Meta-analysis	Critically Low	Zolpidem: NR<br>Placebo: NR	23	sleep laboratory	Mean difference [95% CI]:<br>**32 [21.7, 42.3]**I-squared: NR
		Zopiclone: NR<br>Placebo: NR	13	sleep laboratory	Mean difference [95% CI]:<br>**56.3 [37.3, 75.4]** I-squared: NR
Mayers, 2005<br>Systematic Review	Critically Low	Zolpidem<br>Placebo<br>Total sample: 306	1	NR	**Significant increase** in total sleep time compared to placebo **(p<0.05)**
Swainston Harrison, 2005<br>Systematic Review	Critically Low	Zolpidem<br>Placebo<br>Total sample: 16	1	NR	Change in outcome (p-value):<br>**+35 vs +29 minutes (p<0.05)**

Author, Year Synthesis type	AMSTAR rating	Intervention and sample size	# of studies	Measurement method	Pooled Estimates (MA) or Narrative Results (from SR with no MA)
	Critically Low	Zolpidem<br>Triazolam<br>Total sample: 16	1	NR	Change in outcome (p-value):<br>+35 vs -112 minutes (p<0.05)
Wake after sleep onset					
Buscemi, 2005<br>Meta-analysis	High	Zolpidem: 345<br>Placebo: 345	7	sleep diary<br>polysomnography	Mean difference [95% CI]:<br>-8.46[-20.17, 3.26] I-squared: 64.1%
Sateia, 2017<br>Meta-analysis	Critically Low	Zolpidem: 55<br>Placebo: 57	2	polysomnography	Mean difference [95% CI]:<br>**-25.46 [-32.99, -17.94]** I-squared: 0%
		Zolpidem: 384<br>Placebo: 400	6	subjective measure	Mean difference [95% CI]:<br>**-13.57 [-19.84, -7.30]** I-squared: 92%
Mayers, 2005<br>Systematic Review	Critically Low	Zolpidem<br>Placebo<br>Total sample: 306	1	NR	**Significant improvement** in WASO compared to placebo **(p=0.04)**
Mendelson, 2005<br>Systematic Review	Critically Low	Zolpidem<br>Placebo<br>Total sample: 306	1	self-reported	Relative to placebo, patients reported **significant improvement** in [WASO] with trazodone and zolpidem during week 1 **(p < .02)**
Swainston Harrison, 2005<br>Systematic Review	Critically Low	Zolpidem: 16<br>Placebo: 69	2	NR	Change in outcome (p-value):<br>**-35 vs +116 min (p<0.05)**<br>+6 vs -8 min (p=NS)
	Critically Low	Zolpidem<br>Triazolam<br>Total sample: 102	3	NR	Change in outcome (p-value):<br>**-35 vs +116 min (p<0.05)**;<br>**-38 vs +17 min (p<0.01)**;<br>+6 vs +19 min (p=NS)
Sleep Quality					
Brasure, 2015<br>Meta-analysis	High	Zolpidem: 289<br>Placebo: 268	3	participants reporting improvement	Mean difference [95% CI]:<br>**1.4 [1.20, 1.65]** I-squared: 14%
Sateia, 2017<br>Meta-analysis	Critically Low	Zolpidem: 314<br>Placebo: 324	6	subjective measure	Standardized mean difference [95% CI]:<br>**0.64 [0.03, 1.26]** I-squared: 92%
Mayers, 2005<br>Systematic Review	Critically Low	Zolpidem<br>Placebo<br>Total sample: 306	1	NR	**Significant improvement** on sleep quality compared to placebo **(p=0.0003)**
Mendelson, 2005<br>Systematic Review	Critically Low	Zolpidem<br>Placebo<br>Total sample: 306	1	self-reported	Relative to placebo, patients reported **significant improvement** in [sleep quality] with zolpidem during week 1 **(p < 0.02)**
Sleep efficiency					

Author, Year<br>Synthesis type	AMSTAR rating	Intervention and sample size	# of studies	Measurement method	Pooled Estimates (MA) or<br>Narrative Results (from SR with no MA)
Sateia, 2017<br>Meta-analysis	Critically Low	Zolpidem: 111<br>Placebo: 115	4	polysomnography	Mean difference[95% CI]:<br>**6.12 [4.39, 7.85]** I-squared: 35%
Swainston Harrison, 2005<br>Systematic Review	Critically Low	Zolpidem<br>Placebo<br>Total sample: 69	1	NR	Change in outcome (p-value):<br>-3 vs +5 min (p=NS)
	Critically Low	Zolpidem<br>Triazolam<br>Total sample: 86	2	NR	Change in outcome (p-value):<br>**+10 vs -6 min (p<0.01)**<br>-3 vs -15 min (p=NS)
Health-related quality of life					
Ishak, 2012<br>Systematic Review	Low	Zopiclone<br>Placebo<br>Total sample: 1006	2	23-item questionnaire developed by sleep experts; QOLI	Contradicting evidence between both studies. No differences found in QoL between subjects treated with zopiclone and placebo
	Low	Zolpidem (5 nights/week) or Zolpidem (nightly)<br>Total sample: 789	1	SF-36	Both groups demonstrated **improvement** with treatment **(p=0.005)**. The continuous group demonstrated greater increase in mean MOS than the discontinuous group.
Hangover/morning sedation					
Cimolai, 2007<br>Systematic Review	Critically Low	Zopiclone<br>Flurazepam<br>Placebo<br>Total sample: 24	1	NR	After 3 weeks of treatment, zopiclone has no effect on early morning performance and free of residual sedative activity
Addiction, dependence, or diversion					
Cimolai, 2007<br>Systematic Review	Critically Low	Zopiclone<br>No comparator (pre- post-intervention)<br>Total sample: 119	3	NR	One study found no carry over effect after 3 weeks of treatment; the second one found that after 7-8 weeks of treatment, return of sleep variables to pre-treatment baseline after withdrawal, and 1 of 11 patients had marked rebound insomnia and daytime anxiety for the first week off; and the third study after 3 mos of treatment, found withdrawal effects despite tapering dose
		Zopiclone<br>Triazolam<br>Total sample: 48	1	NR	Worse psychomotor deterioration after triazolam than zopiclone, 3 of 24 zopiclone patients felt agitated early after withdrawal
		Zopiclone<br>Zolpidem<br>Total sample: 248	1	NR	After 2 weeks of treatment, zopiclone group had 15.4% with rebound insomnia

Author, Year Synthesis type	AMSTAR rating	Intervention and sample size	# of studies	Measurement method	Pooled Estimates (MA) or Narrative Results (from SR with no MA)
		Zopiclone<br>Temazepam<br>Placebo<br>Total sample: 35	2	NR	No psychomotor performance deterioration after 2 weeks of treatment; no rebound insomnia or anxiety after 3 weeks of treatment

* bolded results indicate statistical significance as reported by review

Table E3. Suvorexant

Author, Year Synthesis type	AMSTAR rating	Intervention and sample size	# of studies	Measurement method	Pooled Estimates (MA) or Narrative Results (from SR with no MA)
Sleep onset latency					
Brasure, 2015<br>Meta-analysis	High	Suvorexant: 425<br><br>Placebo: 664	2	subjective report	Mean change [95% CI]:<br>**-5.97 [-10.01, -1.92]** I-squared: 0%
Kishi, 2015<br>Meta-analysis	Moderate	Suvorexant: 936<br>Placebo: 953	3	sleep diary	Mean difference [95% CI]:<br>**-7.62 [-11.03, -4.21]** I-squared: 0%
		Suvorexant: 349<br>Placebo: 659	3	polysomnography	Mean difference [95% CI]:<br>**-10.82 [-16.72, -4.93]** I-squared: 35%
Kuriyama, 2017<br>Meta-analysis	Moderate	Suvorexant: NR<br>Placebo: NR	3	sleep diary, PSG	Mean difference [95% CI]:<br>**-9.45 [-13.26, -5.65]** I-squared: 13.3%
		Suvorexant: NR<br>Placebo: NR	3	polysomnography	Mean difference [95% CI]:<br>-6.39 [-12.85, 0.07] I-squared: 67.1%
Citrome, 2014<br>Systematic Review	Critically Low	Suvorexant; 15; 20 mg: 425<br>Placebo: 688	2	sleep diary	Change in outcome [least squares mean difference (p-value)]:<br>**-5.9 minutes (p<0.01)**<br>Proportion of respondents with >15% improvement:<br>69.9% v 66%; NNT: 26 (p=NS)<br>Suvorexant was **superior to placebo** for sleep latency both through patient-assessed and polysomnography means
		Suvorexant; 15; 20 mg: 343<br>Placebo: 585	2	polysomnography	Change in outcome [least squares mean difference (p-value)]:<br>-4.6 minutes (p=NS)
		Suvorexant; 30; 40 mg: 688<br>Placebo: 664	2	sleep diary	Change in outcome [least squares mean difference (p-value)]:<br>**-10.8 minutes (p<0.001)**<br>Proportion of respondents with >15% improvement:<br>76.5% v 66%; NNT 10 (95% CI 7 to 18)
		Suvorexant; 30; 40 mg: 590<br>Placebo: 585	2	polysomnography	Change in outcome [least squares mean difference (p-value)]:<br>**-6.4 minutes (p<0.01)**

Total sleep time					
Kishi, 2015<br>Meta-analysis	Moderate	Suvorexant: 936<br>Placebo: 953	3	sleep diary	Mean difference [95% CI]:<br>**-20.16 min[-25.01, -15.30]** I-squared: 0%
Kuriyama, 2017<br>Meta-analysis	Moderate	Suvorexant: NR<br>Placebo: NR	3	sleep diary, PSG	Mean difference [95% CI]:<br>**18.55 min [12.52, 24.58]** I-squared: 0%
Brasure, 2015<br>Meta-analysis	High	Suvorexant: 425<br>Placebo: 664	2	subjective report	Mean change [95% CI]:<br>**15.97 min [4.73, 27.22]** I-squared: 63%
Citrome, 2014<br>Systematic Review	Critically Low	Suvorexant<br>(15, 20 mg): 425<br>Placebo: 664	2	sleep diary	Change in outcome [least squares mean difference (p-value)]:<br>**16 minutes (p<0.001)**<br>Proportion of respondents with >15% increase:<br>50.1% v 41.9%; NNT 13 (95% CI 17 to 46)<br>Suvorexant was also **superior to placebo** for sleep maintenance, as assessed subjectively by patient-estimated total sleep time
		Suvorexant<br>(30, 40 mg): 688<br>Placebo: 664	2	sleep diary	Change in outcome: [least squares mean difference (p-value)]:<br>**22.1 minutes (p<0.0001)**<br>Proportion of respondents with >15% increase:<br>54.7% v 41.9%; NNT 8 (95% CI 6 to 14)
Wake after sleep onset					
Kishi, 2015<br>Meta-analysis	Moderate	Suvorexant: 955<br>Placebo: 960	3	sleep diary	Mean difference [95% CI]:<br>**-7.75 [-10.87, -4.62]** I-squared: 0%
		Suvorexant: 317<br>Placebo: 542	2	polysomnography	Mean difference [95% CI]:<br>**-25.32 [-31.52, -19.39]** I-squared: 0%
Kuriyama, 2017<br>Meta-analysis	Moderate	Suvorexant: NR<br>Placebo: NR	3	sleep diary	Mean difference [95% CI]:<br>**-7.51 [-12.46, -2.56]** I-squared: 0%
		Suvorexant: NR<br>Placebo: NR	3	polysomnography	Mean difference [95% CI]:<br>**-24.19 [-33.81, -14.58]** I-squared: 69.7%
Citrome, 2014<br>Systematic Review	Critically Low	Suvorexant<br>(15, 20 mg): 425<br>Placebo: 660	2	polysomnography	Change in outcome [least squares mean difference (p-value)]:<br>**-4.7 minutes (p<0.001)**<br>Proportion of respondents with >15% improvement:<br>75.8% v 69.4%; NNT 16 (95% CI 9 to 102)
		Suvorexant<br>(15, 20 mg): 343<br>Placebo: 585	2	polysomnography	Change in outcome: [least squares mean difference (p-value)]:<br>**-23.1 minutes (p<0.001)**
		Suvorexant<br>(30, 40 mg): 683<br>Placebo: 660	2	sleep diary	Change in outcome [least squares mean difference (p-value)]:<br>**-7.8 minutes (p<0.001)**<br>Proportion of respondents with >15% improvement:<br>77.5% v 69.4%; NNT 13 (95% CI 8 to 30)

		Suvorexant<br>(30, 40 mg): 590<br>Placebo: 585	2	polysomnography	Change in outcome: [least squares mean difference (p-value):<br>**-25.9 minutes (p<0.001)**
Sleep quality					
Kishi, 2015<br>Meta-analysis	Moderate	Suvorexant: 955<br>Placebo: 960	2	Sleep diary; 4-point scale	Mean difference [95% CI]:<br>**-0.17 [-0.25, -0.09]** I-squared: 0%
Insomnia severity index					
Kishi, 2015<br>Meta-analysis	Moderate	Suvorexant: 947<br>Placebo: 952	3	ISI	Mean difference [95% CI]:<br>**-1.35 [-1.78, -0.93]** I-squared: 0%
Kuriyama, 2017<br>Meta-analysis	Moderate	Suvorexant: NR<br>Placebo: NR	3	ISI	Mean difference [95% CI]:<br>**-1.42 [-1.85, -0.98]** I-squared: 0%
Citrome, 2014<br>Systematic Review	Critically Low	Suvorexant<br>(15, 20 mg): 411<br>Placebo: 638	2	ISI	Proportion of respondents with >6 point improvement:<br>55.5% v 42.2%; NNT 8 (95% CI 6 to 14)
		Suvorexant<br>(30, 40 mg): 656<br>Placebo: 638	2	ISI	Proportion of respondents with >6 point improvement:<br>54.9% v 42.2%; NNT 8 (95% CI 6 to 14)
Hangover/morning sedation					
Kishi, 2015<br>Meta-analysis	Moderate	Suvorexant: 1784<br>Placebo: 1025	3	NR	Risk ratio [95% CI]:<br>**3.34 [1.08, 10.32]** I-squared: 0%
Kuriyama, 2017<br>Meta-analysis	Moderate	Suvorexant: 2027<br>Placebo: 1274	3	"excessive daytime sleepiness"	Relative risk [95% CI]:<br>**3.05 [1.10, 8.48]** I-squared: 0%
Accidental Injury					
Kishi, 2015<br>Meta-analysis	Moderate	Suvorexant: 1784<br>Placebo: 1025	3	"Motor vehicle accidents/violations"	Risk ratio [95% CI]:<br>**1.16 [0.52, 2.60]** I-squared: 14%
Kuriyama, 2017<br>Meta-analysis	Moderate	Suvorexant: 1784<br>Placebo: 1025	3	"Falls"	Relative risk [95% CI]:<br>**0.84 [0.44, 1.62]** I-squared: 0%
		Suvorexant: 1784<br>Placebo: 1025	3	"Motor vehicle accident/violation"	Relative risk [95% CI]:<br>**1.16 [0.39, 3.40]** I-squared: 50.2%
Addiction, dependence, diversion					
Kishi, 2015<br>Meta-analysis	Moderate	Suvorexant: 1784<br>Placebo: 1025	3	"events suggesting drug abuse potential"	Risk ratio [95% CI]:<br>**1.05 [0.67, 1.65]** I-squared: 0%
Kuriyama, 2017<br>Meta-analysis	Moderate	Suvorexant: 1784<br>Placebo: 1025	3	"Potential drug abuse"	Relative risk [95% CI]:<br>**1.05 [0.66, 1.65]** I-squared: 0%

Table E4. Antidepressants

Author, Year Synthesis type	AMSTAR rating	Intervention and sample size	# of studies	Measurement method	Pooled Estimates (MA) or Narrative Results (from SR with no MA)
Sleep onset latency					
Buscemi, 2005 Meta-analysis	High	Doxepin 25mg: 40<br>Placebo: 40	3	sleep diary, polysomnography	Mean difference [95% CI]:<br>**-6.65 [-10.68, -2.63]** I-squared: 49.3%
Sateia, 2017 Meta-analysis	Critically Low	Doxepin 3mg: 282<br>Placebo: 276	4	polysomnography	Mean difference [95% CI]:<br>-2.3 [-6.22, 1.62] I-squared: 0%
		Doxepin 3mg: 148<br>Placebo: 143	2	subjective measure	Mean difference [95% CI]:<br>-9.35 [-21.89, 3.19] I-squared: 55%
		Doxepin 6mg: 209<br>Placebo: 206	3	polysomnography	Mean difference [95% CI]:<br>**-5.29 [-9.25, -1.34]** I-squared: 0%
Yuan, 2010 Meta-analysis	Low	Doxepin 1mg: 140<br>Placebo: 139	2	NR	Mean difference [95% CI]:<br>-0.85 [-5.82, 4.13] I-squared: NR
		Doxepin 3mg: 140<br>Placebo: 139	2	NR	Mean difference [95% CI]:<br>0.37 [-0.66, 1.40] I-squared: NR
		Doxepin 6mg: 141<br>Placebo: 139	2	NR	Mean difference [95% CI]:<br>0.37 [-0.66 to 1.40] I-squared: NR
		Doxepin 25mg: 30<br>Placebo: 30	2	NR	Mean difference [95% CI]:<br>**-8.69 [-13.72 to -3.67]** I-squared: NR
Buscemi, 2005 Meta-analysis	High	Trazodone (50mg, 150-250mg): 100<br>Placebo: 108	2	sleep diary, polysomnography	Mean difference [95% CI]:<br>**-12.21 [-22.26, -2.15]** I-squared: 0%
Mayers, 2005 Systematic Review	Critically Low	Doxepin<br>Placebo<br>Total sample: 10	1	NR	Doxepin **significantly improved** sleep latency compared to placebo (p-value not reported)
Vande Griend, 2012 Systematic Review	Critically Low	Doxepin: NR<br>Placebo: NR	6	polysomnography questionnaire (7-point Likert scale)	Mixed results: polysomnography and questionnaire data showed **significant improvement** compared to placebo in some trials **(p<0.05)**; non-significant change found in other trials
Yeung, 2015 Systematic Review	Moderate	Doxepin: NR<br>Placebo: NR	NR	self-report	Adults <65 y: 3 mg doxepin had negative impact on sleep latency in short term (1-2 nights) results, 6 mg doxepin had positive impact on sleep latency in the short term (1-2 nights)<br>Adults >65y: 3 mg doxepin had negative impact on sleep-latency in both short- and long-term (4 weeks) treatment; 6 mg doxepin had positive results at short-term but negative results at long-term

			NR	polysomnography	Adults <65y: 3 and 6 mg had mixed results in short-term and negative results in long-term<br><br>Adults >65y: 3 mg doxepin had mixed results in short term and negative results in long-term; 6 mg doxepin had negative results in short term, no assessment of long-term effect
Mayers, 2005<br>Systematic Review	Critically Low	Trazodone<br>Placebo<br>Total sample: 323	2	NR	**Significant decrease** in sleep latency for one trial **(p<0.05)**, almost significant change in the other (p=0.06)
Mendelson, 2005<br>Systematic Review	Critically Low	Trazodone<br>Placebo; unspecified control<br>Total sample: 306	1	self-reported	Relative to placebo, patients reported **significant improvement** during week 1 **(p<0.02)**; during week 2, the trazodone group did not differ significantly from the placebo group.
		Trazodone<br>placebo; unspecified control<br>Total sample: 29	2	polysomnography	No significant changes in sleep latency found between groups
		Trazodone: 39<br>No comparator (pre- post-intervention)	5	polysomnography	Mixed results: 3 trials found **significant improvement** compared to baseline **(p<0.05)**; 2 trials found non-significant change
Vande Griend, 2012<br>Systematic Review	Critically Low	Trazodone: NR<br>Placebo: NR	3	sleep diaries; polysomnography	No significant difference found between groups
Mayers, 2005<br>Systematic Review	Critically Low	Trazodone<br>Zolpidem<br>Total sample: 306	1	NR	Sleep latency was **significantly shorter** for the zolpidem v placebo group compared to trazodone v placebo group **(p=0.037)**
Mendelson, 2005<br>Systematic Review	Critically Low	Trazodone<br>Zolpidem<br>Total sample: 306	1	self-reported	Sleep latency for zolpidem compared to placebo was **significantly shorter** than that for trazodone compared to placebo **(p <0.037)**
Vande Griend, 2012<br>Systematic Review	Critically Low	Trazodone<br>Zolpidem<br>Total sample: 306	1	daily questionnaire	Sleep latency was **significantly shorter** for zolpidem compared to placebo **(p<0.005)** but not for trazodone compared with placebo; no significant difference between zolpidem and trazodone
Total sleep time					
Brasure, 2015<br>Meta-analysis	High	Doxepin: 289<br>Placebo: 205	2	subjective report, in minutes	Mean change [95% CI]:<br>**23.85 [12.04, 35.65]** I-squared: 0%
Liu, 2017<br>Meta-analysis	Critically Low	Doxepin: 743<br>Placebo: 733	7	polysomnography	Standardized mean difference [95% CI]:<br>**0.61 [0.50, 0.71]** I-squared: 15%

Sateia, 2017<br>Meta-analysis	Critically Low	Doxepin (3mg): 282<br>Placebo: 276	4	polysomnography	Mean difference [95% CI]:<br>**26.14 [18.49, 33.79]** I-squared: 0%
		Doxepin (3mg): 148<br>Placebo: 143	2	subjective measure	Mean difference [95% CI]:<br>**43.57 [5.16, 81.98]** I-squared: 82%
		Doxepin (6mg): 209<br>Placebo: 206	3	PSG	Mean difference [95% CI]:<br>**32.27 [24.24, 40.30]** I-squared: 0%
		Doxepin (6mg): 204<br>Placebo: 197	2	subjective measure	Mean difference [95% CI]:<br>18.84 [-1.65, 39.34] I-squared: 56%
Yuan, 2010<br>Meta-analysis	Low	Doxepin (1mg): 140<br>Placebo: 139	2	NR	Mean difference [95% CI]:<br>**17.24 [7.43, 27.05]** I-squared: NR
		Doxepin (25mg): 30<br>Placebo: 30	2	NR	Mean difference [95% CI]:<br>**70.74 [42.61, 98.88]** I-squared: NR
		Doxepin (3mg): 140<br>Placebo: 139	2	NR	Mean difference [95% CI]:<br>**27.95 [17.99, 37.90]** I-squared: NR
		Doxepin (6mg): 141<br>Placebo: 139	2	NR	Mean difference [95% CI]:<br>**33.78 [24.44, 43.11]** I-squared: NR
Mayers, 2005<br>Systematic Review	Critically Low	Doxepin<br>Placebo<br>Total sample: 10	1	NR	Doxepin **significantly improved** total sleep time compared to placebo (p-value not reported)
Vande Griend, 2012<br>Systematic Review	Critically Low	Doxepin: NR<br>Placebo: NR	7	polysomnography<br>questionnaire<br>(Likert-type scale)	Doxepin increased total sleep time by 25-51 minutes across the trials; 6 of the found a **significant difference** compared to placebo **(p<0.05)**
Yeung, 2015<br>Systematic Review	Moderate	Doxepin: NR<br>Placebo: NR	NR	self-report	Adults <65y: mixed results for 3 mg doxepin over 1-2 nights, a single trial showed improvement at 4 weeks; multiple trials showed positive improvement for 6 mg doxepin over 1-2 nights, a single trial showed improvement at 4 weeks<br>Adults >65y: 3mg and 6mg doxepin showed positive results over 1-2 nights; results were maintained for 3 mg doxepin only
			NR	polysomnography	Adults <65y: multiple trials found positive results for 3mg and 6mg doxepin over 1-2 nights, the effect was maintained at 4 weeks for 6mg doxepin only<br><br>Adults >65y: positive results for 3mg and 6mg doxepin over 1-2 nights, the effect was maintained at 4 weeks for 3 mg doxepin; no long-term data on 6mg doxepin was available

Mayers, 2005<br>Systematic Review	Critically Low	Trazodone<br>Placebo<br>Total sample: 323	2	NR	Both trials found a **significant increase** in total sleep time compared to placebo **(p<0.05; p=0.003)**
		Trazodone (50 v 75 v 100mg): 75<br>No comparator<br>(multiple doses)	1	self-rated	Total sleep time was **significantly longer** with 50mg and 75mg doses compared to 100mg (p-value NR); no significant difference was found between 50mg and 75 mg doses (p-value NR)
Mendelson, 2005<br>Systematic Review	Critically Low	Trazodone<br>placebo; unspecified control<br>Total sample: 63	3	polysomnography	Mixed results: 2 trials found **significant improvement** in total sleep time compared to placebo **(p<0.05)**;<br>1 trial found no significant change between groups
		Trazodone: 39<br>No comparator (pre- post-intervention)	5	polysomnography	Mixed results: 2 trials found **significant increase** in total sleep time compared to baseline **(p<0.05)**;<br>3 trials found no significant change from baseline
Vande Griend, 2012<br>Systematic Review	Critically Low	Trazodone: NR<br>Placebo: NR	2	sleep diaries<br>polysomnography	No significant differences between groups
Mayers, 2005<br>Systematic Review	Critically Low	Trazodone<br>Zolpidem<br>Total sample: 306	1	NR	No significant differences found between zolpidem and trazodone
Vande Griend, 2012<br>Systematic Review	Critically Low	Trazodone<br>Zolpidem<br>Total sample: 306	1	daily questionnaire	No significant differences found between zolpidem and trazodone
Wake after sleep onset					
Sateia, 2017<br>Meta-analysis	Critically Low	Doxepin (3mg): 282<br>Placebo: 276	4	polysomnography	Mean difference [95% CI]:<br>**-22.17 [-29.62, -14.72]** I-squared: 23%
		Doxepin (6mg): 209<br>Placebo: 206	3	polysomnography	Mean difference [95% CI]:<br>**-23.4 [-30.34, -16.46]** I-squared: 0%
		Doxepin (6mg): 204<br>Placebo: 197	2	Subjective measure	Mean difference [95% CI]:<br>**-14.39 [-24.86, -3.93]**<br>I-squared: 0%
Yuan, 2010<br>Meta-analysis	Low	Doxepin (1mg): 140<br>Placebo: 139	2	NR	Mean difference [95% CI]:<br>-3.57 [-7.46, 0.32] I-squared: NR
		Doxepin (25mg): 30<br>Placebo: 30	2	NR	Mean difference [95% CI]:<br>**-10.23 [-14.82, -5.64]** I-squared: NR
		Doxepin (3mg): 140<br>Placebo: 139	2	NR	Mean difference [95% CI]:<br>**-5.71 [-9.39, -2.02]** I-squared: NR
		Doxepin (6mg): 141<br>Placebo: 139	2	NR	Mean difference [95% CI]:<br>**-7.36 [-10.69, -4.03]** I-squared: NR

Vande Griend, 2012<br>Systematic Review	Critically Low	Doxepin: NR<br>Placebo: NR	7	polysomnography<br>questionnaire<br>(Likert-type scale)	Doxepin reduced WASO by 5-20 minutes across the trials; 6 out of 7 trials found a **significant difference** compared to placebo **(p<0.05)**
Yeung, 2015<br>Systematic Review	Moderate	Doxepin: NR<br>Placebo: NR	NR	self-report	Adults <65y: mixed results for 3mg and 6mg doxepin over 1-2 nights, 3mg and 6mg doxepin showed positive results at 4 weeks<br>Adults >65y: positive results for 3mg doxepin over 1-2 nights, negative results for 6mg doxepin over 1-2 nights and at 4 weeks
			NR	polysomnography	Adults <65y: multiple trials with positive results for 3mg and 6mg doxepin over 1-2 nights, results were maintained at 4 weeks<br>Adults >65y: positive results for 3mg doxepin over 1-2 nights and at 4 weeks; positive results for 6mg doxepin over 1-2 nights, no data at 4 weeks
Kolla, 2011<br>Systematic Review	Critically Low	Trazodone<br>Placebo<br>Total sample: 16	1	polysomnography	Improved WASO was observed in trazodone participants compared to placebo
Mayers, 2005<br>Systematic Review	Critically Low	Trazodone<br>Placebo<br>Total sample: 306	1	NR	**Significant improvement** in WASO compared to placebo **(p=0.04)**
Mendelson, 2005<br>Systematic review	Critically Low	Trazodone<br>Placebo; unspecified control<br>Total sample: 306	1	self-reported	Relative to placebo, patients reported **significant improvement** during week 1 **(p<0.02)**; during week 2, the trazodone group did not differ significantly from the placebo group.
		Trazodone: 15<br>No comparator (pre- post-intervention)	2	polysomnography	Mixed result:<br>1 trial found **significant improvement (p<0.05)**;<br>1 trial found no significant change from baseline
Vande Griend, 2012<br>Systematic Review	Critically Low	Trazodone: NR<br>Placebo: NR	2	polysomnography	Mixed result:<br>1 trial found a **significant difference** compared to placebo **(p<0.05)**,<br>1 trial found no difference between groups
Mayers, 2005<br>Systematic Review	Critically Low	Trazodone<br>Zolpidem<br>Total sample: 306	1	NR	No significant differences found between zolpidem and trazodone
Vande Griend, 2012<br>Systematic Review	Critically Low	Trazodone<br>Zolpidem<br>Total sample: 306	1	daily questionnaire	No significant differences found between zolpidem and trazodone
Sleep Quality					

Sateia, 2017<br>Meta-analysis	Critically Low	Doxepin (3mg): 148<br>Placebo: 143	2	subjective measure	Standardized mean difference [95% CI]:<br>**0.57 [0.26 to 0.88]** I-squared: 43%
		Doxepin (6mg): 204<br>Placebo: 200	2	subjective measure	Standardized mean difference [95% CI]:<br>**0.28 [0.06 to 0.49]** I-squared: 15%
Mayers, 2005<br>Systematic Review	Critically Low	Doxepin<br>Placebo<br>Total sample: 57	2	NR	Doxepin **significantly improved** sleep quality relative to placebo **(p<0.001)**
Kolla, 2011<br>Systematic Review	Critically Low	Trazodone<br>placebo<br>Total sample: 173	1	Pittsburgh Sleep Quality Index	Sleep quality **improved significantly** in trazodone group during active treatment phase
Mayers, 2005<br>Systematic Review	Critically Low	Trazodone<br>Placebo<br>Total sample: 323	2	NR; Pittsburgh Sleep Quality Index	Sleep quality **significantly improved** compared to placebo in one trial **(p=0.003)**, and almost reached significance in the other (p=0.06) but **significantly higher proportion** of patients in this trial showed **improvement** on sleep quality compared to placebo **(0.004)**
Mendelson, 2005<br>Systematic Review	Critically Low	Trazodone<br>placebo; unspecified control<br>Total sample: 767	5	self-reported (1); Pittsburgh Sleep Quality Index (1); Leeds sleep evaluation questionnaire (3)	Relative to placebo, patients reported **significant improvement** during week 1 **(p<0.02)**;<br>during week 2, the trazodone group did not differ significantly from the placebo group<br>3 trials reported **significant improvements** throughout the 6-week treatment period for "quality of sleep."**(p<0.001)**; Pittsburgh Sleep Quality Index improvements were similar for the placebo and trazodone groups
		Trazodone: 9<br>No comparator (pre- post-intervention)	1	visual analog scale	In the subjective ratings, sleep quality **improved significantly** during weeks 1 and 2 **(p<0.001)** but not during week 3
Mayers, 2005<br>Systematic Review	Critically Low	Trazodone<br>Zolpidem<br>Total sample: 306	1	NR	No significant differences found between zolpidem and trazodone
Sleep Efficiency					
Sateia, 2017<br>Meta-analysis	Critically Low	Doxepin (3mg): 214<br>Placebo: 209	3	polysomnography	Mean difference [95% CI]:<br>**6.78 [4.50 to 9.07]** I-squared: 17%
		Doxepin (6mg): 141<br>Placebo: 139	2	polysomnography	Mean difference [95% CI]:<br>**7.06 [5.12 to 9.01]** I-squared: 0%
Yuan, 2010<br>Meta-analysis	Low	Doxepin (1mg): 140<br>Placebo: 139	2	NR	Mean difference [95% CI]:<br>**3.59 [1.55 to 5.63]** I-squared: NR
		Doxepin (25mg): 30<br>Placebo: 30	2	NR	Mean difference [95% CI]:<br>**12.58 [7.60, 17.56]** I-squared: NR

		Doxepin (3mg): 140<br>Placebo: 139	2	NR	Mean difference [95% CI]:<br>**5.82 [3.75 to 7.90]** I-squared: NR
		Doxepin (6mg): 141<br>Placebo: 139	2	NR	Mean difference [95% CI]:<br>**7.07 [5.12 to 9.01]** I-squared: NR
Mayers, 2005<br>Systematic Review	Critically Low	Doxepin<br>Placebo<br>Total sample: 47	1	NR	Doxepin **significantly increased** sleep efficiency compared to placebo **(p<0.05)**
Vande Griend, 2012<br>Systematic Review	Critically Low	Doxepin: NR<br>Placebo: NR	6	polysomnography<br>questionnaire<br>(7-point Likert type scale)	Doxepin increased SE by 6-10% across the trials; **significant differences** were found compared to placebo **(p<0.05)**
Yeung, 2015<br>Systematic Review	Moderate	Doxepin: NR<br>Placebo: NR	1	polysomnography	Sleep efficiency **significantly improved** compared to placebo after 28 nights (p-value not reported)
Mendelson, 2005<br>Systematic Review	Critically Low	Trazodone<br>placebo; unspecified control<br>Total sample: 56	3	polysomnography	Mixed result:<br>2 trials found **significant improvements** in sleep efficiency compared to placebo **(p<0.05)**;<br>1 trial found no significant change compared to placebo
		Trazodone: 20<br>No comparator (pre- post-intervention)	3	polysomnography	Mixed result:<br>1 trial found **significant improvements** in sleep efficiency compared to baseline **(p<0.05)**;<br>2 trials found no significant change from baseline
Vande Griend, 2012<br>Systematic Review	Critically Low	Trazodone: NR<br>Placebo: NR	2	sleep diaries;<br>polysomnography	Mixed result:<br>1 study found **significant difference** between groups (**p<0.05**, polysomnography data only); 1 study found no difference between group (polysomnography and sleep diary data)
Insomnia Severity Index					
Brasure, 2015<br>Meta-analysis	High	Doxepin: 289<br>Placebo: 205	2	ISI	Mean change [95% CI]:<br>**-1.74 [-2.59 to -0.88]** I-squared: 0%

Table E5. Anti-psychotics

Author, Year<br>Synthesis type	AMSTAR rating	Intervention and sample size	# of studies	Measurement method	Pooled Estimates (MA) or<br>Narrative Results (from SR with no MA)
Sleep onset latency					
Anderson, 2014<br>Systematic Review	Critically Low	Quetiapine<br>No comparator (pre- post-intervention)<br>Total sample: 70	2	polysomnography;<br>Spiegel sleep questionnaire	Change in outcome (p-value):<br>24.2 +/-19.0 minutes (p=NS);<br>Improvement in Spiegel sleep questionnaire score (p-value not reported)
		Quetiapine<br>Placebo; no therapy<br>Total sample: 52	2	polysomnography or actigraphy	Change in outcome (p-value):<br>66.5+/-51.2 v 47.4+/-30.4 minutes (p=NS);<br>**15.6+/-18.1 minutes v 24.5+/-30.2 minutes (p<0.05)**
Coe, 2014<br>Systematic Review	Critically Low	Quetiapine: 8<br>Placebo: 8	1	patient-recorded sleep logs	Change in outcome (p-value):<br>-96.16 v -23.72 minutes (p=0.07)
Wine, 2009<br>Systematic Review	Critically Low	Quetiapine: 32<br>No comparator (pre- post-intervention)	2	NR	Change in outcome (p-value):<br>22±17 v 24±19 minutes (p=NS);<br>**82±65 v 29±23 minutes (p<0.05)**<br>Significant reduction in sleep latency scores compared to baseline with quetiapine use
Total Sleep Time					
Anderson, 2014<br>Systematic Review	Critically Low	Quetiapine: 18<br>No comparator (pre- post-intervention)	1	polysomnography	Change in outcome:<br>**395.6+/-62.3 (p<0.05 compared to baseline)**
		Quetiapine<br>Placebo; no therapy<br>Total sample: 52	2	polysomnography actigraphy	Change in outcome:<br>347.5+/-100.9 v 361.9+/-85.4 (p=NS);<br>432 +/-66 v 390+/-54 (p=NS)
Coe, 2014<br>Systematic Review	Critically Low	Quetiapine: 8<br>Placebo: 8	1	patient-recorded sleep logs	Change in outcome (p-value):<br>+124.92 v +72.24 minutes (p=0.193)
		Quetiapine: 18<br>No comparator (pre- post-intervention)	1	objective; from baseline to week 6	TST **significantly improved** from baseline compared to week 6 **(p=0.03)**
Wine, 2009<br>Systematic Review	Critically Low	Quetiapine: 18<br>No comparator (pre- post-intervention)	1	NR	Change in outcome (p-value):<br>**396±62 to 358±61mins (p<0.05)**<br>**Significant decrease** in TST vs baseline for quetiapine
		Quetiapine<br>Untreated control<br>(pre- post- intervention)<br><br>Total sample: 18	1	NR	Change in outcome (p-value):<br>**[pre-post] 240±60 to 360±120 min (p<0.05)**;<br>Quetiapine **improved** total sleep time compared to control
Sleep Quality					

Anderson, 2014<br>Systematic Review	Critically Low	Quetiapine: 84<br>No comparator (pre- post-intervention)	3	PSQI; Spiegel Sleep Questionnaire	**Significant improvements** on PSQI global scores **(p<0.001)**; 75% improvement in global score for SSQ (p-value not reported)
		Quetiapine Placebo; no therapy<br>Total sample: 78	3	PSQI; actigraphy	**Significant improvement** in PSQI scores from baseline and compared to placebo **(p<0.001)**
Coe, 2014<br>Systematic Review	Critically Low	Quetiapine: 18<br>No comparison	1	PSQI; sleep diary	Subjective parameters such as PSQI and sleep diaries also showed **significant improvement** from baseline to weeks 2 and 6
Kolla, 2011<br>Systematic Review	Critically Low	Quetiapine: 28<br>No comparator (pre- post-intervention)	1	HAM-D's sleep question subset	Middle and late insomnia was **significantly reduced** at 2 weeks, no other sleep data presented
Wine, 2009<br>Systematic Review	Critically Low	Quetiapine: 18<br>No comparator (pre- post-intervention	1	PSQI	PSQI decreased total scores with use of quetiapine vs baseline
Sleep Satisfaction					
Anderson, 2014<br>Systematic Review	Critically Low	Quetiapine Placebo; no therapy<br>Total sample: 25	1	visual analogue scale	Non-significant improvement based on visual analog scale (p=0.505)
Sleep Efficiency					
Anderson, 2014<br>Systematic Review	Critically Low	Quetiapine: 18<br>No comparator (pre- post-intervention)	1	polysomnography	Change in outcome (p-value):<br>**89.9+/-8.2 (p<0.05)**
		Quetiapine Placebo; no therapy<br>Total sample: 27	1	polysomnography or actigraphy	Change in outcome (p-value):<br>**82.7+/-9.1 v 77.0+/-7.9 (p<0.05)**
Coe, 2014<br>Systematic Review	Critically Low	Quetiapine: 18<br>No comparator (pre- post-intervention)	1	Objective measure	Sleep efficiency **significantly improved** from baseline compared to week 6 **(p=0.02)**
Wine, 2009<br>Systematic Review	Critically Low	Quetiapine: 18<br>No comparator (pre- post-intervention)	1	NR	Change in outcome (p-value):<br>**83%±14%→90%±8% (p<0.05)**<br>**Significant increase** in sleep efficiency with quetiapine use compared to baseline
Insomnia Severity Index					
Anderson, 2014<br>Systematic Review	Critically Low	Quetiapine: 6<br>No comparator (pre- post-intervention)	1	ISI	In 5 of 6 patients, the ISI score moved from moderate insomnia to absence of insomnia at week 1 and was maintained
Hangover/Morning Sedation					

Anderson, 2014<br>Systematic Review	Critically Low	Quetiapine Placebo; no therapy	2	NR	Daytime sedation was **significantly more common** in the quetiapine group<br>(compared to placebo, p-value not reported)

Table E6. Melatonin

**Author, Year**<br>**Synthesis type**	**AMSTAR rating**	**Intervention and sample size**	**# of studies**	**Measurement method**	**Pooled Estimates (MA) or**<br>**Narrative Results (from SR with no MA)**
Sleep onset latency					
Buscemi, 2005<br>Meta-analysis	High	Melatonin: 103<br>Placebo: 103	8	sleep diary<br>polysomnography	Mean difference [95% CI]:<br>**-8.25 [-14.45, -2.04]** I-squared: 44.2%
Buscemi, 2004<br>Meta-analysis	High	Melatonin: 178<br>Placebo: 167	12	NR	Mean difference [95% CI]:<br>**-10.66 [-17.61, -3.72]** I-squared: 81.5%
Ferracioli-Oda, 2013<br>Meta-analysis	Critically Low	Melatonin: NR<br>Placebo: NR	8	polysomnography or actigraphy	Mean difference [95% CI]:<br>**5.5 [2.29, 7.81]** I-squared: NR
Lee, NA<br>Meta-analysis	Critically Low	Melatonin: NR<br>Placebo: NR	12	sleep diary<br>polysomnography or actigraphy	Mean difference [95% CI]:<br>**-3.71 [-6.78, -0.63]** I-squared: 39%
Bellon, 2006<br>Systematic Review	Critically Low	Melatonin: NR<br>Placebo: NR	13	subjective<br>polysomnography<br>actigraphy	Adults: 3 studies **significant improvement (p<0.05);** 1 study non-significant improvement; 1 study improved subjectively and non-significant change on polysomnography<br>Elderly: 2 studies non-significant improvement; 2 studies no change; 1 study **significant improvement (P<0.05)**<br>Schizophrenia, Dementia, and medically ill patients: 3 studies, non-significant improvement
Culpepper, 2015<br>Systematic Review	Critically Low	Melatonin<br>Placebo<br>Total sample: 772	3	polysomnography<br>actigraphy sleep diary	No significant difference between groups (melatonin vs placebo)
Vural, 2014<br>Systematic Review	Critically Low	Melatonin<br>Control<br>Total sample: 14	1	NR	**Significant decrease** in sleep latency with morning and nighttime melatonin doses; **significant decrease** in sleep latency to 10mins of persistent sleep with early and continuous melatonin doses
Total Sleep Time					
Buscemi, 2004<br>Meta-analysis	High	Melatonin: NR<br>Placebo: NR	11	NR	Mean difference [95% CI]:<br>4 [-10.5, 18.5] I-squared: 67.6%
Ferracioli-Oda, 2013<br>Meta-analysis	Critically Low	Melatonin: NR<br>Placebo: NR	10	polysomnography<br>actigraphy	Mean change [95% CI]:<br>0.34 [-11.19, 11.87] I-squared: NR

Lee, NA<br>Meta-analysis	Critically Low	Melatonin: NR<br>Placebo: NR	11	sleep diary<br>polysomnography<br>actigraphy	Mean difference [95% CI]:<br>**3.3 [7.04, 13.65]** I-squared:12%
McCleery, 2016<br>Meta-analysis	High	Melatonin: 119<br>Placebo: 65	2	actigraphy	Mean difference [95% CI]:<br>10.68 [-16.22, 37.59] I-squared: 0%
Xu, 2015<br>Meta-analysis	Moderate	Melatonin: 257<br>Placebo; light therapy: 240	8	actigraphy	Mean difference [95% CI]:<br>**24.36 [3.26, 45.46]** I-squared: 59%
Zhang, 2016<br>Meta-analysis	Critically Low	Melatonin: 101<br>Placebo: 96	4	polysomnography<br>actigraphy	Mean difference [95% CI]:<br>12.38 [-10.38, 35.15] I-squared: 34%
Bellon, 2006<br>Systematic Review	Critically Low	Melatonin: NR<br>Placebo: NR	15	subjective<br>polysomnography<br>actigraphy	Adult patients: 2 studies report no change; 2 report decrease; 2 report **significant improvement**; 1 reports subjective improvement but no change on PSG<br>Elderly patients: 4 studies report no change<br>Schizophrenia, dementia, Alzheimer's patients: 3 studies, non-significant improvement<br>Medically ill patients: 1 study, **significant improvement**
Chase, 1997<br>Systematic Review	Critically Low	Melatonin<br>Placebo<br>Total sample: 25	2	wrist actigraphy; subjective report	1 study found no change in total sleep time; 1 study found Melatonin treatment resulted in a **significant effect** on reported time asleep
Costello, 2014<br>Systematic Review	Moderate	Melatonin<br>Placebo<br>Total sample: 791	1	National sleep foundation diary; Pittsburgh Sleep Quality Index	Melatonin (Circadin) **significantly increased** sleep time compared to placebo **(p=0.035)**
Culpepper, 2015<br>Systematic Review	Critically Low	Melatonin<br>Placebo<br>Total sample: 50	2	sleep diary<br>polysomnography<br>actigraphy	Change in outcome (p-value):<br>**-15.4 vs -5.5 minutes (p<0.01)** [1 study]<br>1 study: No significant difference between groups (melatonin vs placebo);<br>1 study: **Significant reduction** in sleep latency **(p<0.01)**
Wake after sleep onset					
Buscemi, 2005<br>Meta-analysis	High	Melatonin: 68<br>Placebo: 68	5	sleep diary,<br>polysomnography	Mean difference [95% CI]:<br>-9.65 [-33.57, 14.26] I-squared: 89.8%
Buscemi, 2004<br>Meta-analysis	High	Melatonin: NR<br>Placebo: NR	5	NR	Mean difference [95% CI]:<br>-1.4 [-21.8, 19] I-squared: 84%
		Melatonin: NR<br>Placebo: NR	3	NR	Mean difference [95% CI]:<br>-6.3 [-16.6, 3.9] I-squared: 35.3%
Zhang, 2016<br>Meta-analysis	Critically Low	Melatonin: 75<br>Placebo: 69	2	polysomnography<br>actigraphy	Mean difference [95% CI]:<br>10.93 [-6.07, 27.92] I-squared: 0%

Chase, 1997<br>Systematic Review	Critically Low	Melatonin<br>Placebo<br>Total sample: 12	1	wrist actigraphy	Change in outcome (p-value):<br>**49 minutes v 73 minutes (p<0.001)**
Vural, 2014<br>Systematic Review	Critically Low	Melatonin<br>Control<br>Total sample: 12	1	NR	**Significant decrease** in wake after sleep onset in melatonin group
Sleep Quality					
Buscemi, 2004<br>Meta-analysis	High	Melatonin: NR<br>Placebo: NR	2	NR	Standardized mean difference [95% CI]:<br>0.5 [-0.1, 1.1] I-squared: 0%
Ferracioli-Oda, 2013<br>Meta-analysis	Critically Low	Melatonin: NR<br>Placebo: NR	14	polysomnography<br>actigraphy<br>sleep scales questionnaires<br>sleep logs	Mean change [95% CI]:<br>**0.22 [0.13, 0.32]** I-squared: 0%
McCleery, 2016<br>Meta-analysis	High	Melatonin: 111<br>Lactose placebo: 53	2	carer-rated sleep quality	Standardized mean difference [95% CI]:<br>0.04 [-0.29, 0.38] I-squared: 46%
Lee, NA<br>Meta-analysis	Critically Low	Melatonin: 675<br>Placebo: 672	10	Leeds Sleep Evaluation Questionnaire – Quality of Sleep; The Pittsburgh Sleep Quality Index Component 1; The Northside Hospital Sleep Medicine Institute Test; Daily Sleep Questionnaire	Standardized mean difference [95% CI]:<br>0.16 [-0.06, 0.39] I-squared: 58%
Sateia, 2017<br>Meta-analysis	Critically Low	Melatonin: 233<br>Placebo: 228	3	subjective measure	Standardized mean difference [95% CI]:<br>0.21 [-0.36, 0.77] I-squared: 83%
Bellon, 2006<br>Systematic Review	Critically Low	Melatonin: NR<br>Placebo: NR	11	subjective<br>polysomnography<br>actigraphy	Adult patients: 4 studies showed **significant improvement**; 2 studies no change<br>Elderly patients: 2 studies no change<br>Medically ill patients: 1 study **significant improvement**<br>Dementia or Alzheimer's patients: 2 studies, no change
Chase, 1997<br>Systematic Review	Critically Low	Melatonin<br>Placebo<br>Total sample: 10	1	daily sleep questionnaire	No statistical difference between groups noted, except patients in melatonin group had **significantly improved** perceived quality of sleep **(p<0.03)**

Costello, 2014<br>Systematic Review	Moderate	Melatonin<br>Placebo<br>Total sample: 54	3	sleep diary; subjective sleep quality questionnaire; Daily Sleep Questionnaire; Stanford Sleepiness Scale; Visual Analog Scale	Melatonin did not affect sleep quality in patients with primary insomnia; melatonin **significantly improved** sleep quality compared to placebo, indicating that controlled-release melatonin may effectively facilitate discontinuation of benzodiazepine therapy while maintaining good sleep quality; melatonin 5 mg resulted in an improvement in overall subjective sleep quality **(p=0.03)** compared to 1 mg and placebo.
Culpepper, 2015<br>Systematic Review	Critically Low	Melatonin<br>Placebo<br>Total sample: 48	2	sleep questionnaire: visual analogue scale; 38-item Northside Hospital Sleep Medicine Institute test	Change in outcome (p-value):<br>**1.78 vs 3.44 (p<0.05)**<br>1 study: No significant difference between groups (melatonin vs placebo);<br>1 study: **Significantly improved** sleep quality
		Melatonin<br>Placebo<br>Total sample: 344	2	polysomnography sleep diary (2)	Change in outcome (p-value):<br>**26% v 15% (p<0.05)**<br>1 study: no significant difference between groups;<br>1 study: **Higher rate** of sleep quality in patients while on melatonin vs placebo
Vural, 2014<br>Systematic Review	Critically Low	Melatonin<br>Control<br>Total sample: 27	1	NR	**Significant increase** in sleep quality in melatonin group
Sleep Satisfaction					
Vural, 2014<br>Systematic Review	Critically Low	Melatonin<br>Control<br>Total sample: 112	1	% nights scored good;<br>% good mood	**Significant increase** in % nights scored good and significant increase in % good mood in melatonin group
Sleep Efficiency					
Buscemi, 2004<br>Meta-analysis	High	Melatonin:117<br>Placebo: 117	9	NR	Mean difference [95% CI]:<br>1.45 [-0.66, 3.56] I-squared: 62.8%
McCleery, 2016<br>Meta-analysis	High	Melatonin: 104<br>Placebo: 47	1	Actigraphy	Mean difference [95% CI]:<br>-0.01 [-0.04, 0.03] I-squared: 0%
Xu, 2015<br>Meta-analysis	Moderate	Melatonin: 232<br>Placebo; light therapy: 214	6	Actigraphy	Mean difference [95% CI]:<br>1.78 [-0.13, 3.70] I-squared: 25%
Lee, NA<br>Meta-analysis	Critically Low	Melatonin: 123<br>Placebo: 125	8	sleep diary<br>polysomnography<br>actigraphy	Mean difference [95% CI]:<br>**2.74 [0.41, 5.88]** I-squared: 54%
Zhang, 2016<br>Meta-analysis	Critically Low	Melatonin: 75<br>Placebo: 69	2	polysomnography<br>actigraphy	Mean difference [95% CI]:<br>-0.01 [-0.04, 0.02] I-squared: 0%

Bellon, 2006<br>Systematic Review	Critically Low	Melatonin: NR<br>Placebo: NR	12	subjective<br>polysomnography<br>actigraphy	Adult patients: No change across all studies<br>Elderly patients: 2 studies no change; 2 studies **significant improvement**<br>Schizophrenia patients: 1 study **significant improvement**<br>Dementia or Alzheimer's: 2 studies, no change
Chase, 1997<br>Systematic Review	Critically Low	Melatonin<br>Placebo<br>Total sample: 35	2	wrist actigraphy	Proportion of respondents (p-value):<br>**83% v 75% (p<0.001)**<br>Increase in sleep efficiency; A **significant difference** in sleep efficiency was noted in the elderly without sleep disorders and compared with those with insomnia **(p<0.0001)**
Culpepper, 2015<br>Systematic Review	Critically Low	Melatonin<br>Placebo<br>Total sample: 40	1	Actigraphy	No significant difference between groups
Health-related Quality of Life					
Vural, 2014<br>Systematic Review	Critically Low	Melatonin<br>Control<br>Total sample: 42	1	NR	**Significant increase** in quality of life in melatonin group

Table E7. Diphenhydramine

Author, Year<br>Synthesis type	AMSTAR rating	Intervention and sample size	# of studies	Measurement method	Pooled Estimates (MA) or<br>Narrative Results (from SR with no MA)
Sleep onset latency					
Sateia, 2017<br>Meta-analysis	Critically Low	Diphenhydramine: 79 patients<br>Placebo: 84 patients	2	subjective measure	Mean difference [95% CI]:<br>-2.47 [-8.17, 3.23] I-squared: 0%
Culpepper, 2015<br>Systematic Review	Critically Low	Diphenhydramine<br>Placebo<br>Total sample: 226	3	sleep diary	Change in outcome (p-value):<br>34.2 vs 36.8 mins (p=NS);<br>21.6 vs 23.8 mins (p=NS);<br>**138.5 vs 99.9 mins (p<0.05)**
Vande Griend, 2012<br>Systematic Review	Critically Low	Diphenhydramine<br>Placebo<br>Total sample: 332	4	sleep diaries<br>questionnaire<br>polysomnography	Overall, the outcomes analyzed from all four trials provided mixed results, with the majority not being statistically different than placebo (P > 0.05); 3 studies found no difference compared to placebo and 2 studies found the drug was **superior to placebo**
Total Sleep Time					
Sateia, 2017<br>Meta-analysis	Critically Low	Diphenhydramine: 77 patients<br>Placebo: 84 patients	2	subjective measure	Mean difference [95% CI]:<br>17.86 [-3.79, 39.51] I-squared: 0%

Culpepper, 2015<br>Systematic Review	Critically Low	Diphenhydramine<br>Placebo<br>Total Sample: 204	2	sleep diary	Change in outcome (p-value):<br>6.6 vs 6.3 minutes (p=NS);<br>No change (p=NS);<br>No significant changes in TST compared to placebo
Vande Griend, 2012<br>Systematic Review	Critically Low	Diphenhydramine<br>Placebo<br>Total sample: 332	4	sleep diaries<br>polysomnography<br>questionnaire (unspecified)	Overall, the outcomes analyzed from all four trials provided mixed results, with the majority not being statistically different than placebo (P > 0.05);<br>4 studies found no significant difference between groups; 1 study found drug to be **superior**
Wake after sleep onset					
Vande Griend, 2012<br>Systematic Review	Critically Low	Diphenhydramine<br>Placebo<br>Total sample: 17	1	questionnaire (unspecified)	Overall, the outcomes analyzed from all four trials provided mixed results, with the majority not being statistically different than placebo (p>0.05)
Sleep quality					
Culpepper, 2015<br>Systematic Review	Critically Low	Diphenhydramine<br>Placebo<br>Total Sample: 20	1	sleep diary	Change in outcome (p-value):<br>No difference in subjective scores<br>3.0 vs 2.9 (p=NS)<br>No significant treatment difference for sleep quality
Sleep efficiency					
Vande Griend, 2012<br>Systematic Review	Critically Low	Diphenhydramine<br>Placebo<br>Total Sample: 204	1	sleep diaries<br>polysomnography	Overall, the outcomes analyzed from all four trials provided mixed results, with the majority not being statistically different than placebo (p>0.05);<br>Sleep diary data showed **significant improvement (p<0.05)**;<br>polysomnography data showed no difference compared to placebo
Insomnia Severity Index					
Culpepper, 2015<br>Systematic Review	Critically Low	Diphenhydramine<br>Placebo<br>Total sample: 184	1	ISI	Change in outcome (p=value):<br>**9.39 vs 11.63 (p<0.01)**<br>**Significantly lower** ISI with diphenhydramine after 2 weeks

Table E8. Cognitive Behavioural Interventions

**Author, Year Synthesis type**	**AMSTAR rating**	**Intervention and sample size**	**# of studies**	**Measurement method**	**Pooled Estimates (MA) or Narrative Results (from SR with no MA)**
Sleep onset latency					
Brasure, 2015 Meta-analysis	High	CBT-I: 626<br><br>sham treatment/ placebo, wait-list control, no treatment, or sleep hygiene/sleep education: 620	15	subjective report	Mean difference [95% CI]: **-12.7 [-18.23, -7.18]** I-squared: 78%
		CBT-I: 108 [older adults]<br><br>placebo, wait-list control, no treatment, or sleep hygiene/sleep education: 83	3	subjective report	Mean difference [95% CI]: **-9.98 [-16.48, -3.48]** I-squared: 0%
		CBT-I: 61 [adults with chronic pain]<br><br>passive control (placebo or sham treatment or wait-list): 61	3	subjective report	Mean difference [95% CI]: **-26.5 [-43.25, -9.75]** I-squared: 77%
Cheng, 2012 Meta-analysis	Low	CBT - sleep hygiene; stimulus control; relaxation training; sleep restriction; cognitive restructuring: NR<br><br>wait list control: NR	4	sleep diary	Standardized mean difference [95% CI]: **-0.55 [-0.80, -0.30]** I-squared: 0%
Ho, 2016 Meta-analysis	Low	CBT- image rehearsal therapy; exposure, re-scripting and relaxation therapy; mind-body bridging; behavioural sleep intervention: NR<br><br>wait list control; sleep hygiene: NR	4	sleep diary	Standardized mean difference [95% CI]: **-0.83 [-1.19, -0.47]** I-squared: 0%
Irwin, 2006 Meta-analysis	Critically Low	CBT – relaxation, biofeedback, hypnosis, sleep compression/ restriction, paradoxical intention: NR<br><br>control: NR	21	self-report	Cohen's *d* [95% CI]: **-0.52 [-0.68 to -0.82]** Q-statistic: 74.66

Author, Year<br>Synthesis type	AMSTAR rating	Intervention and sample size	# of studies	Measurement method	Pooled Estimates (MA) or<br>Narrative Results (from SR with no MA)
Johnson, 2016<br>Meta-analysis	Moderate	CBT-I with both cognitive and behavioral components: 423<br><br>waitlist control; treatment-as-usual; sleep education; behavioral placebo; mindfulness-based stress reduction: 297	8	sleep diary	Cohen's *d* [95% CI]:<br>**0.27 [0.11 to 0.44]** I-squared: 0.2%
Koffel, 2015<br>Meta-analysis	Critically Low	group CBT-I - stimulus control, sleep restriction, and addressing dysfunctional beliefs about sleep: NR<br><br>wait list, treatment as usual, placebo: NR	6	sleep diary	Mean effect size [95% CI]:<br>**0.47 [0.27; 0.66]** I-squared: NR
Montgomery, 2003<br>Meta-analysis	High	CBT - sleep hygiene; stimulus control; muscle relaxation; sleep restriction; cognitive therapy; education; imagery training: 86<br><br>wait-list control, placebo: 49	3	sleep diary	Mean difference [95% CI]:<br>**-3 [-8.92, 2.92]** I-squared: 0%
Navarro-Bravo, 2015<br>Meta-analysis	Moderate	CBT - sleep restriction, stimulus control, sleep education/hygiene: NR<br><br>Placebo; wait list control; stress management and wellness training; treatment as usual; sleep hygiene/education: NR	7	sleep diary; actigraphy; sleep evaluation (4-item questionnaire);	Cohen's *d* [95% CI]:<br>**-0.46 [-0.76, -0.15]** Chi square: 19.88; 0.003
Seyffert, 2016<br>Meta-analysis	Low	Internet-based CBT - sleep education, stimulus control, sleep restriction, relaxation, sleep hygiene, cognitive techniques: NR<br><br>Wait-list control, Internet control, treatment as usual: NR	7	sleep diary	Mean difference [95% CI]:<br>**-10.68 [-16.00, -5.37]** I-squared: 4.3%

**Author, Year**<br>**Synthesis type**	**AMSTAR rating**	**Intervention and sample size**	**# of studies**	**Measurement method**	**Pooled Estimates (MA) or**<br>**Narrative Results (from SR with no MA)**
van Straten, 2007<br>Meta-analysis	Low	CBT - relaxation; sleep restriction; stimulus control; paradoxical intention; identifying and challenging dysfunctional thoughts: NR<br><br>waitlist control; no treatment; placebo; psycho-education: NR	108	sleep diary	Hedges *g* [95% CI]:<br>**0.57 [0.50, 0.65]** I-squared: 48%
van Straten, 2009<br>Meta-analysis	Moderate	CBT (self-help): stimulus-control; sleep restriction; cognitive therapy; sleep hygiene; relaxation; in-bed exercises: NR<br><br>waiting list: NR	8	sleep diary	Cohen's *d* [95% CI]:<br>**0.29 [0.15, 0.43]** I-squared: 0%
Ye, 2016<br>Meta-analysis	Moderate	CBT - sleep hygiene education; cognitive restructuring; stimulus control; sleep restriction; relaxation therapy; hierarchy development; imagery training; scheduled pseudo desensitization; breathing control: 1006<br><br>wait-list control, treatment as usual; internet + email; internet + telephone; telephone; internet-based control: 1004	15	NR	Mean difference [95% CI]:<br>**-18.41 [-23.21, 13.60]** I-squared: 62%
Brooks, 2014<br>Systematic Review	Low	CBT-I (unspecified)<br><br>Control (unspecified)<br><br>Total sample: 60	1	daily sleep diary; Pittsburgh Sleep Quality Index; actigraphy	Improved self-reported sleep latency maintained for 6 months post-treatment; not corroborated by actigraphy
Ishak, 2012<br>Systematic Review	Low	CBT (unspecified)<br><br>Placebo; no treatment; usual care<br><br>Total sample: 209	1	SF-36	**Significant reductions** in sleep latency for CBT compared to placebo **(p<0.01)**

Author, Year<br>Synthesis type	AMSTAR rating	Intervention and sample size	# of studies	Measurement method	Pooled Estimates (MA) or<br>Narrative Results (from SR with no MA)
Dickerson, 2014<br>Systematic Review	High	CBT (unspecified):<br><br>Control: usual treatment; waitlist crossover; waitlist control; control; usual treatment<br><br>Total sample: 150	1	actigraphy	Mean change [95% CI]:<br>**-0.42 [-0.80 to -0.01]**<br>CBT moderate effect in decreasing insomnia symptoms
Venables, 2014<br>Systematic Review	Critically Low	group CBT: 660<br><br>No comparator (pre- post-intervention)	8	NR	The group CBT studies obtained improvements in all sleep parameters, group delivered CBT sessions may be slightly more effective than individual sessions
		professionally administered CBT: 615<br><br>No comparator (pre- post-intervention)	8	actigraphy; sleep diary	7 of 8 studies that used CBT found a reduction in sleep latency (values not reported)
Wang, 2005<br>Systematic Review	Moderate	CBT: stimulus control; sleep restriction; cognitive therapy; sleep hygiene education; sleep scheduling<br><br>control; Quasi desensitization; self-monitoring control; sleep hygiene recommendations; waiting-list control<br><br>Total sample: 109	1	sleep diary; wrist actigraphy	Change in outcome:<br>CBT: 61→28mins; control: 74→70mins<br><br>CBT **significantly improved** sleep latency compared to control with sustained mean reduction by 50% in CBT group **($p<0.05$)**
Ho, 2015<br>Meta-analysis	Low	Multi-component CBT - stimulus control, sleep restriction, sleep hygiene, relaxation and/or cognitive therapy: NR<br><br>waiting-list control; routine care or no treatment: NR	8	sleep diary	Hedges *g* [95% CI]:<br>**-0.70 [-1.0, -0.4]** I-squared: 77%

Author, Year<br>Synthesis type	AMSTAR rating	Intervention and sample size	# of studies	Measurement method	Pooled Estimates (MA) or<br>Narrative Results (from SR with no MA)
Trauer, 2015<br>Meta-analysis	Moderate	Multi-modal CBT - cognitive therapy, stimulus control, sleep restriction, relaxation, sleep hygiene: NR<br><br>Wait-list control, treatment as usual, Sleep hygiene, sham, placebo: NR	16	sleep diary	Mean difference [95% CI]:<br>**-19.03 [-23.93, 14.12]** I-squared: 41.9%
Buscemi, 2005<br>Meta-analysis	High	Multi-component CBT - Paradoxical Intention; Sleep compression; Stimulus control:152<br><br>Placebo: 124	9	sleep diary	Mean difference [95% CI]:<br>**-4.57 [-9.75, 0.61]** I-squared: 12.5%
Okajima, 2011<br>Meta-analysis	Critically Low	Multi-component CBT - sleep hygiene education; sleep restriction; stimulus control; cognitive therapy; relaxation; paradoxical intention: NR<br><br>wait-list control, placebo; sleep hygiene education; control(unspecified); treatment as usual: NR	7	sleep diary	Cohen's *d* [95% CI]:<br>**0.4 [0.21, 0.57]** I-squared: NR
			2	polysomnography; actigraphy	Cohen's *d* [95% CI]:<br>**0.59 [0.08, 1.02]** I-squared: NR
Zachariae, 2016<br>Meta-analysis	Moderate	Multi-component CBT - stimulus control, sleep hygiene; cognitive therapy; sleep restriction; relaxation techniques: NR<br><br>Wait-list control, treatment as usual: NR	10	NR	Hedges *g* [95% CI]:<br>**0.41 [0.29, 0.53]** I-squared: 0%

Author, Year<br>Synthesis type	AMSTAR rating	Intervention and sample size	# of studies	Measurement method	Pooled Estimates (MA) or<br>Narrative Results (from SR with no MA)
Howell, 2014<br>Systematic Review	Moderate	Multi-component CBT - sleep hygiene, sleep restriction, stimulus control, cognitive restructuring, and relaxation therapies<br><br>Usual care; wait-list control, healthy eating and nutrition; sleep education and hygiene only; no treatment<br><br>Total sample: 235	3	sleep diary	Sleep latency had **significant improvement** in all studies post CBT intervention (values not reported)
McCurry, 2007<br>Systematic Review	Critically Low	Multi-component CBT - sleep hygiene, relaxation, sleep compression, cognitive behavioural therapy, stimulus control<br><br>delayed treatment; wait-list control, placebo; stress management<br><br>Total sample: 92	1	sleep logs	CBT **significantly decreased** sleep latency compared to stress management (p-values not reported)
Yang, 2014<br>Meta-analysis	Moderate	CBT + relaxation (sleep hygiene; relaxation CD): 13<br><br>Sleep hygiene education; treatment as usual: 13	1	NR	Standardized mean difference [95% CI]:<br>**1.33 [0.46, 2.19]** I-squared: 0%
Buscemi, 2005<br>Meta-analysis	High	CBT + relaxation: relaxation training; cognitive control; stimulus control; group relaxation; aggressive muscle relaxation; cognitive distraction: 45<br><br>placebo: 46	4	sleep diary	Mean difference [95% CI]:<br>**-21.5 [-42.2, -0.8]** I-squared: 74.4%
van Straten, 2009<br>Meta-analysis	Moderate	CBT (self-help): stimulus-control; sleep restriction; cognitive therapy; sleep hygiene; relaxation; in-bed exercises: NR<br><br>CBT (face-to-face)	3	sleep diary	Cohen's *d* [95% CI]:<br>**-0.37 [-0.73, -0.02]** I-squared: 0%

Author, Year<br>Synthesis type	AMSTAR rating	Intervention and sample size	# of studies	Measurement method	Pooled Estimates (MA) or Narrative Results (from SR with no MA)
Buscemi, 2005<br>Meta-analysis	High	CBT + relaxation - relaxation training; cognitive control; stimulus control; group relaxation: 18<br><br>progressive muscle relaxation; EMG biofeedback; group relaxation: 16	2	sleep diary	Mean difference [95% CI]:<br>-9.2 [-37.9, 19.5] I-squared: 37.1%
		CBT + relaxation - relaxation training; stimulus control; aggressive muscle relaxation; cognitive distraction: 23<br><br>CBT - cognitive therapy; sleep restriction; stimulus control; sleep hygiene: 24	2	sleep diary	Mean difference [95% CI]:<br>-4.6 [-20.7, 11.5] I-squared: 0%
Total Sleep Time					
Brasure, 2015<br>Meta-analysis	High	CBT-I: 621<br><br>sham treatment/ placebo, wait-list control, no treatment, or sleep hygiene/sleep education): 612	15	subjective report	Mean difference [95% CI]:<br>**14.24 [2.08, 26.39]** I-squared: 56%
Cheng, 2012<br>Meta-analysis	Low	CBT - sleep hygiene; stimulus control; relaxation training; sleep restriction; cognitive restructuring: NR<br><br>wait list control: NR	4	sleep diary	Standardized mean difference [95% CI]:<br>0.22 [-0.03, 0.46] I-squared: 0%
Ho, 2016<br>Meta-analysis	Low	CBT - image rehearsal therapy; exposure, re-scripting and relaxation therapy; mind-body bridging; behavioural sleep intervention: NR<br><br>wait list control; sleep hygiene: NR	4	sleep diary	Standardized mean difference [95% CI]:<br>0.39 [-0.05, 0.84] I-squared: 38%
Irwin, 2006<br>Meta-analysis	Critically Low	CBT – relaxation, biofeedback, hypnosis, sleep compression/restriction paradoxical intention: NR<br><br>control: NR	16	self-report	Cohen's *d* [95% CI]:<br>0.17 [-0.13, 0.48] Q-statistic: 50.27

Author, Year<br>Synthesis type	AMSTAR rating	Intervention and sample size	# of studies	Measurement method	Pooled Estimates (MA) or<br>Narrative Results (from SR with no MA)
Koffel, 2015<br>Meta-analysis	Critically Low	group CBT-I - stimulus control, sleep restriction, and addressing dysfunctional beliefs about sleep: NR<br><br>wait list, treatment as usual, placebo: NR	6	sleep diary	Mean effect size [95% CI]:<br>-0.04 [-0.32, 0.23] I-squared: NR
Montgomery, 2003<br>Meta-analysis	High	CBT - sleep hygiene; stimulus control; muscle relaxation; sleep restriction; cognitive therapy; education; imagery training: 76<br><br>wait-list control, placebo: 67	4	Sleep Diary	Mean difference [95% CI]:<br>-14.56 [-36.13, 7.01] I-squared: 0%
		CBT - sleep hygiene; stimulus control; muscle relaxation; sleep restriction; cognitive therapy; education; imagery training: 30<br><br>wait-list control, placebo: 29	2	polysomnography	Mean difference [95% CI]:<br>18.93 [-2.74, 40.60] I-squared: 0%
Navarro-Bravo, 2015<br>Meta-analysis	Moderate	CBT - sleep restriction, stimulus control, sleep education/hygiene: NR<br><br>placebo; wait list control; stress management and wellness training; treatment as usual; sleep hygiene/education: NR	8	sleep diary<br>polysomnography<br>actigraphy<br>sleep evaluation (4-item questionnaire)	Cohen's *d* [95% CI]:<br>0.11 [-0.15, 0.37] Chi square: 17.56; 0.014
Seyffert, 2016<br>Meta-analysis	Low	Internet-based CBT - sleep education, stimulus control, sleep restriction, relaxation, sleep hygiene, cognitive techniques: NR<br><br>Wait-list control, internet control, Treatment as usual: NR	8	sleep diary	Mean difference [95% CI]:<br>**19.57 [8.56, 30.58]** I-squared: 24.7%

Author, Year Synthesis type	AMSTAR rating	Intervention and sample size	# of studies	Measurement method	Pooled Estimates (MA) or Narrative Results (from SR with no MA)
van Straten, 2007 Meta-analysis	Low	CBT - relaxation; sleep restriction; stimulus control; paradoxical intention; identifying and challenging dysfunctional thoughts: NR<br><br>waitlist control; no treatment; placebo; psycho-education: NR	91	sleep diary	Hedges *g* [95% CI]:<br>**0.16 [0.08, 0.24]** I-squared: 47%
van Straten, 2009 Meta-analysis	Moderate	CBT - self-help: stimulus-control; sleep restriction; cognitive therapy; sleep hygiene; relaxation; in-bed exercises: NR<br><br>waiting list: NR	8	sleep diary	Cohen's *d* [95% CI]:<br>-0.01 [-0.14, 0.14] I-squared: 18.8%
Ye, 2016 Meta-analysis	Moderate	CBT - sleep hygiene education; cognitive restructuring; stimulus control; sleep restriction; relaxation therapy; hierarchy development; imagery training; scheduled pseudo desensitization; breathing control: 1006<br><br>wait-list control, treatment as usual; internet + email; internet + telephone; telephone; internet-based control: 1003	15	sleep diary	Mean difference [95% CI]:<br>**22.3 [16.38, 28.23]** I-squared: 12%
Dickerson, 2014 Systematic Review	High	CBT<br><br>usual treatment; waitlist crossover; waitlist control; control; usual treatment<br><br>Total sample: 150	1	actigraphy	Change in outcome:<br>CBT vs placebo: **-0.81 [-1.21, -0.42]**<br>CBT had a **moderate effect** in decreasing insomnia symptoms
		CBT: 12<br><br>No comparator (pre- post-intervention)	1	sleep diary	Change in outcome:<br>Pre-/post scores: 0.47 [-0.27, 1.350]

Author, Year Synthesis type	AMSTAR rating	Intervention and sample size	# of studies	Measurement method	Pooled Estimates (MA) or Narrative Results (from SR with no MA)
Venables, 2014 Systematic Review	Critically Low	group CBT: 660<br><br>No comparator (pre- post-intervention)	8	NR	7 of 8 studies obtained improvements in total sleep time; group delivered CBT sessions may be slightly more effective than individual sessions.
		professionally administered CBT: 615<br><br>No comparator (pre- post-intervention)	8	actigraphy<br>sleep diary	4 of 8 studies reported increased TST in the intervention group, 4 of 8 found no significant increase in TST in the intervention or control groups
Ho, 2015 Meta-analysis	Low	Multi-component CBT - stimulus control, sleep restriction, sleep hygiene, relaxation and/or cognitive therapy: NR<br><br>waiting-list control; routine care or no treatment: NR	8	sleep diary	Hedges *g* [95% CI]:<br>**0.31 [0.0, 0.6]** I-squared: 78%
Okajima, 2011 Meta-analysis	Critically Low	Multi-component CBT - sleep hygiene education; sleep restriction; stimulus control; cognitive therapy; relaxation; paradoxical intention: NR<br><br>wait-list control, placebo; sleep hygiene education; control(unspecified); treatment as usual: NR	7	sleep diary	Cohen's *d* [95% CI]:<br>**0.21 [0.03, 0.39]** I-squared: NR
			2	polysomnography<br>actigraphy	Cohen's *d* [95% CI]:<br>**0.71 [0.21, 1.12]** I-squared: NR
Trauer, 2015 Meta-analysis	Moderate	Multi-component CBT - cognitive therapy, stimulus control, sleep restriction, relaxation, sleep hygiene: NR<br><br>WLC/TAU, Sleep hygiene, sham, placebo: NR	16	sleep diary	Mean difference [95% CI]:<br>7.61 [-0.51, 15.74] I-squared: 3.1%

Author, Year<br>Synthesis type	AMSTAR rating	Intervention and sample size	# of studies	Measurement method	Pooled Estimates (MA) or<br>Narrative Results (from SR with no MA)
Zachariae, 2016<br>Meta-analysis	Moderate	Multi-component CBT - stimulus control, sleep hygiene, cognitive therapy, sleep restriction, relaxation techniques: NR<br><br>wait-list control; treatment as usual: NR	10	NR	Hedges *g* [95% CI]:<br>**0.29 [0.17, 0.42]** I-squared: 5.4%
Howell, 2014<br>Systematic Review	Moderate	Multi-component CBT - sleep hygeine, sleep restriction, stimulus control, cognitive restructuring, and relaxation therapies<br><br>usual care, wait-list control, healthy eating and nutrition, sleep education and hygiene only, no treatment<br><br>Total sample: 369	2	actigraphy	Non-significant change was identified between control and treatment groups for total sleep time
van Straten, 2009<br>Meta-analysis	Moderate	self-help CBT: stimulus-control; sleep restriction; cognitive therapy; sleep hygiene; relaxation; in-bed exercises: NR<br><br>In-person CBT: NR	3	sleep diary	Cohen's *d* [95% CI]:<br>-0.05 [-0.40, 0.31] I-squared: 50.9%
Seyffert, 2016<br>Meta-analysis	Low	Internet-based CBT - sleep education, stimulus control, sleep restriction, relaxation, sleep hygiene, cognitive techniques: NR<br><br>In-person CBT: NR	2	sleep diary	Mean difference [95% CI]:<br>0.73 [-311.8, 313.3] I-squared: 75%
Wang, 2005<br>Systematic Review	Moderate	CBT: stimulus control; sleep restriction; sleep hygiene education<br><br>Relaxation therapy<br><br>Total sample: 46	1	sleep log<br>polysomnography	Change in outcome:<br>CBT: 352.1→372.4 mins;<br>relaxation: 352.1→337.9 mins

Author, Year<br>Synthesis type	AMSTAR rating	Intervention and sample size	# of studies	Measurement method	Pooled Estimates (MA) or<br>Narrative Results (from SR with no MA)
Wake after sleep onset					
Brasure, 2015<br>Meta-analysis	High	CBT-I: 412 [general adult population]<br><br>sham treatment/ placebo, wait-list control, no treatment, or sleep hygiene/sleep education): 420	11	subjective report	Mean difference [95% CI]:<br>**-22.33 [-37.44, -7.21]** I-squared: 89%
		CBT-I: 124 [older adults]<br><br>placebo, wait-list control, no treatment, or sleep hygiene/sleep education: 96	4	subjective report	Mean difference [95% CI]:<br>**-26.96 [-35.73, -18.19]** I-squared: 0%
		CBT-I: 61 [adults with chronic pain]<br><br>passive control (placebo or sham treatment or wait-list): 61	3	subjective report	Mean difference [95% CI]:<br>**-38.18 [-65.57, -10.78]** I-squared: 0.82%
Cheng, 2012<br>Meta-analysis	Low	CBT - sleep hygiene; stimulus control; relaxation training; sleep restriction; cognitive restructuring: NR<br><br>wait list control: NR	4	sleep diary	Standardized mean difference [95% CI]:<br>-0.18 [-0.43, 0.06] I-squared: 55%
Ho, 2016<br>Meta-analysis	Low	CBT - image rehearsal therapy; exposure, re-scripting and relaxation therapy; mind-body bridging; behavioural sleep intervention: NR<br><br>wait list control; sleep hygiene: NR	4	sleep diary	Standardized mean difference [95% CI]:<br>**-1.02 [-1.32, -0.66]** I-squared: 0%
Irwin, 2006<br>Meta-analysis	Critically Low	CBT – relaxation, biofeedback, hypnosis, sleep compression/restriction, paradoxical intention: NR<br><br>control: NR	15	self-report	Cohen's *d* [95% CI]:<br>**-0.64 [-0.82, -0.47]** Q-statistic: 21.65

Author, Year Synthesis type	AMSTAR rating	Intervention and sample size	# of studies	Measurement method	Pooled Estimates (MA) or Narrative Results (from SR with no MA)
Johnson, 2016 Meta-analysis	Moderate	CBT-I - with both cognitive and behavioral components: 423<br><br>waitlist control; treatment-as-usual; sleep education; behavioral placebo; mindfulness-based stress reduction: 297	8	sleep diary	Cohen's *d* [95% CI]: **0.29 [0.10 to 0.48]** I-squared: 30.1%
Koffel, 2015 Meta-analysis	Critically Low	group CBT-I - stimulus control, sleep restriction, and addressing dysfunctional beliefs about sleep: NR<br><br>wait list, treatment as usual, placebo: NR	6	sleep diary	Mean effect size [95% CI]: **0.65 [0.26, 1.04]** I-squared: NR
Montgomery, 2003 Meta-analysis	High	CBT - sleep hygiene; stimulus control; muscle relaxation; sleep restriction; cognitive therapy; education; imagery training: 95<br><br>wait-list control, placebo: 64	4	Sleep Diary	Mean difference [95% CI]: **-21.84 [-37.30, -6.38]** I-squared: 55%
		CBT -sleep hygiene; stimulus control; muscle relaxation; sleep restriction; cognitive therapy; education; imagery training: 30<br><br>wait-list control, placebo: 29	2	polysomnography	Mean difference [95% CI]: **-24.36 [-41.14, -7.57]** I-squared: 0%
Navarro-Bravo, 2015 Meta-analysis	Moderate	CBT - sleep restriction, stimulus control, sleep education/hygiene: NR<br><br>placebo; wait list control; stress management and wellness training; treatment as usual; sleep hygiene/education: NR	7	sleep diary<br>polysomnography<br>actigraphy<br>sleep evaluation (4-item questionnaire)	Cohen's *d* [95% CI]: **-0.68 [-1.11, -0.26]** Chi square: 34.43; 0.000

Author, Year<br>Synthesis type	AMSTAR rating	Intervention and sample size	# of studies	Measurement method	Pooled Estimates (MA) or<br>Narrative Results (from SR with no MA)
Seyffert, 2016<br>Meta-analysis	Low	Internet-based CBT -sleep education, stimulus control, sleep restriction, relaxation, sleep hygiene, cognitive techniques: NR<br><br>wait-list control, Internet control, TAU: NR	6	Sleep diary	Mean difference [95% CI]:<br>**-20.44 [-34.87, -6.01]** I-squared: 69.3%
van Straten, 2007<br>Meta-analysis	Moderate	CBT - relaxation; sleep restriction; stimulus control; paradoxical intention; identifying and challenging dysfunctional thoughts: NR<br><br>waitlist control; no treatment; placebo; psycho-education: NR	71	sleep diary	Hedges *g* [95% CI]:<br>**0.63 [0.53, 0.73]** I-squared: 60%
van Straten, 2009<br>Meta-analysis	Moderate	CBT -self-help: stimulus-control; sleep restriction; cognitive therapy; sleep hygiene; relaxation; in-bed exercises: NR<br><br>waiting list: NR	6	sleep diary	Cohen's *d* [95% CI]:<br>**0.3 [0.13, 0.48]** I-squared: 63.9%
Ye, 2016<br>Meta-analysis	Moderate	CBT - sleep hygiene education; cognitive restructuring; stimulus control; sleep restriction; relaxation therapy; hierarchy development; imagery training; scheduled pseudo desensitization; breathing control: 828<br><br>wait-list control, treatment as usual; internet + email; internet + telephone; telephone; internet-based control: 827	11	sleep diary	Mean difference [95% CI]:<br>**-22.31 [-31.11, -13.50]** I-squared: 76%
Brooks, 2014<br>Systematic Review	Low	CBT-I: NR<br><br>Control NR: NR	1	daily sleep diary; Pittsburgh Sleep Quality Index; actigraphy	WASO improved in treatment group based on self-reported data and effect remained for 6 months post-treatment; not corroborated by actigraphy

Author, Year<br>Synthesis type	AMSTAR rating	Intervention and sample size	# of studies	Measurement method	Pooled Estimates (MA) or<br>Narrative Results (from SR with no MA)
Dickerson, 2014<br>Systematic Review	High	CBT(unspecified)<br><br>usual treatment; waitlist crossover; waitlist control; control; usual treatment<br><br>Total sample: 150	1	Actigraphy	Change in outcome:<br>CBT vs placebo: **-0.50 [-0.89 to -0.1]**<br>CBT showed a moderate effect in decreasing insomnia symptoms compared to control
		CBT (unspecified): 12<br><br>No comparator (pre- post-intervention)	1	sleep diary	Change in outcome:<br>Pre/post scores<br>-1.18 [-2.45 to 0.62]
Venables, 2014<br>Systematic Review	Critically Low	group CBT: 660<br><br>No comparison	8	NR	8 of 8 studies demonstrated improvement in WASO (p-values not reported); results demonstrate that group delivered CBT sessions may be slightly more effective than individual sessions
		professionally administered CBT: 615<br><br>No comparison	8	actigraphy<br>sleep diary	8 of 8 studies in professionally administered CBT reported an increase in WASO (p-values not reported)
Wang, 2005<br>Systematic Review	Moderate	CBT - stimulus control; sleep restriction; cognitive therapy; sleep hygiene education; sleep scheduling<br><br>control; Quasi desensitization; self-monitoring control; sleep hygiene recommendations; waiting-list control<br><br>Total sample: 162	3	polysomnography<br>sleep log	**significant improvement** in WASO for CBT compared to placebo **(p<0.05)**;<br>CBT group averaged a 52% reduction in WASO from study entry to 3-month follow-up time point; 60% of CBT group and none of control group achieved the criterion for clinically significant WASO improvement
Ho, 2015<br>Meta-analysis	Low	Multi-component CBT - stimulus control, sleep restriction, sleep hygiene, relaxation and/or cognitive therapy: NR<br><br>waiting-list control; routine care or no treatment: NR	6	sleep diary	Hedges *g* [95% CI]:<br>**-0.74 [-1.3, -0.2]** I-squared: 93%

Author, Year Synthesis type	AMSTAR rating	Intervention and sample size	# of studies	Measurement method	Pooled Estimates (MA) or Narrative Results (from SR with no MA)
Okajima, 2011 Meta-analysis	Critically Low	Multi-component CBT - sleep hygiene education; sleep restriction; stimulus control; cognitive therapy; relaxation; paradoxical intention: NR<br><br>wait-list control, placebo; sleep hygiene education; control(unspecified); treatment as usual: NR	6	sleep diary	Cohen's *d* [95% CI]:<br>**0.34 [0.15, 0.52]** I-squared: NR
Trauer, 2015 Meta-analysis	Moderate	Multi-component CBT - cognitive therapy, stimulus control, sleep restriction, relaxation, sleep hygiene: NR<br><br>Wait-list control, treatment as usual, Sleep hygiene, sham, placebo: NR	14	Sleep diary	Mean difference [95% CI]:<br>-26 [-36.52, 15.48] I-squared: 47.2%
Zachariae, 2016 Meta-analysis	Moderate	Multi-component CBT -stimulus control, sleep hygiene, cognitive therapy, sleep restriction, relaxation techniques: NR<br><br>Wait-list control, treatment as usual: NR	7	NR	Hedges *g* [95% CI]:<br>**0.45 [0.25, 0.66]** I-squared: 48.5%
Howell, 2014 Systematic Review	Moderate	Multi-component CBT -sleep hygiene, sleep restriction, stimulus control, cognitive restructuring, and relaxation therapies<br><br>usual care, wait-list control, healthy eating and nutrition, sleep education and hygiene only, no treatment<br><br>Total sample: 207	2	actigraphy<br>sleep diary	2 of 2 studies found **significant improvement** in WASO for undergoing CBT

Author, Year<br>Synthesis type	AMSTAR rating	Intervention and sample size	# of studies	Measurement method	Pooled Estimates (MA) or<br>Narrative Results (from SR with no MA)
McCurry, 2007<br>Systematic Review	Critically Low	Multi-component CBT -sleep hygiene, relaxation, sleep compression, cognitive behavioural therapy, stimulus control<br><br>delayed treatment, wait-list control, placebo; stress management:<br><br>Total sample: 154	3	sleep logs	CBT **significantly improved** WASO compared to stress management and wait-list control
		Multi-component CBT -sleep restriction, education, stimulus control: 4<br><br>No comparator (pre- post-intervention)	1	Sleep logs<br>sleep assessment device	Effect size (p-value): 1.12 (NR)<br>Large effect size supports the effectiveness of CBT to improve WASO (p-values not reported)
van Straten, 2009<br>Meta-analysis	Moderate	self-help CBT: stimulus-control; sleep restriction; cognitive therapy; sleep hygiene; relaxation; in-bed exercises: NR<br><br>In-person CBT: NR	3	sleep diary	Cohen's *d* [95% CI]:<br>-0.03 [-0.32, 0.38] I-squared: 44.5%
Wang, 2005<br>Systematic Review	Moderate	CBT - stimulus control; sleep restriction; sleep hygiene education<br><br>Relaxation therapy<br><br>Total sample: 46	1	polysomnography<br>sleep log	Change in outcome:<br>CBT: 50.8→30.1minutes<br>relaxation: 50.8→50.6minutes<br>**Significant improvement** in WASO for CBT compared to relaxation therapy; CBT recipients reported a 54% reduction whereas relaxation group reported 16% **($p<0.01$)**
McCurry, 2007<br>Systematic Review	Critically Low	CBT (unspecified)<br><br>CBT (unspecified) + Temazepam<br><br>Total sample: 78	1	sleep logs	CBT and CBT + temazepam groups both showed **significant improvement** compared to placebo

Author, Year<br>Synthesis type	AMSTAR rating	Intervention and sample size	# of studies	Measurement method	Pooled Estimates (MA) or<br>Narrative Results (from SR with no MA)
Buscemi, 2005<br>Meta-analysis	High	CBT + relaxation technique - relaxation training; cognitive control; stimulus control; group relaxation; aggressive muscle relaxation; cognitive distraction: 23<br><br>placebo: 26	2	NR	Mean difference [95% CI]:<br>-7.6 [-26.3, 11.1] I-squared: 0%
		Multi-component CBT; Paradoxical Intention; Sleep compression; Stimulus control:128<br><br>Placebo: 120	8	sleep diary	Mean difference [95% CI]:<br>**-18.17 [-30.37, -5.98]** I-squared: 52.9%
		CBT + relaxation - relaxation training; stimulus control; aggressive muscle relaxation; cognitive distraction: 23<br><br>CBT - cognitive therapy; sleep restriction; stimulus control; sleep hygiene: 24	2	NR	Mean difference [95% CI]:<br>5.1 [-12.0, 22.2] I-squared: 0%
Sleep Quality					
Brasure, 2015<br>Meta-analysis	High	CBT-I: 296<br><br>sham treatment/ placebo, wait-list control, no treatment, or sleep hygiene/sleep education): 284	6	Pittsburgh Sleep Quality Index	Mean difference [95% CI]:<br>**-2.1 [-2.87, -1.34]** I-squared: 37%
Ho, 2016<br>Meta-analysis	Low	CBT - image rehearsal therapy; exposure, re-scripting and relaxation therapy; mind-body bridging; behavioural sleep intervention: NR<br><br>wait list control; sleep hygiene: NR	6	Pittsburgh sleep quality index	Standardized mean difference [95% CI]:<br>**-0.87 [-1.18, -0.56]** I-squared: 33%
Irwin, 2006<br>Meta-analysis	Critically Low	CBT – relaxation, biofeedback, hypnosis, sleep compression/restriction, paradoxical intention: NR<br><br>control: NR	7	self-report	Cohen's *d* [95% CI]:<br>**0.76 [0.48 to 1.03]** Q-statistic: 7.92

Author, Year Synthesis type	AMSTAR rating	Intervention and sample size	# of studies	Measurement method	Pooled Estimates (MA) or Narrative Results (from SR with no MA)
Koffel, 2015 Meta-analysis	Critically Low	group CBT-I - stimulus control, sleep restriction, and addressing dysfunctional beliefs about sleep: NR<br><br>wait list, treatment as usual, placebo: NR	5	Sleep Diary, Sleep Quality Measures	Mean effect size [95% CI]:<br>0.4 [-0.14, 0.93] I-squared: NR
Navarro-Bravo, 2015 Meta-analysis	Moderate	CBT - sleep restriction, stimulus control, sleep education/hygiene: NR<br><br>placebo; wait list control; stress management and wellness training; treatment as usual; sleep hygiene/education: NR	5	Pittsburgh Sleep Quality Index	Cohen's *d* [95% CI]:<br>**-0.59 [-0.59, -0.85]** Chi square: 6.85; 0.144
Tang, 2015 Meta-analysis	Moderate	CBT-I: psychoeducation, sleep hygiene, stimulus control, sleep restriction, cognitive therapy, and relaxation: 510<br><br>waitlist, treatment as usual: 455	11	Pittsburgh Sleep Quality Index (PSQI); Insomnia Severity Index (ISI)	Standardized mean difference [95% CI]:<br>**0.78 [0.42, 1.13]**<br>I-squared: 84%
van Straten, 2007 Meta-analysis	Low	CBT - relaxation; sleep restriction; stimulus control; paradoxical intention; identifying and challenging dysfunctional thoughts: NR<br><br>waitlist control; no treatment; placebo; psycho-education: NR	19	Pittsburgh sleep quality index	Hedges *g* [95% CI]:<br>**0.65 [0.51, 0.79]** I-squared: 39%
			40	sleep diary	Hedges *g* [95% CI]:<br>**0.4 [0.24, 0.56]** I-squared: 74%
van Straten, 2009 Meta-analysis	Moderate	CBT –self-help: stimulus-control; sleep restriction; cognitive therapy; sleep hygiene; relaxation; in-bed exercises: NR<br><br>waiting list: NR	7	sleep diary	Cohen's *d* [95% CI]:<br>**0.21 [0.06, 0.35]** I-squared: 52.3%
Cheng, 2012 Meta-analysis	Low	sleep hygiene; stimulus control; relaxation training; sleep restriction; cognitive restructuring: NR<br><br>wait list control: NR	4	Pittsburgh sleep quality index	Standardized mean difference [95% CI]:<br>**0.41 [0.16, 0.65]** I-squared: 45%

Author, Year<br>Synthesis type	AMSTAR rating	Intervention and sample size	# of studies	Measurement method	Pooled Estimates (MA) or<br>Narrative Results (from SR with no MA)
Bogdanov, 2017<br>Systematic Review	Low	CBT (unspecified)<br>Control (unspecified)<br>Total sample: 3	1	Pittsburgh sleep quality index	No clinically meaningful improvement in either group
Venables, 2014<br>Systematic Review	Critically Low	professionally administered CBT: 215<br><br>No comparator (pre- post-intervention)	2	Pittsburgh sleep quality index	Change in outcome [mean change (%)]:<br>7.3 (37.6)<br>2 of 2 studies found a reduction in PSQI scores (p-values not reported); neither of the scores decreased to below 5.0, above which is a diagnostic score for insomnia
Ho, 2015<br>Meta-analysis	Low	Multi-component CBT - stimulus control, sleep restriction, sleep hygiene, relaxation and/or cognitive therapy: NR<br><br>waiting-list control; routine care or no treatment: NR	4	sleep diary	Hedges *g* [95% CI]:<br>**0.43 [0.2, 0.6]** I-squared: 0%
Okajima, 2011<br>Meta-analysis	Critically Low	Multi-component CBT –sleep hygiene education; sleep restriction; stimulus control; cognitive therapy; relaxation; paradoxical intention: NR<br><br>wait-list control, placebo; sleep hygiene education; control(unspecified); treatment as usual: NR	2	Pittsburgh Sleep Quality Index	Cohen's *d* [95% CI]:<br>**0.77 [0.48, 0.97]** I-squared: NR
Zachariae, 2016<br>Meta-analysis	Moderate	Multi-component CBT –stimulus control, sleep hygiene, cognitive therapy, sleep restriction, relaxation techniques: NR<br><br>Wait-list control, treatment as usual: NR	8	NR	Hedges g [95% CI]:<br>**0.49 [0.30, 0.68]** I-squared: 34.5%

Author, Year<br>Synthesis type	AMSTAR rating	Intervention and sample size	# of studies	Measurement method	Pooled Estimates (MA) or<br>Narrative Results (from SR with no MA)
Howell, 2014<br>Systematic Review	Moderate	Multi-component CBT –sleep hygiene, sleep restriction, stimulus control, cognitive restructuring, and relaxation therapies<br><br>usual care, wait-list control, healthy eating and nutrition, sleep education and hygiene only, no treatment<br><br>Total sample: 233	2	actigraphy<br>sleep diary	**Significant improvement** in all studies post CBT intervention identified for sleep quality (p-values not reported)
McCurry, 2007<br>Systematic Review	Critically Low	Multi-component CBT –sleep hygiene, relaxation, sleep compression, cognitive behavioural therapy, stimulus control<br><br>delayed treatment, wait-list control, placebo; stress management:<br><br>Total sample: 210	4	sleep logs<br>Pittsburgh sleep quality index	**Significant improvement** in sleep quality ratings compared to control
Buscemi, 2005<br>Meta-analysis	High	CBT + relaxation - relaxation training; cognitive control; stimulus control; group relaxation; aggressive muscle relaxation; cognitive distraction: 23<br><br>placebo: 26	2	NR	Mean difference [95% CI]:<br>0.69 [-0.34, 1.73] I-squared: 65.4%
Yang, 2014<br>Meta-analysis	Moderate	CBT + relaxation - CBT; sleep hygiene; relaxation CD: 93<br><br>Sleep hygiene education; treatment as usual: 91	3	global Pittsburgh sleep quality index	Standardized mean difference [95% CI]:<br>**0.85 [0.37, 1.34]** I-squared: 56%
		CBT + relaxation - CBT; sleep hygiene; relaxation CD: 56<br><br>Sleep hygiene education; treatment as usual: 56	2	subjective sleep quality in Pittsburgh Sleep Quality Index	Standardized mean difference [95% CI]:<br>0.44 [-0.28, 1.17] I-squared: 64%

Author, Year<br>Synthesis type	AMSTAR rating	Intervention and sample size	# of studies	Measurement method	Pooled Estimates (MA) or<br>Narrative Results (from SR with no MA)
van Straten, 2009<br>Meta-analysis	Moderate	self-help CBT: stimulus-control; sleep restriction; cognitive therapy; sleep hygiene; relaxation; in-bed exercises: NR<br><br>In-person CBT: NR	2	sleep diary	Cohen's *d* [95% CI]:<br>-0.5 [-0.90, 0.02] I-squared: 0%
Buscemi, 2005<br>Meta-analysis	High	CBT + relaxation - relaxation training; stimulus control; aggressive muscle relaxation; cognitive distraction: 23<br><br>CBT - cognitive therapy; sleep restriction; stimulus control; sleep hygiene: 24	2	NR	Mean difference [95% CI]:<br>0.2 [-0.38, 0.77] I-squared: 0%
Sleep Satisfaction					
Wang, 2005<br>Systematic Review	Moderate	CBT - stimulus control; sleep restriction; cognitive therapy; sleep hygiene education; sleep scheduling<br><br>control; Quasi desensitization; self-monitoring control; sleep hygiene recommendations; waiting-list control<br><br>Total sample: 81	2	Dysfunctional attitudes and Beliefs about Sleep (DBAS) evaluation; The Beliefs and Attitudes about Sleep Scale	CBT provided greater improvements in DBAS scores than did placebo, CBT endorsed less dysfunctional beliefs and attitudes about sleep than the placebo group; **significantly greater improvement** in BAS scores
Sleep efficiency					
Cheng, 2012<br>Meta-analysis	Low	CBT - sleep hygiene; stimulus control; relaxation training; sleep restriction; cognitive restructuring: NR<br><br>wait list control: NR	4	sleep diary	Standardized mean difference [95% CI]:<br>**0.4 [0.15, 0.64]** I-squared: 63%
Ho, 2016<br>Meta-analysis	Low	CBT - image rehearsal therapy; exposure, re-scripting and relaxation therapy; mind-body bridging; behavioural sleep intervention: NR<br><br>wait list control; sleep hygiene: NR	5	sleep diary	Standardized mean difference [95% CI]:<br>**1.15 [0.75, 1.56]** I-squared: 37%

Author, Year<br>Synthesis type	AMSTAR rating	Intervention and sample size	# of studies	Measurement method	Pooled Estimates (MA) or<br>Narrative Results (from SR with no MA)
Irwin, 2006<br>Meta-analysis	Critically Low	CBT – relaxation, biofeedback, hypnosis, sleep compression/restriction, paradoxical intention: NR<br><br>control: NR	8	self-report	Cohen's *d* [95% CI]:<br>**0.52 [0.28 to 0.75]** Q-statistic: 47.85
Johnson, 2016<br>Meta-analysis	Moderate	CBT-I with both cognitive and behavioral components: 423<br><br>waitlist control; treatment-as-usual; sleep education; behavioral placebo; mindfulness-based stress reduction: 297	8	sleep diary	Cohen's *d* [95% CI]:<br>**0.33 [0.11 to 0.54]** I-squared: 41.1%
Koffel, 2015<br>Meta-analysis	Critically Low	group CBT-I - stimulus control, sleep restriction, and addressing dysfunctional beliefs about sleep: NR<br><br>wait list, treatment as usual, placebo: NR	6	sleep diary	Mean effect size [95% CI]:<br>**0.84 [0.38, 1.31]** I-squared: NR
Montgomery, 2003<br>Meta-analysis	High	CBT - cognitive-behavioural therapy (unspecified); sleep hygiene; stimulus control; muscle relaxation; sleep restriction; cognitive therapy; education; imagery training: 86<br><br>wait-list control, placebo: 57	3	sleep diary	Mean difference [95% CI]:<br>-7.49 [-15.45, 0.47] I-squared: 77%
		CBT - cognitive-behavioural therapy (unspecified); sleep hygiene; stimulus control; muscle relaxation; sleep restriction; cognitive therapy; education; imagery training: 30<br><br>wait-list control, placebo: 29	2	polysomnography	Mean difference [95% CI]:<br>**-6.25 [-10.18, -2.31]** I-squared: 0%

Author, Year<br>Synthesis type	AMSTAR rating	Intervention and sample size	# of studies	Measurement method	Pooled Estimates (MA) or<br>Narrative Results (from SR with no MA)
Navarro-Bravo, 2015<br>Meta-analysis	Moderate	CBT - sleep restriction, stimulus control, sleep education/hygiene: NR<br><br>placebo; wait list control; stress management and wellness training; treatment as usual; sleep hygiene/education: NR	8	sleep diary<br>polysomnography<br>actigraphy<br>sleep evaluation (4-item questionnaire)	Cohen's *d* [95% CI]:<br>**0.78 [0.34, 1.21]** Chi square: 47.56; 0.000
Seyffert, 2016<br>Meta-analysis	Low	Internet-based CBT - sleep education, stimulus control, sleep restriction, relaxation, sleep hygiene, cognitive techniques: NR<br><br>Wait-list control, Internet control, treatment as usual: NR	9	Sleep diary	Mean difference [95% CI]:<br>**7.22 [5.13, 9.32]** I-squared: 39.5%
van Straten, 2007<br>Meta-analysis	Low	CBT - relaxation; sleep restriction; stimulus control; paradoxical intention; identifying and challenging dysfunctional thoughts: NR<br><br>waitlist control; no treatment; placebo; psycho-education: NR	79	sleep diary	Hedges *g* [95% CI]:<br>**0.71 [0.61, 0.82]** I-squared: 70%
van Straten, 2009<br>Meta-analysis	Moderate	CBT - self-help: stimulus-control; sleep restriction; cognitive therapy; sleep hygiene; relaxation; in-bed exercises: NR<br><br>waiting list: NR	7	sleep diary	Cohen's *d* [95% CI]:<br>**0.26 [0.11, 0.40]** I-squared: 65.5%

**Author, Year Synthesis type**	**AMSTAR rating**	**Intervention and sample size**	**# of studies**	**Measurement method**	**Pooled Estimates (MA) or Narrative Results (from SR with no MA)**
Ye, 2016 Meta-analysis	Moderate	CBT - sleep hygiene education; cognitive restructuring; stimulus control; sleep restriction; relaxation therapy; hierarchy development; imagery training; scheduled pseudo desensitization; breathing control: 1006<br><br>wait-list control, treatment as usual; internet + email; internet + telephone; telephone; internet-based control: 1003	15	sleep diary	Mean difference [95% CI]: **9.58 [7.30, 11.85]** I-squared: 76%
Bogdanov, 2017 Systematic Review	Low	CBT (unspecified)<br>Control (unspecified)<br>Total sample: 11	1	sleep diary	**Significant improvement** in sleep efficiency compared to control **(p=0.01)**
Brooks, 2014 Systematic Review	Low	CBT-I: 7<br>No comparator (pre- post-intervention)	1	daily sleep diary	Improvement in sleep efficiency (p-values not reported)
		CBT-I (unspecified): NR<br>Control (unspecified): NR	2	daily sleep diary<br>Pittsburgh Sleep Quality Index<br>actigraphy	Self-reported sleep efficiency improved in treatment group; maintained for 6 months post-treatment; not corroborated by actigraphy
Dickerson, 2014 Systematic Review	High	CBT (unspecified): 12<br>No comparator (pre- post-intervention)	1	sleep diary	Change in outcome:<br>Pre-/post scores **1.49 [0.88 to 2.79]**<br>Sleep efficiency improved over baseline at weeks 4 and 8
Ishak, 2012 Systematic Review	Low	CBT (unspecified)<br>placebo, no treatment, usual care<br>Total sample: 209	1	SF-36; CIS-20; GHQ; PANAS; FACT-G	**Significant improvements** in sleep efficiency at both 3 and 6-month follow-up **(p<0.01)**

Author, Year<br>Synthesis type	AMSTAR rating	Intervention and sample size	# of studies	Measurement method	Pooled Estimates (MA) or<br>Narrative Results (from SR with no MA)
Wang, 2005<br>Systematic Review	Moderate	CBT - stimulus control; sleep restriction; cognitive therapy; sleep hygiene education; sleep scheduling<br><br>control; Quasi desensitization; self-monitoring control; sleep hygiene recommendations; waiting-list control<br><br>Total sample: 128	4	sleep diary/log<br>polysomnography<br>structured interview	4 of 4 studies found improvement in sleep efficiency compared to control; only 2 of 4 were **statistically significant**
Taylor, 2014<br>Systematic Review	Critically Low	CBT - stimulus control, sleep restriction, relaxation therapy, cognitive therapy; image rehearsal therapy; medication withdrawal: NR<br><br>wait list, usual care, sleep hygiene, placebo control: NR	13	sleep diaries	Mean effect size [95% CI; p-value]:<br>**0.758 [0.557, 0.958; p<0.01]**<br>CBT-I results in **significant improvement** in sleep efficiency in patients with co-morbid psychiatric disorders, medium to large effects were homogeneous across studies
Venables, 2014<br>Systematic Review	Critically Low	group CBT: 660<br>No comparator (pre- post-intervention)	8	NR	8 of 8 studies obtained improvements in sleep quality, results demonstrate that group delivered CBT sessions may be slightly more effective than individual sessions
		professionally administered CBT: 615<br>No comparator (pre- post-intervention)	8	actigraphy<br>sleep diary	8 of 8 studies found a **significant improvement** in sleep efficiency (p-values not reported)
Ho, 2015<br>Meta-analysis	Low	Multi-component CBT - stimulus control, sleep restriction, sleep hygiene, relaxation and/or cognitive therapy: NR<br><br>waiting-list control; routine care or no treatment: NR	7	sleep diary	Hedges *g* [95% CI]:<br>**0.79 [0.2, 1.4]** I-squared: 92%
Okajima, 2011<br>Meta-analysis	Critically Low	Multi-component CBT - sleep hygiene education; sleep restriction;	8	sleep diary	Cohen's *d* [95% CI]:<br>**0.43 [0.25, 0.59]** I-squared: NR

Author, Year Synthesis type	AMSTAR rating	Intervention and sample size	# of studies	Measurement method	Pooled Estimates (MA) or Narrative Results (from SR with no MA)
		stimulus control; cognitive therapy; relaxation; paradoxical intention: NR<br><br>wait-list control, placebo; sleep hygiene education; control(unspecified); treatment as usual: NR	2	polysomnography actigraphy	Cohen's *d* [95% CI]: **0.78 [0.27, 1.17]** I-squared: NR
Trauer, 2015 Meta-analysis	Moderate	Multi-component CBT - cognitive therapy, stimulus control, sleep restriction, relaxation, sleep hygiene: NR<br><br>Wait-list control, treatment as usual, Sleep hygiene, sham, placebo: NR	17	Sleep diary	Mean difference [95% CI]: **9.91 [8.09, 11.73]** I-squared: 47.1%
Zachariae, 2016 Meta-analysis	Moderate	Multi-component CBT - stimulus control, sleep hygiene, cognitive therapy, sleep restriction, relaxation techniques: NR<br><br>Wait-list control, treatment as usual: NR	10	NR	Hedges *g* [95% CI]: **0.58 [0.36, 0.81]** I-squared: 68.4%
Howell, 2014 Systematic Review	Moderate	Multi-component CBT - sleep hygiene, sleep restriction, stimulus control, cognitive restructuring, and relaxation therapies<br><br>usual care, wait-list control, healthy eating and nutrition, sleep education and hygiene only, no treatment<br><br>Total sample: 209	1	SF-36	Sleep efficiency **increased** in all studies post CBT intervention **(p<0.01)**

Author, Year<br>Synthesis type	AMSTAR rating	Intervention and sample size	# of studies	Measurement method	Pooled Estimates (MA) or<br>Narrative Results (from SR with no MA)
McCurry, 2007<br>**Systematic Review**	Critically Low	Multi-component CBT - sleep hygiene, relaxation, sleep compression, cognitive behavioural therapy, stimulus control<br><br>delayed treatment, wait-list control, placebo; stress management:<br><br>Total sample: 154	3	sleep logs	CBT group had **significant improvement** in sleep efficiency compared to stress management
van Straten, 2009<br>Meta-analysis	Moderate	self-help CBT: stimulus-control; sleep restriction; cognitive therapy; sleep hygiene; relaxation; in-bed exercises: NR<br><br>In-person CBT: NR	3	sleep diary	Cohen's *d* [95% CI]:<br>-0.29 [-0.65, 0.06] I-squared: 22.4%
Seyffert, 2016<br>Meta-analysis	Low	Internet-based CBT - sleep education, stimulus control, sleep restriction, relaxation, sleep hygiene, cognitive techniques: NR<br><br>In-person CBT: NR	2	Sleep diary	Mean difference [95% CI]:<br>-1.21 [-49.0, 46.6] I-squared: 59.7%
Wang, 2005<br>Systematic Review	Moderate	CBT - stimulus control; sleep restriction; sleep hygiene education<br><br>CBT + relaxation therapy<br><br>Total sample: 46	1	polysomnography<br>sleep log	Change in outcome:<br>CBT: 77.8%→85.5%;<br>relaxation: 77.8%→78.1%
McCurry, 2007<br>Systematic Review	Critically Low	CBT + Temazepam<br>CBT<br><br>Total sample: 78	1	polysomnography<br>sleep logs	The combination of CBT + temazepam was **significantly more effective** than placebo
Insomnia Severity Index					
Brasure, 2015<br>Meta-analysis	High	CBT-I: 172<br><br>sham treatment/ placebo, wait-list control, no treatment, or sleep hygiene/sleep education): 173	5	ISI	Mean difference [95% CI]:<br>**-5.15 [-7.13, -3.16]** I-squared: 67%

Author, Year Synthesis type	AMSTAR rating	Intervention and sample size	# of studies	Measurement method	Pooled Estimates (MA) or Narrative Results (from SR with no MA)
		CBT-I: 68<br><br>passive control (placebo or sham treatment or wait-list): 63	4	ISI	Mean difference [95% CI]:<br>**-7.1 [-12.87, -1.32]** I-squared: 89%
Cheng, 2012 Meta-analysis	Low	CBT - sleep hygiene; stimulus control; relaxation training; sleep restriction; cognitive restructuring: NR<br><br>wait list control: NR	2	ISI	Standardized mean difference [95% CI]:<br>**-0.86 [-1.18, -0.53]** I-squared: 0%
Ho, 2016 Meta-analysis	Low	CBT - image rehearsal therapy; exposure, re-scripting and relaxation therapy; mind-body bridging; behavioural sleep intervention: NR<br><br>wait list control; sleep hygiene: NR	5	ISI	Standardized mean difference [95% CI]:<br>**-1.15 [-1.81, -0.49]** I-squared: 77%
Johnson, 2016 Meta-analysis	Moderate	CBT-I with both cognitive and behavioral components: NR<br><br>waitlist control; treatment-as-usual; sleep education; behavioral placebo; mindfulness-based stress reduction: NR	4	ISI	Cohen's *d* [95% CI]:<br>**0.547 [0.37 to 0.73]** I-squared: 0%
Navarro-Bravo, 2015 Meta-analysis	Moderate	CBT - sleep restriction, stimulus control, sleep education/hygiene: NR<br><br>placebo; wait list control; stress management and wellness training; treatment as usual; sleep hygiene/education: NR	4	ISI	Cohen's *d* [95% CI]:<br>**-0.7 [-1.1, -0.22]** Chi square: 11.54; 0.009
Seyffert, 2016 Meta-analysis	Low	CBT - sleep education, stimulus control, sleep restriction, relaxation, sleep hygiene, cognitive techniques: NR<br><br>wait-list control, Internet control, treatment as usual: NR	4	Sleep diary	Mean difference [95% CI]:<br>**-3.74 [-7.10, -0.39]** I-squared: 90%

Author, Year Synthesis type	AMSTAR rating	Intervention and sample size	# of studies	Measurement method	Pooled Estimates (MA) or Narrative Results (from SR with no MA)
van Straten, 2007 Meta-analysis	Low	CBT - relaxation; sleep restriction; stimulus control; paradoxical intention; identifying and challenging dysfunctional thoughts: NR<br><br>waitlist control; no treatment; placebo; psycho-education: NR	38	ISI	Hedges *g* [95% CI]: **0.98 [0.82, 1.15]** I-squared: 74%
Ye, 2016 Meta-analysis	Moderate	CBT - sleep hygiene education; cognitive restructuring; stimulus control; sleep restriction; relaxation therapy; hierarchy development; imagery training; scheduled pseudo desensitization; breathing control: 828<br><br>wait-list control, treatment as usual; internet + email; internet + telephone; internet-based control: 827	11	sleep diary (ISI)	Mean difference [95% CI]: **-5.88 [-7.46, -4.29]** I-squared: NR
Bogdanov, 2017 Systematic Review	Low	CBT (unspecified)<br><br>Control (unspecified): NR	2	ISI	**Significant improvement** compared to control **(p<0.01)**; no clinically meaningful improvement
Dickerson, 2014 Systematic Review	High	CBT (unspecified)<br><br>usual treatment; waitlist crossover; waitlist control; control; usual treatment<br><br>Total sample: 72	1	NR	Change in outcome: CBT vs placebo -0.37 [0.10 to 0.84]
		CBT: 10<br>Pre/post intervention; no comparator	1	ISI	Change in outcome: Pre-/post scores 2.67 [1.37-3.73] Treatment **significantly improved** ISI scores
Ishak, 2012 Systematic Review	Low	CBT<br><br>placebo, no treatment, unspecified, usual care<br><br>Total sample: 209	1	ISI	intervention group had reduced insomnia scores at 12-month follow-up when compared with placebo **(p<0.01)**

Author, Year Synthesis type	AMSTAR rating	Intervention and sample size	# of studies	Measurement method	Pooled Estimates (MA) or Narrative Results (from SR with no MA)
Venables, 2014 Systematic Review	Critically Low	group CBT: 660<br><br>No comparison	8	ISI	Change in outcome (average decrease [%]):<br>**53.1; 39.9; 63.9 (p<0.05)**<br>3 of 8 studies had a **significant decrease** in ISI scores
		professionally administered CBT: 132<br><br>No comparison	5	ISI	Change in outcome (average decrease [%]):<br>58.2; 53.1; 63.9; 27.4; 39.9 (p=NR)<br>5 of 5 studies found a reduction in ISI scores
		self-help CBT: 328<br><br>No comparison	4	ISI	Change in outcome (average decrease [%]):<br>45.2; 44.5; 52; 56.2 (p=NR)<br>4 of 4 studies found a non-significant decrease in IS Scores
Zachariae, 2016 Meta-analysis	Moderate	Multi-component CBT - stimulus control, sleep hygiene, cognitive therapy, sleep restriction, relaxation techniques: NR<br><br>Wait-list control; treatment as usual: NR	8	ISI	Hedges *g* [95% CI]:<br>**1.09 [0.74, 1.45]** I-squared: 82.8%
Howell, 2014 Systematic Review	Moderate	Multi-component CBT -sleep hygiene, sleep restriction, stimulus control, cognitive restructuring, and relaxation therapies<br><br>usual care, wait-list control, healthy eating and nutrition, sleep education and hygiene only, no treatment:<br><br>Total sample: 180	4	ISI	Overall **significant improvement** in ISI after CBT intervention
Yang, 2014 Meta-analysis	Moderate	CBT + relaxation - CBT; sleep hygiene; relaxation CD: 56<br><br>Sleep hygiene education; treatment as usual: 56	2	habitual sleep efficiency in Pittsburgh sleep quality index	Standardized mean difference [95% CI]:<br>-0.43 [-1.68, 0.83] I-squared: 86%

Author, Year<br>Synthesis type	AMSTAR rating	Intervention and sample size	# of studies	Measurement method	Pooled Estimates (MA) or<br>Narrative Results (from SR with no MA)
Seyffert, 2016<br>Meta-analysis	Low	Self-help CBT - sleep education, stimulus control, sleep restriction, relaxation, sleep hygiene, cognitive techniques: NR<br><br>In-person CBT: NR	2	Sleep diary	Mean difference [95% CI]:<br>1.07 [-6.23, 8.38] I-squared: 0%
Fatigue Severity					
Ballesio, 2017<br>Meta-analysis	High	CBT-I Group: 302<br><br>sleep hygiene, wait-list control, pharmacological, placebo, psychological: 226	6	fatigue symptom inventory, multidimensional fatigue symptom inventory, Flinder fatigue scale, multidimensional fatigue inventory, Krupp fatigue scale	Cohen's *d* [95% CI]:<br>0.35 [-0.16, 0.86] I-squared: 76.5%
		CBT-I Individual: 238<br><br>placebo, sleep hygiene, wait-list control, psychological, CBT-I self-help: 160	7	multi-dimensional fatigue inventory, fatigue severity scale, chronic respiratory disease questionnaire-fatigue scale, piper fatigue scale	Cohen's *d* [95% CI]:<br>**0.45 [0.07, 0.83]** I-squared: 76.5%
		CBT-I Self-help: 665<br><br>sleep hygiene, wait-list control, pharmacological: 433	7	fatigue severity scale, multi-dimensional fatigue inventory, daytime fatigue scale, multidimensional fatigue symptoms inventory-short form	Cohen's *d* [95% CI]:<br>0.36 [-0.15, 0.88]<br>I-squared: 76.5%
Tang, 2015<br>Meta-analysis	Moderate	CBT-I - psychoeducation, sleep hygiene, stimulus control, sleep restriction, cognitive therapy, and relaxation: 380<br><br>waitlist, treatment as usual; sleep hygiene advice; healthy eating control; nutrition control: 341	6	multidimensional fatigue inventory (MFI); fatigue symptom inventory (FSI); piper fatigue scale (PFS); general fatigue scale (GFS); multidimensional fatigue symptom inventory-short form (MFSI-SF);	Standardized mean difference [95% CI]:<br>**0.38 [0.08, 0.69]** I-squared: 71%
Dickerson, 2014<br>Systematic Review	High	CBT (unspecified): 12<br><br>No comparator (pre- post-intervention)	1	NR	Change in outcome:<br>Pre-/post scores (95% CI) **-0.82 [-1.87, -0.16]**<br>Fatigue improved by week 8

Author, Year<br>Synthesis type	AMSTAR rating	Intervention and sample size	# of studies	Measurement method	Pooled Estimates (MA) or<br>Narrative Results (from SR with no MA)
Howell, 2014<br>Systematic Review	Moderate	Multi-component CBT - sleep hygiene, sleep restriction, stimulus control, cognitive restructuring, and relaxation therapies<br><br>usual care, wait-list control, healthy eating and nutrition, sleep education and hygiene only, no treatment<br><br>Total sample: 178	3	Sleep diary	Fatigue had **significant improvement** in all studies post CBT intervention
Yang, 2014<br>Meta-analysis	Moderate	CBT + relaxation - CBT; sleep hygiene; relaxation CD: 50<br><br>Sleep hygiene education; treatment as usual: 48	2	subjective fatigue questionnaire	Standardized mean difference [95% CI]:<br>0.77 [0.36, 1.18] I-squared: 0%
Health-related Quality of Life					
Brooks, 2014<br>Systematic Review	Low	CBT-I: 7<br><br>No comparator (pre- post-intervention)	1	daily sleep diaries, ISI	Improvements in quality of life measure (p-values not reported)
Dickerson, 2014<br>Systematic Review	High	CBT (unspecified)<br><br>usual treatment; waitlist crossover; waitlist control; control; usual treatment<br><br>Total sample: 72	1	FACT-B	Change in outcome:<br>CBT vs placebo 0.37 [-0.11 to 0.83]
		CBT (unspecified): 10<br><br>No comparator (pre- post-intervention)	1	Global QOL	Change in outcome:<br>-1.09 [-1.98 to -.011]<br>Increase in global and cognitive dimensions of quality of life at 8 weeks follow-up
Ishak, 2012<br>Systematic Review	Low	CBT (unspecified)<br><br>Placebo; no treatment; unspecified; usual care<br><br>Total sample: 706	4	SF-36; CIS-20; GHQ; PANAS; FACT-G	Change in outcome (pre-post ES):<br>physical HRQoL: 0.739; 0.739;<br>mental HRQoL: 0.739; 0.082<br>**Significant improvement** in physical, emotional and mental health QoL was found in all studies

Author, Year<br>Synthesis type	AMSTAR rating	Intervention and sample size	# of studies	Measurement method	Pooled Estimates (MA) or<br>Narrative Results (from SR with no MA)
Howell, 2014<br>Systematic Review	Moderate	Multi-component CBT - sleep hygiene, sleep restriction, stimulus control, cognitive restructuring, and relaxation therapies<br><br>usual care, wait-list control, healthy eating and nutrition, sleep education and hygiene only, no treatment<br><br>Total sample: 81	1	NR	**Significant improvement** since baseline after CBT intervention (p-values not reported)
Ishak, 2012<br>Systematic Review	Low	CBT (individual): psychoeducation, sleep hygiene, stimulus control, sleep restriction, relaxation exercises, cognitive restructuring<br><br>CBT (group): psychoeducation, sleep hygiene, stimulus control, sleep restriction, relaxation exercises, cognitive restructuring<br><br>Total sample: 58	1	SF-36 SIP	Both groups demonstrated **significant improvement** in quality of life compared to baseline **(p=0.025)**

Table E9. Behavioural Interventions

**Author, Year**<br>**Synthesis type**	**AMSTAR rating**	**Intervention and sample size**	**# of studies**	**Measurement method**	**Pooled Estimates (MA) or**<br>**Narrative Results (from SR with no MA)**
Sleep onset latency					
Brasure, 2015<br>Meta-analysis	High	Sleep restriction: 68<br><br>wait-list control, no treatment, or sleep hygiene/sleep education: 73	2	subjective report	Mean difference [95% CI]:<br>-11.38 [-27.74, 4.99] I-squared: 87%
Miller, 2014<br>Systematic Review	High	Sleep restriction therapy: 98<br><br>wait-list control; sleep hygiene instructions: 94	4	sleep diary; actigraphy	Change in outcome (effect size; SD):<br>*intervention [pre-post]:* -19.34 minutes (0.64; 0.37);<br>*control [pre-post]*: -3.64 minutes (0.06; 0.36)<br><br>Sleep restriction arm: sleep latency decreased in all studies, the weighted effect size was medium (0.64)
Buscemi, 2005<br>Meta-analysis	High	Relaxation Training (autogenic, breathing process, EMG feedback); Relaxation exercises (group, hypnotic, progressive): 199<br><br>Placebo: 185	13	sleep diary	Mean difference [95% CI]:<br>-14.56 [-29.33, 0.20] I-squared: 96.1%
Brasure, 2015<br>Meta-analysis	High	Multicomponent Behavioral Interventions or Brief Behavioral Therapy: 70<br><br>Information control or placebo: 76	3	subjective report	Mean difference [95% CI]:<br>**-10.43 [-16.31, -4.55]** I-squared: 0%

Wang, 2005<br>Systematic Review	Moderate	Multifactor intervention: stimulus control, relaxation response, sleep education<br><br>Stimulus control<br><br>Total sample: 18	1	sleep diary	Change in outcome (p-value):<br>Multifactor intervention: **77.3→17.5mins (p<0.001);** stimulus control: **74.9→28mins (p<0.001)**<br><br>Patients achieving SOL <u><</u>20 mins:<br>Multifactor: **6/9**; Stimulus control: **2/9** **(p<0.05 between groups)**<br><br>Both groups had a statistically and clinically significant change in mean sleep onset latency; significantly greater proportion of patients receiving multifactor intervention achieved 'good sleeper status' (SOL <u><</u> 20 mins)
Total Sleep Time					
Brasure, 2015<br>Meta-analysis	High	Sleep restriction: 68<br><br>wait-list control, no treatment, or sleep hygiene/sleep education: 73	2	subjective report	Mean difference [95% CI]:<br>-17.57 [-102.36, 67.21] I-squared: 93%
McCurry, 2007<br>Systematic Review	Critically Low	Sleep restriction (nap sleep restriction therapy; sleep compression; sleep compression guidance and sleep hygiene)<br><br>placebo; waiting list<br>Total sample : 55	1	actigraphy	Sleep restriction **more effective** than either nap restriction or control on actigraphic total sleep time
Miller, 2014<br>Systematic Review	High	Sleep restriction therapy: 98<br><br>wait-list control; sleep hygiene instructions: 94	4	sleep diary; actigraphy	Change in outcome<br>[effect size (SD)]: intervention [pre-post] 17.06 minutes (0.30; 0.31);<br>control [pre-post]<br><br>6.13 minutes (0.01; 0.40) Secondary pre-to-post measures of sleep diary variables were also compared at post-treatment to baseline levels. This revealed a small non-significant increase in total sleep time (ES=0.3)
Brasure, 2015<br>Meta-analysis	High	Relaxation Therapy: 39<br><br>passive control: 38	2	subjective report	Mean difference [95% CI]:<br>10.23 [-19.64, 40.11] I-squared: 29%
Wake after sleep onset					

Miller, 2014<br>Systematic Review	High	Sleep restriction therapy: 82<br><br>wait-list control; sleep hygiene instructions: 78	3	sleep diary<br>actigraphy	Change in outcome [effect size (SD)]:<br>intervention [pre-post]: -42.17 min, [1.36 (0.42)]<br>control: [pre-post] -11.30 min [0.01 (0.55)]<br>Reductions for wake after sleep onset were found in three studies; the weighted effect size in the intervention was large (1.36)
Buscemi, 2005<br>Meta-analysis	High	Relaxation Training (autogenic, breathing process, EMG feedback); Relaxation exercises (group, hypnotic, progressive): 60<br><br>Placebo: 57	3	sleep diary	Mean difference [95% CI]:<br>-1.61 [-14.05, 10.82] I-squared: 20%
Brasure, 2015<br>Meta-analysis	High	Multicomponent Behavioral Interventions or Brief Behavioral Therapy: 70 patients<br><br>Information control or placebo: 76 patients	3	subjective report	Mean difference [95% CI]:<br>**-14.9 [-22.66, -7.14]** I-squared: 0%
Sleep Quality					
Hwang, 2016<br>Meta-analysis	Critically Low	Behavioural therapy; Brief behavioral treatment: NR<br><br>Control (unspecified): NR	5	PSQI	Standardized mean difference [95% CI]:<br>**1.90 [0.04, 2.94]** I-squared: 96.27%
Bogdanov, 2017<br>Systematic Review	Low	Problem solving therapy<br>sleep education only<br><br>Total sample: 356	1	PSQI	**Significant improvement** compared to control at 6-month but not 12-month follow-up<br>**(p=0.003, 6 months; p=0.88, 12 months)**
Miller, 2014<br>Systematic Review	High	Sleep restriction therapy: 44<br>wait-list control; sleep hygiene instructions: 50	1	sleep diary	Change in outcome (effect size; SD):<br>intervention [pre-post] 2.77→2.90 (0.3; NA);<br>control [pre-post]: 2.57→2.58 (0.03; NA)<br>Sleep quality ratings were only reported in one study and were found to increase (ES=0.3)
Brooks, 2014<br>Systematic Review	Low	Progressive relaxation training: 37<br>Control: NR	1	sleep diary	Treatment group had **significant difference** in sleep quality pre- and post-treatment
Hellström, 2011<br>Systematic Review	Moderate	mental imagery<br>usual care<br>Total sample: 36	1	VSH-sleep scale	Effects of relaxation on sleep quality were small and did not reach significance

Tamrat, 2013<br>Systematic Review	Low	Relaxation techniques; audiotape guided imagery; relaxation tapes<br>usual care<br>solitary activity; baseline<br>Total sample: 211	3	self-rating (poor, fair, good); Richards Campbell Sleep Questionnaire (RCSQ)	In summary, there is low strength of evidence that studies of relaxation techniques improve sleep quality
Seda, 2015<br>Meta-analysis	Low	Imagery rehearsal therapy (IRT): NR<br><br>CBT (stimulus control and sleep restriction therapy) + Imagery rehearsal therapy (IRT): NR	8	PSQI	Cohen’s *d* (95% CI):<br>IRT post-treatment: 0.50 (0.16 to 0.84)<br>IT+CBT post-treatment: 1.32 (0.68 to 1.96)<br>Q-statistic (p-value): 4.75 (p=0.03)
Sleep Satisfaction					
McCurry, 2007<br>Systematic Review	Critically Low	Sleep restriction (nap sleep restriction therapy; sleep compression; sleep compression guidance and sleep hygiene)<br><br>placebo; waiting list<br>Total sample: 125	1	NR	sleep compression guidance in combination with sleep education delivered via a standardized video resulted in greater post-test sleep satisfaction scores compared to placebo
Sleep Efficiency					
McCurry, 2007<br>Systematic Review	Critically Low	Sleep restriction (nap sleep restriction therapy; sleep compression; sleep compression guidance and sleep hygiene)<br><br>placebo; waiting list<br>Total sample: 129	2	sleep logs	Sleep restriction therapy was found to be **significantly more beneficial** than a sleep hygiene/placebo control
Miller, 2014<br>Systematic Review	High	sleep restriction therapy: 82<br><br>wait-list control; sleep hygiene instructions: 78	3	sleep diary<br>actigraphy	Change in outcome (effect size; SD):<br>intervention [pre-post]: 16.28% (1.50; 0.35);<br>Control [pre-post]: 4.59% (0.04; 0.23)<br>Sleep efficiency increased in three studies; the effect size for sleep efficiency the intervention was large
Fatigue Severity					
Ballesio, 2017<br>Meta-analysis	High	BT Group: 24<br><br>Placebo: 50	2	fatigue severity scale (2)	Cohen’s d [95% CI]:<br>0.09 [-0.61 to 0.79] I-squared: 76.5%

Table E10. Mindfulness

Author, Year<br>Synthesis type	AMSTAR rating	Intervention and sample size	# of studies	Measurement method	Pooled Estimates (MA) or<br>Narrative Results (from SR with no MA)
Sleep onset latency					
Gong, 2016<br>Meta-analysis	Low	mindfulness based stress reduction; mindfulness meditation; mindfulness based therapy for insomnia<br><br>wait list control; sleep hygiene education; self-monitoring condition<br>Total sample: 83	2	sleep diary	standardized mean difference [95% CI]:<br>**-0.53 [-0.97, -0.09]** I-squared: 0%
Total Sleep Time					
Gong, 2016<br>Meta-analysis	Low	mindfulness based stress reduction; mindfulness meditation; mindfulness based therapy for insomnia<br><br>wait list control; sleep hygiene education; self-monitoring condition<br>Total sample: 58	2	sleep diary	standardized mean difference [95% CI]:<br>0.28 [-0.24, 0.80] I-squared: 0%
Sleep Quality					
Gong, 2016<br>Meta-analysis	Low	mindfulness based stress reduction; mindfulness meditation; mindfulness based therapy for insomnia<br><br>wait list control; sleep hygiene education; self-monitoring condition<br>Total sample: 83	2	sleep diary	standardized mean difference [95% CI]:<br>**0.68 [0.24, 1.13]** I-squared: 0%
		Total sample: 109	2	PSQI	standardized mean difference [95% CI]:<br>-1.09 [-1.50, 0.69] I-squared: 0%

Venables, 2014<br>Systematic Review	Critically Low	mindfulness based stress reduction; mind-body bridging; mindfulness meditation: 63<br>No comparator<br>(pre- post- intervention)	1	PSQI	Proportion of respondents: [pre-intervention]<br>91% score >5; 51% score >10<br>[post-intervention]<br>79% score >5; 27% score >10
Sleep efficiency					
Gong, 2016<br>Meta-analysis	Low	mindfulness based stress reduction; mindfulness meditation; mindfulness based therapy for insomnia<br><br>wait list control; sleep hygiene education; self-monitoring condition<br>Total sample: 58	2	sleep diary	standardized mean difference [95% CI]:<br>0.85 [-0.31, 1.40] I-squared: 0%
Venables, 2014<br>Systematic Review	Critically Low	mindfulness based stress reduction; mind-body bridging; mindfulness meditation: 205<br>No comparator<br>(pre- post- intervention)	3	NR	2 trials showed **significant improvement** in SE; 1 trial showed no significant improvement

Table E11. Combination Therapy

Author, Year<br>Synthesis type	AMSTAR rating	Intervention and sample size	# of studies	Measurement method	Pooled Estimates (MA) or<br>Narrative Results (from SR with no MA)
Sleep onset latency					
Chiesa, 2009<br>Systematic Review	Low	Pharmacotherapy (general) and Mindfulness based cognitive therapy; mindfulness based stress reduction: 14<br>No comparator (pre- post- intervention)	1	NR	Median sleep latency reduced from 30 minutes to 26 minutes per night
Total Sleep Time					
Buscemi, 2005<br>Meta-analysis	High	Triazolam; temazepam and CBT: 27Placebo: 25	2	sleep diary	Mean difference [95% CI]:<br>23.2 [-2.3, 48.8] I-squared: 0%
Chiesa, 2009<br>Systematic Review	Low	Pharmacotherapy (general) and Mindfulness based cognitive therapy; mindfulness based stress reduction: 30<br>No comparator (pre- post- intervention)	2	NR	1 trial showed and average increase of 1 hour total sleep time; 1 trial showed **significant improvement** in measures of sleep quantity that persisted at 6 and 12 months
Sleep Quality					
Chiesa, 2009<br>Systematic Review	Low	Pharmacotherapy (general) and Mindfulness based cognitive therapy; mindfulness based stress reduction: 14<br>No comparator (pre- post- intervention)	1	NR	Measures of sleep quality **improved significantly** post-treatment, improvements were maintained at 6 and 12 months; levels of mindfulness were shown to correlate with quality of sleep

Appendix F: Tables of primary studies by treatment comparison for outcomes with more than one included SR or SR+MA

Table F1. Primary studies across included systematic reviews that examined Benzodiazepines

Primary Studies	Buscemi, 2005* n = 18	Sateia, 2017* n = 2	Soldatos, 1999* n = 28[1]	Kolla, 2011 n = 1	Swainston Harrison, 2005 n = 1	Times Cited	Outcome(s)
**Flurazepam (n=10)**							
Campbell, 1987	+	--	--	--	--	1	SOL
Cohn, 1991	+	--	--	--	--	1	SOL
Fillingim, 1982	+	--	--	--	--	1	SOL
Fleming, 1995	+	--	--	--	--	1	SOL
Hartmann, 1983	+	--	--	--	--	1	SOL
Mamelak, 1987	+	--	--	--	--	1	SOL
Mamelak, 1989	+	--	--	--	--	1	SOL
Mello de Paula, 1984	+	--	--	--	--	1	SOL
Mitler, 1984	+	--	--	--	--		SOL
Scharf, 1990	+	--	--	--	--	1	SOL
**Temazepam (n=8)**							
Beary, 1984	+	--	--	--	--	1	SOL
Fillingim, 1982	+	--	--	--	--	1	SOL
Glass, 2008	--	+	--	--	--	1	SOL, TST, SQ
Hindmarch, 1979	--	+	--	--	--	1	SQ
Leppik, 1997	+	--	--	--	--	1	SOL
Tuk, 1997	+	--	--	--	--	1	SOL, WASO
Morin, 1999	+	--	--	--	--	1	WASO
Wu, 2006	--	+	--	--	--	1	SOL, TST
**Triazolam (n=38)**							
Adam, 1984	--	--	+	--	--	--	SOL, TST
Bergougnan, 1992	--	--	+	--	--	--	SOL, TST
Borberly and Achermann, 1991	--	--	+	--	--	--	SOL, TST
Bowen, 1978	+	--	--	--	--	1	SOL
Cluydts, 1986	--	--	+	--	--	--	SOL, TST
Cohn, 1983	+	--	--	--	--	1	SOL
Drake(1), 2000	+	--	--	--	--	1	SOL
Drake(2), 2000	+	--	--	--	--	1	SOL

Fabre, 1997	--	--	--	+	--	1	TST
Kales 1976	--	--	+	--	--	--	SOL, TST
Kales, 1986	--	--	+	--	--	--	SOL, TST
Kales, 1991	--	--	+	--	--	--	SOL, TST
Kanno, 1993	--	--	+	--	--	--	SOL, TST
Leppik, 1997	+	--	--	--	--	1	SOL
Mamelak, 1984	--	--	+	--	--	--	SOL, TST
Mamelak, 1990	--	--	+	--	--	--	SOL, TST
Merlotti, 1988	--	--	+	--	--	--	SOL, TST
Mitler, 1984	+	--	+	--	--	1	SOL, TST, WASO
Monti, 1994	--	--	+	--	+	--	SOL, TST
Mouret, 1990	--	--	+	--		--	SOL, TST
Nicholson and Stone, 1980	--	--	+	--	--	--	SOL, TST
Nicholson, 1982	--	--	+	--	--	--	SOL, TST
Ogura, 1980	--	--	+	--	--	--	SOL, TST
Okuma and Honda, 1978	--	--	+	--	--	--	SOL, TST
Pegram, 1980	--	--	+	--	--	--	SOL, TST
Roehrs, 1992	--	--	+	--	--	--	SOL, TST
Roth, 1974	--	--	+	--	--	--	SOL, TST
Roth, 1976	--	--	+	--	--	--	SOL, TST
Roth, 1977	--	--	+	--	--	--	SOL, TST
Saletu, 1994	--	--	+	--	--	--	SOL, TST
Scharf, 1990	+		+	--	--	1	SOL, TST
Spinweber and Johnson, 1982	--	--	+	--	--	--	SOL, TST
Steens, 1993	+	--	--	--	--	1	SOL, WASO
Stepanski, 1982	--	--	+	--	--	--	SOL, TST
Tiberge, 1988	--	--	+	--	--	--	SOL, TST
Vogel, 1975	--	--	+	--	--	--	SOL, TST
Walsh, 1998	+	--	--	--	--	1	SOL
Ware, 1997	--	--	+	--	--	--	SOL, TST

* Systematic review with meta-analysis

[1]The authors of the Soldatos, 1999 paper did not clearly report which studies were included in the analyses for each outcome. All of the primary studies in this review are listed here but are not included in times cited count.

Table F2. Primary studies across included systematic reviews that examined non-benzodiazepines

Primary Studies	Brasure, 2015* n = 8	Buscemi, 2005* n = 22	Sateia, 2017* n = 12	Soldatos, 1999*[1] n = 34	Mayers, 2005 n = 1	Mendelson, 2005 n = 1	Swainston Harrison, 2005 n = 2	Ishak, 2012 n = 3	Cimolai, 2007 n = 4	Times Cited	Outcome(s)
**Zolpidem v Placebo (n = 46)**											
Allain, 2001	--	+	--	--	--	--	--	--	--	1	SOL, WASO
Asnis, 1999	--	+	--	--	--	--	--	--	--	1	SOL, WASO
Benoit, 1992	--	--	--	+	--	--	--	--	--	--	SOL, TST
Bergougnan, 1992	--	--	--	+	--	--	--	--	--	--	SOL, TST
Besset, 1995	--	--	--	+	--	--	--	--	--	--	SOL, TST
Brunner, 1991	--	--	--	+	--	--	--	--	--	--	SOL, TST
De Roeck and Cluydts, 1991	--	--	--	+	--	--	--	--	--	--	SOL, TST
Declerck, 1992	--	--	--	+	--	--	--	--	--	--	SOL. TST
Declerck, 1999	--	+	--	--	--	--	--	--	--	1	SOL, WASO
Dorsey, 2004	--	--	+	--	--	--	--	--	--	1	SOL, WASO
Dujardin, 1998	--	+	--	--	--	--	--	--	--	1	SOL
Elie, 1999	+	+	+	--	--	--	--	--	--	3	SOL, TST, WASO, SQ
Erman, 2008	--	--	+	--	--	--	--	--	--	1	SOL, TST, WASO, SQ
Fleming, 1995	--	+	--	--	--	--	--	--	--	1	SOL
Fry, 2000	+	+	--	--	--	--	--	--	--	2	SOL, SQ
Hajak, 2002	--	--	--	--	--	--	--	+	--	1	QoL
Hermann, 1988	--	--	--	+	--	--	--	--	--	--	SOL, TST
Hermann, 1993	--	+	+	+	--	--	--	--	--	2	SOL, TST, WASO, SE
Jacobs, 2004	+	--	+	--	--	--	--	--	--	2	SOL, TST
Kanno, 1993b	--	--	--	+	--	--	--	--	--	--	SOL, TST
Koshorec, 1988	--	--	--	+	--	--	--	--	--	--	SOL, TST
Kryger, 1991	--	--	--	+	--	--	--	--	--	--	SOL, TST
Lahmeyer, 1997	+	+	--	--	--	--	--	--	--	2	SOL, SQ
Leppik, 1997	--	+	--	--	--	--	--	--	--	1	SOL
Merlotti, 1989	--	--	--	+	--	--	--	--	--	--	SOL, TST

Primary Studies	**Brasure, 2015* n = 8**	**Buscemi, 2005* n = 22**	**Sateia, 2017* n = 12**	**Soldatos, 1999*[1] n = 34**	**Mayers, 2005 n = 1**	**Mendelson, 2005 n = 1**	**Swainston Harrison, 2005 n = 2**	**Ishak, 2012 n = 3**	**Cimolai, 2007 n = 4**	**Times Cited**	**Outcome(s)**
Monti, 1989	--	--	--	+	--	--	--	--	--	--	SOL, TST
Monti, 1994	--	--	--	--	--	--	+	--	--	1	TST, WASO
Monti, 1996	--	+	--	--	--	--	--	--	--	1	SOL, WASO
Monti, 2000	--	+	--	--	--	--	--	--	--	1	SOL, WASO
Nicholson and Pascoe, 1986	--	--	--	+	--	--	--	--	--	--	SOL, TST
Nobuhara, 1992	--	--	--	+	--	--	--	--	--	--	SOL, TST
Oswald and Adam, 1988	--	--	--	+	--	--	--	--	--	--	SOL, TST
Perlis, 2004	+	--	+	--	--	--	--	--	--	2	SOL, TST, WASO
Randall, 2012	+	--	+	--	--	--	--	--	--	2	SOL, TST, WASO, SQ, SE
Scharf, 1991a	--	--	--	+	--	--	--	--	--	--	SOL, TST
Scharf, 1991b	--	--	--	+	--	--	--	--	--	--	SOL, TST
Scharf, 1991c	--	--	--	+	--	--	--	--	--	--	SOL, TST
Scharf, 1994	+	+	+	+	--	--	--	--	--	3	SOL, TST, WASO, SQ, SE
Staner, 2005	--	--	+	--	--	--	--	--	--	1	SQ
Steens, 1993	--	+	--	--	--	--	--	--	--	1	SOL, WASO
Uchimura, 2012	--	--	+	--	--	--	--	--	--	1	SOL, SQ
Vogel, 1989	--	--	--	+	--	--	--	--	--	--	SOL, TST
Walsh, 1998	--	+	+	--	+	+	--	--	--	4	SOL, TST, WASO, SQ
Walsh, 2000	--	+	--	--	--	--	--	--	--	1	SOL
Walsh, 2002	+	+	--	--	--	--	--	--	--	2	SOL
Ware, 1997	--	--	+	+	--	--	+	--	--	2	SOL, TST, WASO, SE
**Zolpidem v Triazolam (n = 3)**											
Monti, 1994	--	--	--	--	--	--	+	--	--	1	TST,

Primary Studies	**Brasure, 2015* n = 8**	**Buscemi, 2005* n = 22**	**Sateia, 2017* n = 12**	**Soldatos, 1999*[1] n = 34**	**Mayers, 2005 n = 1**	**Mendelson, 2005 n = 1**	**Swainston Harrison, 2005 n = 2**	**Ishak, 2012 n = 3**	**Cimolai, 2007 n = 4**	**Times Cited**	**Outcome(s)**
											WASO
Silvestri, 1996	--	--	--	--	--	--	+	--	--	1	SOL, WASO, SE
Ware, 1997	--	--	--	--	--	--	+	--	--	1	WASO, SE
**Zolpidem (nightly) v Zolpidem (as needed) (n = 1)**											
Hajak, 2002	--	--	--	--	--	--	--	+	--	1	QoL
**Zopiclone v Placebo (n = 21)**											
Billiard, 1989	--	--	--	+	--	--	--	--	--	--	SOL, TST
Campbell, 1987	--	+	--	--	--	--	--	--	--	1	SOL
Chaudoir, 1983	--	+	--	--	--	--	--	--	--	1	SOL
Fleming, 1988	--	--	--	+	--	--	--	--	--	--	SOL, TST
Godtlibsen and Dreyfus, 1980	--	--	--	+	--	--	--	--	--	--	SOL, TST
Goldenberg, 1994	--	--	--	--	--	--	--	+	--	1	QoL
Jobert, 1993	--	--	--	+	--	--	--	--	--	--	SOL, TST
Kim, 1993	--	--	--	+	--	--	--	--	--	--	SOL, TST
Lamphere, 1989	--	+	--	+	--	--	--	--	--	1	SOL, TST
Leger, 1995	--	--	--	--	--	--	--	+	--	1	QoL
Lemoine, 1995	--	--	--	--	--	--	--	--	+	1	A/D/D
Mamelak, 1982	--	--	--	+	--	--	--	--	+	1	SOL, TST, A/D/D
Mamelak, 1987	--	+	--	--	--	--	--	--	--	1	SOL
Monchesky, 1986	--	+	--	--	--	--	--	--	--	1	SOL
Mouret, 1990	--	--	--	+	--	--	--	--	--	--	SOL, TST
Nicholson and Stone, 1982	--	--	--	+	--	--	--	--	--	--	SOL, TST
Nicholson and Stone, 1987	--	--	--	+	--	--	--	--	--	--	SOL, TST
Pecknold, 1990	--	--	--	--	--	--	--	--	+	1	A/D/D
Petre-Quadens ,1982	--	--	--	+	--	--	--	--	--	--	SOL, TST
Ponciano, 1990	--	--	--	--	--	--	--	--	+	1	Hangover
Tiberge, 1988	--	--	--	+	--	--	--	--	--	--	SOL, TST

[1]The authors of the Soldatos, 1999 paper did not clearly report which studies were included in the analyses for each outcome. All of the primary studies in this review are listed here and are not included in times cited count.
*systematic review with meta-analysis

Table F3. Primary studies across included systematic reviews that examined Suvorexant

Primary Studies (n = 3)	**Kishi, 2015* n = 3**	**Kuriyama, 2017* n = 3[2]**	**Brasure, 2015* n = 2**	**Citrome, 2014* n = 3**	**Times Cited**	Outcome(s)
Michelson, 2014	+	†	--	--	1	SOL, TST, WASO, ISI, Hangover, Injury, A/D/D
Herring, 2012	--	†	--	--	--	SOL, TST, WASO, ISI, Hangover, Injury, A/D/D
Herring, 2014[1] Suvorexant; 15 and 20mg Suvorexant; 30 and 40mg	+	†	+	+	3	SOL, TST, WASO, ISI, Hangover, Injury, A/D/D

[1]This publication includes two trials

[2]only able to ascertain that these three trials were the only ones included in the SR+MA, unable to determine which outcome analyses they contributed to thus they are not counted in the final column

*systematic review with meta-analysis

Table F4. Primary studies across included systematic reviews that examined antidepressants

Primary Studies	**Brasure, 2015* n = 2**	**Buscemi, 2005* n = 5**	**Sateia, 2017* n = 5**	**Yuan, 2010* n = 4**	**Liu, 2017* n = 6**	**Kolla, 2011 n = 1**	**Mayers, 2005 n = 3**	**Vande Griend, 2012 n = 9**	**Yeung, 2015 n = NR[1]**	**Mendelson, 2005 n = 8**	**Times Cited**	**Outcome(s)**
					**Doxepin v Placebo (n = 12)**							
Goldberg, 1974	--	--	--	--	--	--	--	--	+	--	1	SOL
Hajak, 1996	--	+	--	--	+	--	+	--	--	--	3	SOL, TST, SQ
Hajak, 2000	--	--	--	--	+	--	--	--	--	--	1	TST
Hajak, 2001	--	+	--	+	--	--	+	+	+	--	5	SOL, TST, WASO, SQ, SE
Krystal, 2010	+	--	+	--	+	--	--	+	--	--	4	SOL, TST, WASO, SQ, SE
Krystal, 2011	--	--	+	--	+	--	--	+	--	--	3	SOL, TST, WASO, SE

Primary Studies	**Brasure, 2015* n = 2**	**Buscemi, 2005* n = 5**	**Sateia, 2017* n = 5**	**Yuan, 2010* n = 4**	**Liu, 2017* n = 6**	**Kolla, 2011 n = 1**	**Mayers, 2005 n = 3**	**Vande Griend, 2012 n = 9**	**Yeung, 2015 n = NR[1]**	**Mendelson, 2005 n = 8**	**Times Cited**	**Outcome(s)**
Lankford, 2011	+	--	+	--	--	--	--	+	--	--	3	SOL, TST, WASO
Lankford, 2012	--	--	+	--	--	--	--	--	--	--	1	SQ
Rodenbeck, 2003	--	+	--	+	--	--	--	--	--	--	2	SOL, TST, WASO, SE
Roth, 2007	--	--	+	+	+	--	--	+	--	--	4	SOL, TST, WASO, SE
Roth, 2010	--	--	--	--	--	--	--	+	--	--	1	SOL, TST, WASO, SE
Scharf, 2008	--	--	+	+	+	--	--	+	--	--	4	SOL, TST, WASO, SQ, SE
**Trazodone v Placebo (n = 20)**												
Kaynak, 2004	--	--	--	--	--	--	--	--	--	+	1	TST, SQ, SE
Blacker, 1988	--	--	--	--	--	--	--	--	--	+	1	SQ
Davey, 1988	--	--	--	--	--	--	--	--	--	+	1	SQ
Friedmann, 2008	--	--	--	--	--	+	--	--	--	--	1	SQ
Haffmans and Vos, 1999	--	+	--	--	--	--	--	--	--	+	2	SOL, TST
Le Bon, 2003	--	--	--	--	--	+	--	+	--	--	2	SOL, TST, WASO, SE
Mashiko, 1999	--	--	--	--	--	--	+	--	--	--	1	TST
Montgomery, 1983	--	--	--	--	--	--	--	--	--	+	1	SOL, TST, SQ
Moon, 1998	--	--	--	--	--	--	--	--	--	+	1	SQ
Mouret, 1988	--	--	--	--	--	--	--	--	--	+	1	SOL, TST
Nierenberg, 1994	--	--	--	--	--	--	+	--	--	--	1	SOL, TST, SQ
Parrino, 1994	--	--	--	--	--	--	--	--	--	+	1	SOL, TST, WASO, SE
Roth, 2011	--	--	--	--	--	--	--	+	--	--	1	SOL, WASO
Saletu-Zyhlarz, 2001	--	--	--	--	--	--	--	--	--	+	1	SOL, TST, SE

Primary Studies	**Brasure, 2015* n = 2**	**Buscemi, 2005* n = 5**	**Sateia, 2017* n = 5**	**Yuan, 2010* n = 4**	**Liu, 2017* n = 6**	**Kolla, 2011 n = 1**	**Mayers, 2005 n = 3**	**Vande Griend, 2012 n = 9**	**Yeung, 2015 n = NR[1]**	**Mendelson, 2005 n = 8**	**Times Cited**	**Outcome(s)**
Saletu-Zyhlarz, 2002	--	--	--	--	--	--	--	--	--	+	1	TST, SE
Scharf and Sachais, 1990	--	--	--	--	--	--	--	--	--	+	1	SOL, TST, SE
Scharf, 1990	--	--	--	--	--	--	--	--	--	+	1	WASO
Stein, 2011	--	--	--	--	--	--	--	+	--	--	1	TST, SE
Van Bemmel, 1992	--	--	--	--	--	--	--	--	--	+	1	SOL, TST, SE
Walsh, 1998	--	+	--	--	--	--	+	+	--	+	4	SOL, TST, WASO, SQ
**Trazodone v Zolpidem (n = 1)**												
Walsh, 1998	--	--	--	--	--	--	+	+	--	+	3	SOL, TST, WASO

[1] Unable to determine some or all of the primary studies associated with this review
*systematic review with meta-analysis

Table F5. Primary studies across included systematic reviews that examined anti-psychotics

Primary Studies	**Anderson, 2014 n = 7**	**Coe, 2014 n = 2**	**Kolla, 2011 n = 1**	**Wine, 2009 n = 3**	**Times Cited**	**Outcome(s)**
**Quetiapine (n = 9)**						
Baune, 2007	+	--	--	--	1	SQ
Endicott, 2008	+	--	--	--	1	SQ
Juri, 2005	+	--	--	+	2	SOL, SQ
Martinotti, 2008	--	--	+	--	1	SQ
Robert, 2005	--	--	--	+	1	TST, SQ
Tassniyom, 2010	+	+	--	--	2	SOL, TST
Terán, 2008	+	--	--	--	1	SOL, SQ
Todder, 2006	+	--	--	--	1	SOL, TST, SQ, SE
Wiegand, 2008	+	+	--	+	3	SOL, TST, SQ, SE

Table F6. Primary studies across included systematic reviews that examined melatonin

Primary Studies	**Buscemi, 2004* n = 12[1]**	**Buscemi, 2005* n = 8**	**Ferracioli-Oda, 2013* n = 8**	**McCleery, 2016* n = 2**	**Lee, NA* n = 15**	**Sateia, 2017* n = 3**	**Xu, 2015* n = 8**	**Zhang, 2016* n = 4**	**Bellon, 2006 n = 13**	**Chase, 1997 n = 2**	**Costello, 2014 n = 1**	**Culpepper, 2015 n = 3**	**Vural, 2014 n = 1**	**Times Cited**	**Outcome(s)**
Almeida, 2003	--	--	--	--	+	--	--	--	+	--	--	+	--	3	SOL, TST, SQ, SE
Andrade, 2001	+	--	--	--	--	--	--	--	--	--	--	--	--	1	SOL, WASO
Arendt 1986a and 1986b	--	--	--	--	--	--	--	--	--	+	--	--	--	1	SQ
Asaya, 2003	--	--	--	--	--	--	+	+	--	--	--	--	--	2	TST
Baskett, 2003	+	--	--	--	+	--	--	--	--	--	--	+	--	3	SOL, TST, SQ, SE
Dahlitz, 1991	+	--	+	--	--	--	--	--	+	+	--	--	--	4	SOL, TST
Dawson, 1998	+	+	+	--	+	--	--	--	--	--	--	--	--	4	SOL, TST, WASO, SQ, SE
Dowling, 2005	--	--	--	--	--	--	--	+	--	--	--	--	--	1	WASO
Dowling, 2008	--	--	--	+	--	--	+	+	--	--	--	--	--	3	TST
Eckerberg, 2012	--	--	--	--	+	--	--	--	--	--	--	--	--	1	TST
Ellis, 1995	--	--	--	--	+	--	--	--	--	--	--	--	--	1	TST, SQ, SE
Ellis, 1996	+	+	--	--	--	--	--	--	+	--	--	--	--	3	SOL, TST, WASO, SQ, SE
Garfunkel, 1995	+	+	--	--	+	--	--	--	--	+	+	--	--	5	SOL, TST, WASO, SQ, SE
Garfunkel, 1997	--	+	--	--	+	--	--	--	--	--	--	--	--	2	SOL, WASO
Garzon, 2009	--	--	+	--	+	--	--	--	--	--	--	+	--	3	SQ
Gehman, 2009	--	--	--	--	--	--	+	+	--	--	--	--	--	2	TST, WASO, SE
Gooneratne, 2012	--	--	--	--	--	--	--	--	--	--	--	--	+	1	SQ
Haimov, 1995	+	+	+	--	+	--	--	--	--	--	--	--	--	4	SOL, WASO, SQ, SE
He, 2005	--	--	--	--	+	--	--	--	--	--	--	--	--	1	SOL, TST, SE
Hughes, 1998	--	--	--	--	+	--	--	--	--	--	--	--	+	2	SOL, TST, SQ, Se
James, 1989	--	--	+	--	--	--	--	--	--	--	--	--	--	1	TST

Primary Studies	**Buscemi, 2004* n = 12[1]**	**Buscemi, 2005* n = 8**	**Ferracioli-Oda, 2013* n = 8**	**McCleery, 2016* n = 2**	**Lee, NA* n = 15**	**Sateia, 2017* n = 3**	**Xu, 2015* n = 8**	**Zhang, 2016* n = 4**	**Bellon, 2006 n = 13**	**Chase, 1997 n = 2**	**Costello, 2014 n = 1**	**Culpepper, 2015 n = 3**	**Vural, 2014 n = 1**	**Times Cited**	**Outcome(s)**
James, 1990	+	+	+	--	+	--	--	--	+	+	+	--	--	7	SOL, TST, WASO, SQ, SE
Kayumov, 2001	+	--	+	--	--	--	--	--	+	--	--	--	--	3	SOL, TST, SQ, SE
Kunz, 2010	--	--	+	--	--	--	--	--	--	--	--	--	--	1	SOL, TST, SQ
Lemoine, 2007	--	--	+	--	+	+	--	--	--	--	--	+	--	4	SQ
Luthringer, 2009	--	--	+	--	+	+	--	--	--	--	--	--	--	3	SOL, TST, SQ
MacFarlane, 1991	--	--	--	--	--	--	--	--	+	--	--	--	--	1	TST
Medeiros, 2007	--	--	--	--	--	--	--	+	--	--	--	--	--	1	TST, SE
Montes, 2002	--	--	+	--	--	--	--	--	--	--	--	--	--	1	TST
Montes, 2003	+	+	--	--	--	--	--	--	--	--	--	--	--	2	SOL, WASO, SQ, SE
Mundey, 2005	--	--	+	--	--	--	--	--	--	--	--	--	--	1	TST, SQ
Nagtegaal, 1995	--	--	+	--	--	--	--	--	--	--	--	--	--	1	SOL
Nagtegaal, 1998	--	--	--	--	--	--	--	--	+	--	--	--	--	1	SOL, TST, SQ, SE
Riemersma, 2008a and 2008b	--	--	--	--	--	--	+	--	--	--	--	--	--	1	TST, SE
Serfaty, 2002	--	--	--	--	--	--	+	+	--	--	--	--	--	2	TST, SE
Singer, 2003a and 2003b	--	--	--	+	--	--	+	+	--	--	--	--	--	3	TST, WASO, SQ, SE
Smiths, 2001	--	--	--	--	+	--	--	--	--	--	--	--	--	1	SOL, TST
Smiths, 2003	+	--	--	--	+	--	--	--	--	--	--	--	--	2	SOL, TST
Sugaya, 2007	--	--	--	--		--	--	--	--	--	--	--	+	1	QoL
Van Geijlswijk, 2011	--	--	--	--	+	--	--	--	--	--	--	--	--	1	SOL
Wade, 2007	--	--	+	+	+	+	--	--	--	--	--	+	--	5	SOL, SQ
Wade, 2011	--	--	+	--	+	--	--	--	--	--	--	--	--	2	SOL, TST, SQ
Wade, 2014	--	--	--	--	--	--	--	+	--	--	--	--	--	1	WASO
Zhdanova, 2001	+	+	+	--	+	--	--	--	--	--	--	--	--	4	SOL, TST,

Primary Studies	**Buscemi, 2004* n = 12[1]**	**Buscemi, 2005* n = 8**	**Ferracioli-Oda, 2013* n = 8**	**McCleery, 2016* n = 2**	**Lee, NA* n = 15**	**Sateia, 2017* n = 3**	**Xu, 2015* n = 8**	**Zhang, 2016* n = 4**	**Bellon, 2006 n = 13**	**Chase, 1997 n = 2**	**Costello, 2014 n = 1**	**Culpepper, 2015 n = 3**	**Vural, 2014 n = 1**	**Times Cited**	**Outcome(s)**
															WASO, SQ, SE

[1] Unable to determine some of the primary studies associated with this review
*systematic review with meta-analysis

Table F7. Primary studies across included systematic reviews that compared diphenhydramine to inactive controls

Primary Studies	**Sateia, 2017* n = 2**	**Culpepper, 2015 n = 3**	**Vande Griend, 2012 n = 4**	**Times Cited**	**Outcome(s)**
Glass, 2008	+	+	+	3	SOL, TST, SQ, SE
Morin, 2005	+	+	+	3	SOL, TST, SE, ISI
Katayose, 2012	--	+	--	1	SOL
Rickels, 1983	--	--	+	1	SOL, TST
Meuleman, 1987	--	--	+	1	SOL, TST, WASO

*systematic review with meta-analysis

Table F8. Primary studies across included systematic reviews that compared cognitive behavioural interventions to inactive controls

Primary Studies (n = 89)	Brasure, 2015* n = 21	Cheng, 2012* n = 4	Ho, 2016* n = 4	Koffel, 2015* n = 6	Navarro-Bravo, 2015* n=7	van Straten, 2007* n=10[2]	Ye, 2016* n = 15	Yang, 2014* n = 1	Buscemi, 2005* n = 13[2]	Ho, 2015* n = 8[2]	Johnson, 2016* n = 7	Trauer, 2015* n = 16	Montgomery, 2003* n = 3	Irwin, 2006* n = 20	van Straten, 2009* n = 8[2]	Seyffert, 2016* n = 7	Okajima, 2011* n = 9[2]	Zachariae, 2016* n = 10	Howell, 2014 n = 3	McCurry, 2007 n = 1	Brooks, 2014 n = 1	Ishak, 2012 n = 1	Dickerson, 2014 n = 1	Venables, 2014 n = 8	Times Cited
**Sleep Onset Latency**																									
Altena, 2008	--	--	--	--	--	--	--	--	--	--	--	+	--	--	--	--	--	--	--	--	--	--	--	--	1
Arnedt, 2013	+	--	--	--	--	--	--	--	--	--	--	--	--	--	--	--	--	--	--	--	--	--	--	--	1
Ascher, 1979	--	--	--	--	--	--	--	--	+	--	--	--	--	--	--	--	--	--	--	--	--	--	--	--	1
Berger, 2009	--	--	--	--	--	--	--	--	--	--	--	--	--	--	--	--	--	--	--	--	--	--	+	--	1
Blom, 2015a	--	--	--	--	--	--	+	--	--	--	--	--	--	--	--	--	--	--	--	--	--	--	--	--	1
Blom, 2015b	--	--	--	--	--	--	+	--	--	--	--	--	--	--	--	--	--	--	--	--	--	--	--	--	1
Bothelius, 2013	+	--	--	--	--	--	--	--	--	--	--	+	--	--	--	--	--	--	--	--	--	--	--	--	2
Buysse, 2011	--	--	--	--	--	--	--	--	--	--	--	+	--	--	--	--	--	--	--	--	--	--	--	--	1
Carr-Kaffashan, 1979	--	--	--	--	--	--	--	--	--	--	--	--	--	+	--	--	--	--	--	--	--	--	--	--	1
Chen, 2008	--	--	--	--	--	--	--	+	--	--	--	--	--	--	--	--	--	--	--	--	--	--	--	--	1
Chen, 2011	--	--	--	--	+	--	--	--	--	--	--	--	--	--	--	--	--	--	--	--	--	--	--	--	1
Currie, 2000	--	--	--	+	+	--	--	--	--	--	--	--	--	--	--	--	--	--	--	--	--	--	--	--	2
Currie, 2014	--	--	--	--	--	--	--	--	--	--	--	--	--	--	--	--	--	--	--	--	+	--	--	--	1

Dirksen, 2008	--	--	--	--	--	--	--	--	--	--	--	--	--	--	--	--	--	--	--	--	--	--	--	+	1
Dixon, 2006	--	--	--	--	--	--	--	--	--	--	--	--	--	--	--	--	--	--	--	--	--	+	--	--	1
Edinger and Sampson, 2003	--	--	--	--	--	--	--	--	--	--	--	+	--	--	--	--	--	--	--	--	--	--	--	--	1
Edinger, 2003	+	--	--	--	--	--	--	--	+	--	--	--	--	+	--	--	--	--	--	--	--	--	--	--	3
Edinger, 2007	--	--	--	--	--	--	--	--	--	--	--	+	--	--	--	--	--	--	--	--	--	--	--	--	1
Edinger, 2009	+	--	--	--	--	--	--	--	--	--	--	+	--	--	--	--	--	--	--	--	--	--	--	--	2
Epstein and Dirksen, 2007	--	--	--	+	+	--	--	--	--	--	+	--	--	--	--	--	--	--	--	--	--	--	--	+	4
Epstein, 2012	--	--	--	--	+	--	--	--	--	--	--	--	--	--	--	--	--	--	--	--	--	--	--	--	1
Espie, 1989	--	--	--	--	--	--	--	--	+	--	--	--	--	+	--	--	--	--	--	--	--	--	--	--	2
Espie, 2001	--	--	--	--	--	--	--	--	--	--	--	+	--	+	--	--	--	--	--	--	--	--	--	--	3
Espie, 2006	--	--	--	--	--	--	--	--	--	--	--	--	--	--	--	--	--	--	--	--	--	--	+	+	2
Espie, 2007	+	--	--	+	+	--	--	--	--	--		+	--	--	--	--	--	--	--	--	--	--	--	--	4
Espie, 2008a	--	--	--	--	--	--	--	--	--	--	+	--	--	--	--	--	--	--	+	--	--	--	+	+	4
Espie, 2008b	--	--	--	--	--	--	--	--	--	--	--	--	--	--	--	--	--	--	--	--	--	--	--	+	1
Espie, 2012	+	--	--	--	--	--	+	--	--	--	--	--	--	--	--	+	--	+	--	--	--	--	--	--	4
Fiorentino, 2008	--	--	--	--	--	--	--	--	--	--	--	--	--	--	--	--	--	--	--	--	--	--	+	--	1
Garland, 2014	--	--	--	--	--	--	--	--	--	--	+	--	--	--	--	--	--	--	--	--	--	--	--	--	1

Harvey, 2003	--	--	--	--	--	--	--	--	+	--	--	--	--	--	--	--	--	--	--	--	--	--	--	--	1
Ho, 2014	--	--	--	--	--	--	+	--	--	--	--	--	--	--	--	+	--	+	--	--	--	--	--	--	3
Holqvist, 2014	--	--	--	--	--	--	+	--	--	--	--	--	--	--	--	--	--	--	--	--	--	--	--	--	1
Irwin, 2014	+	--	--	--	--	--	--	--	--	--	--	--	--	--	--	--	--	--	--	--	--	--	--	--	1
Jacobs, 2004	+	--	--	--	--	--	--	--	--	--	--	+	--	--	--	--	--	--	--	--	--	--	--	--	2
Jernelov, 2012	+	--	--	--	--	--	--	--	--	--	--	--	--	--	--	--	--	--	--	--	--	--	--	--	1
Jungquist, 2010	+	--	--	--	--	--	--	--	--	--	--	--	--	--	--	--	--	--	--	--	--	--	--	--	1
Kaldo, 2015	--	--	--	--	--	--	+	--	--	--	--	--	--	--	--	--	--	--	--	--	--	--	--	--	1
Lacks, 1983b	--	--	--	--	--	--	--	--	+	--	--	--	--	+	--	--	--	--	--	--	--	--	--	--	2
Lancee, 2012	--	--	--	--	--	--	+	--	--	--	--	--	--	--	--	+	--	+	--	--	--	--	--	--	3
Lancee, 2013a	--	--	--	--	--	--	+	--	--	--	--	--	--	--	--	--	--	--	--	--	--	--	--	--	1
Lancee, 2013b	--	--	--	--	--	--	+	--	--	--	--	--	--	--	--	--	--	--	--	--	--	--	--	--	1
Lancee, 2015	--	--	--	--	--	--	+	--	--	--	--	--	--	--	--	--	--	--	--	--	--	--	--	--	1
Lichstein, 2001	--	--	--	--	--	--	--	--	+	--	--	--	+	+	--	--	--	--	--	--	--	--	--	--	3
Lick, 1977	--	--	--	--	--	--	--	--	--	--	--	--	--	+	--	--	--	--	--	--	--	--	--	--	1
Lovato, 2014	--	--	--	--	--	--	--	--	--	--	--	+	--	--	--	--	--	--	--	--	--	--	--	--	1
Mack, 2013	--	--	+	--	--	--	--	--	--	--	--	--	--	--	--	--	--	--	--	--	--	--	--	--	1
Margolies, 2013	--	--	+	--	--	--	--	--	--	--	--	--	--	--	--	--	--	--	--	--	--	--	--	--	1
Matthews, 2010	--	--	--	--	--	--	--	--	--	--	--	--	--	--	--	--	--	--	--	--	--	--	+	--	1
Matthews, 2014	--	--	--	--	--	--	--	--	--	--	+	--	--	--	--	--	--	--	--	--	--	--	--	--	1

McCrae, 2007	--	--	--	--	--	--	--	--	--	--	--	+	--	--	--	--	--	--	--	--	--	--	--	--	1
Mimeault, 1999	+	--	--	--	--	--	--	--	--	--	--	--	--	--	--	--	--	--	--	--	--	--	--	--	1
Morawetz, 1989	--	--	--	--	--	--	--	--	--	--	--	--	--	+	--	--	--	--	--	--	--	--	--	--	1
Morin, 1988	--	--	--	--	--	--	--	--	--	--	--	--	+	+	--	--	--	--	--	--	--	--	--	--	2
Morin, 1993	+	--	--	+	--	--	--	--	--	--	--	+	+	+	--	--	--	--	--	--	--	--	--	--	5
Nicassio, 1974	--	--	--	--	--	--	--	--	--	--	--	--	--	+	--	--	--	--	--	--	--	--	--	--	1
Nicassio, 1982	--	--	--	--	--	--	--	--	--	--	--	--	--	+	--	--	--	--	--	--	--	--	--	--	1
Pallesen, 2003	--	--	--	--	--	--	--	--	--	--	--	--	--	+	--	--	--	--	--	--	--	--	--	--	1
Pillai, 2015	--	--	--	--	--	--	--	--	--	--	--	--	--	--	--	--	--	+	--	--	--	--	--	--	1
Puder, 1983	--	--	--	--	--	--	--	--	--	--	--	--	--	+	--	--	--	--	--	--	--	--	--	--	1
Quesnel, 2003	--	--	--	--	--	--	--	--	--	--	--	--	--	--	--	--	--	--	--	--	--	--	+	+	2
Raymond, 2010	--	--	--	--	--	--	--	--	--	--	--	--	--	--	--	--	--	--	--	--	--	--	--	+	1
Riedel, 1995	--	--	--	--	--	--	--	--	--	--	--	--	--	+	--	--	--	--	--	--	--	--	--	--	1
Ritterband, 2009	+	+	--	--	--	--	+	--	--	--	--	--	--	--	--	+	--	+	--	--	--	--	--	--	5
Ritterband, 2012	--	+	--	--	--	--	+	--	--	--	+	--	--	--	--	--	--	+	+	--	--	--	--	--	5
Rybarczyk, 2002	--	--	--	+	--	--	--	--	--	--	--	--	--	+	--	--	--	--	--	--	--	--	--	--	2
Rybarczyk, 2005	+	--	--	--	+	--	--	--	--	--	--	--	--	--	--	--	--	--	--	+	--	--	--	--	3
Sanavio, 1990	--	--	--	--	--	--	--	--	+	--	--	--	--	--	--	--	--	--	--	--	--	--	--	--	1
Savard, 2005	--	--	--	--	+	--	--	--	--	--	+	--	--	--	--	--	--	--	+	--	--	--	--	+	4

Savard, 2014[1]	--	--	--	--	--	--	--	--	--	--	+	--	--	--	--	--	--	--	--	--	--	--	--	--	1
Smith, 2015	+	--	--	--	--	--	--	--	--	--	--	--	--	--	--	--	--	--	--	--	--	--	--	--	1
Soeffing, 2008	--	--	--	--	--	--	--	--	--	--	--	+	--	--	--	--	--	--	--	--	--	--	--	--	1
Stanton, 1989	--	--	--	--	--	--	--	--	+	--	--	--	--	+	--	--	--	--	--	--	--	--	--	--	2
Strom, 2004	+	+	--	--	--	--	+	--	--	--	--	--	--	--	--	+	--	+	--	--	--	--	--	--	5
Suzuki, 2008	--	--	--	--	--	--	--	--	--	--	--	--	--	--	--	--	--	+	--	--	--	--	--	--	1
Talbot, 2014	--	--	+	--	--	--	--	--	--	--	--	--	--	--	--	--	--	--	--	--	--	--	--	--	1
Tang, 2012	+	--	--	--	--	--	--	--	--	--	--	--	--	--	--	--	--	--	--	--	--	--	--	--	1
Taylor, 2014	--	--	--	--	--	--	--	--	--	--	--	+	--	--	--	--	--	--	--	--	--	--	--	--	1
Thiart, 2015	--	--	--	--	--	--	--	--	--	--	--	--	--	--	--	--	--	--	--	--	--	--	--	--	
Turner, 1979	--	--	--	--	--	--	--	--	--	--	--	--	--	+	--	--	--	--	--	--	--	--	--	--	1
Turner, 1982	--	--	--	--	--	--	--	--	--	--	--	--	--	+	--	--	--	--	--	--	--	--	--	--	1
Ulmer, 2011	--	--	+	--	--	--	--	--	--	--	--	--	--	--	--	--	--	--	--	--	--	--	--	--	1
Van Straten, 2009	+	--	--	--	--	--	--	--	--	--	--	--	--	--	--	--	--	--	--	--	--	--	--	--	1
Van Straten, 2014	+	--	--	--	--	--	+	--	--	--	--	--	--	--	--	+	--	+	--	--	--	--	--	--	4
Vincent, 2009	+	+	--	--	--	--	+	--	--	--	--	--	--	--	--	+	--	+	--	--	--	--	--	--	5
Vitiello, 2009	--	--	--	+	--	--	--	--	--	--	--	--	--	--	--	--	--	--	--	--	--	--	--	--	1
Waters, 2003	--	--	--	--	--	--	--	--	+	--	--	+	--	--	--	--	--	--	--	--	--	--	--	--	2

Woolfolk, 1983	--	--	--	--	--	--	--	--	--	--	--	--	--	+	--	--	--	--	--	--	--	--	--	--	1
Wu, 2006	+	--	--	--	--	--	--	--	--	--	--	+	--	--	--	--	--	--	--	--	--	--	--	--	2

Primary Studies n = 72	**Brasure, 2015* n = 15**	**Cheng, 2012* n = 4**	**Ho, 2016* n = 4**	**Koffel, 2015* n = 6**	**Navarro-Bravo, 2015* n = 8**	**van Straten, 2007* n=10[2]**	**Ye, 2016* n = 15**	**Dickerson, 2014 n = 2**	**Venables, 2014 n = 8**	**Ho, 2015* n = 8[2]**	**Irwin, 2006* n = 15**	**Montgomery, 2003* n = 4**	**Okajima, 2011* n = 9[2]**	**Seyffert, 2016* n = 8**	**Trauer, 2015* n = 16**	**van Straten, 2009* n=8[2]**	**Zachariae, 2016* n = 10**	**Howell, 2014 n = 2**	**Wang, 2005 n = 2**	**Times Cited**
**Total Sleep Time**																				
Arnedt, 2013	+	--	--	--	--	--	--	--	--	--	--	--	--	--	--	--	--	--	--	1
Barsevick, 2010	--	--	--	--	--	--	--	--	--	--	--	--	--	--	--	--	--	+	--	1
Berger, 2009	--	--	--	--	--	--	--	--	+	--	--	--	--	--	--	--	--	--	--	1
Blom, 2015a	--	--	--	--	--	--	+	--	--	--	--	--	--	--	--	--	--	--	--	1
Blom, 2015b	--	--	--	--	--	--	+	--	--	--	--	--	--	--	--	--	--	--	--	1
Bothelius, 2013	--	--	--	--	--	--	--	--	--	--	--	--	--	--	+	--	--	--	--	1
Buysse, 2011	--	--	--	--	--	--	--	--	--	--	--	--	--	--	+	--	--	--	--	1
Chen, 2001	--	--	--		+	--	--	--	--	--	--	--	--	--	--	--	--	--	--	1
Currie, 2000	--	--	--	+	+	--	--	--	--	--	--	--	--	--	--	--	--	--	--	2
Davidson, 2001	--	--	--	--	--	--	--	+	--	--	--	--	--	--	--	--	--	--	--	1
Dirksen, 2008	--	--	--	--	--	--	--	--	+	--	--	--	--	--	--	--	--	--	--	1
Edinger and Sampson, 2003	--	--	--	--	--	--	--	--	--	--	--	--	--	--	+	--	--	--	--	1
Edinger, 2001	+	--	--	--	--	--	--	--	--	--	+	--	--	--	+	--	--	--	+	4
Edinger, 2003	+	--	--	--	--	--	--	--	--	--	+	--	--	--	--	--	--	--	--	2
Edinger, 2007	--	--	--	--	--	--	--	--	--	--	--	--	--	--	+	--	--	--	--	1
Edinger, 2009	+	--	--	--	--	--	--	--	--	--	--	--	--	--	+	--	--	--	--	2
Epstein and Dirksen, 2007	--	--	--	+	+	--	--	--	+	--	--	--	--	--	--	--	--	--	--	3
Epstein, 2012	--	--	--	--	+	--	--	--	--	--	--	--	--	--	--	--	--	--	--	1
Espie, 2001	--	--	--	--	--	--	--	--	--	--	+	--	--	--	--	--	--	--	--	1
Espie, 2003	--	--	--	--	--	--	--	--	--	--	+	--	--	--	--	--	--	--	--	1
Espie, 2006	--	--	--	--	--	--	--	--	+	--	--	--	--	--	--	--	--	--	--	1
Espie, 2007	+	--	--	+	+	--	--	--	--	--	--	--	--	--	+	--	--	--	--	4
Espie, 2008a	--	--	--	--	--	--	--	+	+	--	--	--	--	--	--	--	--	--	--	2
Espie, 2008b	--	--	--	--	--	--	--	--	+	--	--	--	--	--	--	--	--	--	--	1
Espie, 2012	+	--	--	--	--	--	+	--	--	--	--	--	--	+	--	--	+	--	--	4

Fiorentino, 2008	--	--	--	--	--	--	--	--	+	--	--	--	--	--	--	--	--	--	--	1
Ho, 2014	--	--	--	--	--	--	+	--	--	--	--	--	--	+	--	--	+	--	--	3
Holmqvist, 2014	--	--	--	--	--	--	+	--	--	--	--	--	--	--	--	--	--	--	--	1
Jacobs, 2004	+	--	--	--	--	--	--	--	--	--	--	--	--	--	+	--	--	--	--	2
Jernelov, 2012	+	--	--	--	--	--	--	--	--	--	--	--	--	--	--	--	--	--	--	1
Kaldo, 2015	--	--	--	--	--	--	+	--	--	--	--	--	--	+	--	--	--	--	--	2
Lancee, 2012	--	--	--	--	--	--	+	--	--	--	--	--	--	+	--	--	+	--	--	3
Lancee, 2013a	--	--	--	--	--	--	+	--	--	--	--	--	--	--	--	--	--	--	--	1
Lancee, 2013b	--	--	--	--	--	--	+	--	--	--	--	--	--	--	--	--	--	--	--	1
Lancee, 2015	--	--	--	--	--	--	+	--	--	--	--	--	--	--	--	--	--	--	--	1
Lichstein, 2001	--	--	--	--	--	--	--	--	--	--	+	+	--	--	--	--	--	--	--	2
Lick, 1977	--	--	--	--	--	--	--	--	--	--	+	--	--	--	--	--	--	--	--	1
Lovato, 2014	--	--	--	--	--	--	--	--	--	--	--	--	--	--	+	--	--	--	--	1
Mack, 2013	--	--	+	--	--	--	--	--	--	--	--	--	--	--	--	--	--	--	--	1
Margolies, 2013	--	--	+	--	--	--	--	--	--	--	--	--	--	--	--	--	--	--	--	1
Matthews, 2010	--	--	--	--	--	--	--	--	+	--	--	--	--	--	--	--	--	--	--	1
McCrae, 2007	--	--	--	--	--	--	--	--	--	--	--	--	--	--	+	--	--	--	--	1
Mimeault, 1999	+	--	--	--	--	--	--	--	--	--	--	--	--	--	--	--	--	--	+	2
Morawetz, 1989	--	--	--	--	--	--	--	--	--	--	+	--	--	--	--	--	--	--	--	1
Morin, 1988	--	--	--	--	--	--	--	--	--	--	+	+	--	--	--	--	--	--	--	2
Morin, 1993	--	--	--	+	--	--	--	--	--	--	+	+	--	--	+	--	--	--	--	4
Morin, 1999	--	--	--	--	+	--	--	--	--	--	+	+	--	--	+	--	--	--	--	4
Pallesen, 2003	--	--	--	--	--	--	--	--	--	--	+	--	--	--	--	--	--	--	--	1
Pillai, 2015	--	--	--	--	--	--	--	--	--	--	--	--	--	--	--	--	+	--	--	1
Quesnel, 2003	--	--	--	--	--	--	--	--	+	--	--	--	--	--	--	--	--	--	--	1
Raymond, 2010	--	--	--	--	--	--	--	--	+	--	--	--	--	--	--	--	--	--	--	1
Riedel, 1995	--	--	--	--	--	--	--	--	--	--	+	--	--	--	--	--	--	--	--	1
Ritterband, 2009	+	+	--	--	--	--	+	--	--	--	--	--	--	+	--	--	+	--	--	5
Ritterband, 2012	--	+	--	--	--	--	+	--	--	--	--	--	--	--	--	--	+	--	--	3
Rybarczyk, 2002	--	--	--	+	--	--	--	--	--	--	+	--	--	--	--	--	--	--	--	2
Rybarczyk, 2005	--	--	--	--	+	--	--	--	--	--	--	--	--	--	--	--	--	--	--	1
Savard, 2005	--	--	--	--	+	--	--	--	+	--	--	--	--	--	--	--	--	+	--	3
Savard, 2013	--	--	--	--	--	--	--	--	+	--	--	--	--	--	--	--	--	--	--	1
Sivertsen, 2006	--	--	--	--	--	--	--	--	--	--	--	--	--	--	+	--	--	--	--	1
Soeffing, 2008	--	--	--	--	--	--	--	--	--	--	--	--	--	--	+	--	--	--	--	1
Strom, 2004	+	+	--	--	--	--	+	--	--	--	--	--	--	+	--	--	+	--	--	5
Suzuki, 2008	--	--	--	--	--	--	--	--	--	--	--	--	--	--	--	--	+	--	--	1

Talbot, 2014	--	--	+	--	--	--	--	--	--	--	--	--	--	--	--	--	--	--	--	1
Taylor, 2014	--	--	--	--	--	--	--	--	--	--	--	--	--	--	+	--	--	--	--	1
Turner, 1979	--	--	--	--	--	--	--	--	--	--	+	--	--	--	--	--	--	--	--	1
Turner, 1982	--	--	--	--	--	--	--	--	--	--	+	--	--	--	--	--	--	--	--	1
Ulmer, 2011	--	--	+	--	--	--	--	--	--	--	--	--	--	--	--	--	--	--	--	1
Van Straten, 2009	+	--	--	--	--	--	--	--	--	--	--	--	--	--	--	--	--	--	--	1
Van Straten, 2014	+	--	--	--	--	--	+	--	--	--	--	--	--	+	--	--	+	--	--	4
Vincent, 2009	+	+	--	--	--	--	+	--	--	--	--	--	--	+	--	--	+	--	--	5
Vitiello, 2009	--	--	--	+	--	--	--	--	--	--	--	--	--	--	--	--	--	--	--	1
Wu, 2006	+	--	--	--	--	--	--	--	--	--	--	--	--	--	+	--	--	--	--	2

Primary Studies n = 70	**Brasure, 2015* n = 11**	**Cheng, 2012* n = 4**	**Ho, 2016* n = 4**	**Koffel, 2015* n = 6**	**Navarro-Bravo, 2015* n = 7**	**van Straten, 2007*[1] n = 10[2]**	**Ye, 2016* n = 11**	**Brooks, 2014 n = 1**	**Dickerson, 2014 n = 1**	**Venables, 2014 n = 8**	**Wang, 2005 n = 3**	**Buscemi, 2005* n =10[2]**	**Ho, 2015* n = 6[2]**	**Irwin, 2006* n = 15**	**Johnson, 2016* n = 7**	**Okajima, 2011* n = 6[2]**	**Trauer, 2015* n = 14**	**Montgomery, 2003* n = 4**	**Seyffert, 2016* n = 6**	**van Straten, 2009* n = 6[2]**	**Zachariae, 2016* n = 7**	**Howell, 2014 n = 2**	**McCurry, 2007 n = 1**	**Times Cited**
**Wake After Sleep Onset**																								
Arnedt, 2013	+	--	--	--	--	--	--	--	--	--	--	--	--	--	--	--	--	--	--	--	--	--	--	1
Berger, 2009	--	--	--	--	--	--	--	--	--	+	--	--	--	--	--	--	--	--	--	--	--	--	--	1
Bothelius, 2013	+	--	--	--	--	--	--	--	--	--	--	--	--	--	--	--	+	--	--	--	--	--	--	2
Buysse, 2011	--	--	--	--	--	--	--	--	--	--	--	--	--	--	--	--	+	--	--	--	--	--	--	1
Currie, 2000	--	--	--	+	+	--	--	--	--	--	--	--	--	--	--	--	--	--	--	--	--	--	--	2
Currie, 2004	--	--	--	--	--	--	--	+	--	--	--	--	--	--	--	--	--	--	--	--	--	--	--	1
Davies, 1986	--	--	--	--	--	--	--	--	--	--	--	+	--	--	--	--	--	+	--	--	--	--	--	2
Dirksen, 2008	--	--	--	--	--	--	--	--	--	+	--	--	--	--	--	--	--	--	--	--	--	--	--	1

Edinger and Sampson, 2003	--	--	--	--	--	--	--	--	--	--	+	--	--	--	--	--	+	--	--	--	--	--	--	2
Edinger, 2001	--	--	--	--	--	--	--	--	--	--	+	+	--	+	--	--	+	--	--	--	--	--	--	4
Edinger, 2003	+	--	--	--	--	--	--	--	--	--	--	+	--	+	--	--	--	--	--	--	--	--	--	3
Edinger, 2007	--	--	--	--	--	--	--	--	--	--	--	--	--	--	--	--	+	--	--	--	--	--	--	1
Edinger, 2009	+	--	--	--	--	--	--	--	--	--	--	--	--	--	--	--	+	--	--	--	--	--	--	2
Epstein and Dirksen, 2007	--	--	--	+	+	--	--	--	--	+	--	--	--	--	+	--	--	--	--	--	--	--	--	4
Espie, 2001	--	--	--	--	--	--	--	--	--	--	+	--	--	+	--	--	--	--	--	--	--	--	--	2
Espie, 2006	--	--	--	--	--	--	--	--	--	+	--	--	--	--	--	--	--	--	--	--	--	--	--	1
Espie, 2007	+	--	--	+	+	--	--	--	--	--	--	--	--	--	--	--	+	--	--	--	--	--	--	4
Espie, 2008a	--	--	--	--	--	--	--	--	+	+	--	--	--	--	+	--	--	--	--	--	--	+	--	4
Espie, 2008b	--	--	--	--	--	--	--	--	--	+	--	--	--	--	--	--	--	--	--	--	--	--	--	1
Espie, 2012	+	--	--	--	--	--	+	--	--	--	--	--	--	--	--	--	--	--	+	--	+	--	--	4
Esptein, 2012	--	--	--	--	+	--	--	--	--	--	--	--	--	--	--	--	--	--	--	--	--	--	--	1
Fiorentino, 2008	--	--	--	--	--	--	--	--	--	+	--	--	--	--	--	--	--	--	--	--	--	--	--	1
Garland, 2014	--	--	--	--	--	--	--	--	--	--	--	--	--	--	+	--	--	--	--	--	--	--	--	1
Ho, 2014	--	--	--	--	--	--	+	--	--	--	--	--	--	--	--	--	--	--	+	--	+	--	--	3
Hoelscher and Edinger, 1988	--	--	--	--	--	--	--	--	--	--	--	--	--	--	--	--	--	--	--	--	--	--	+	1

Holmqvist, 2014	--	--	--	--	--	--	+	--	--	--	--	--	--	--	--	--	--	--	--	--	--	--	--	1
Irwin, 2014	+	--	--	--	--	--	--	--	--	--	--	--	--	--	--	--	--	--	--	--	--	--	--	1
Jernelov, 2012	+	--	--	--	--	--	--	--	--	--	--	--	--	--	--	--	--	--	--	--	--	--	--	1
Jungquist, 2010	+	--	--	--	--	--	--	--	--	--	--	--	--	--	--	--	--	--	--	--	--	--	--	1
Lacks, 1983	--	--	--	--	--	--	--	--	--	--	--	+	--	+	--	--	--	--	--	--	--	--	--	2
Lancee, 2012	--	--	--	--	--	--	+	--	--	--	--	--	--	--	--	--	--	--	+	--	+	--	--	3
Lancee, 2013a	--	--	--	--	--	--	+	--	--	--	--	--	--	--	--	--	--	--	--	--	--	--	--	1
Lancee, 2013b	--	--	--	--	--	--	+	--	--	--	--	--	--	--	--	--	--	--	--	--	--	--	--	1
Lancee, 2015	--	--	--	--	--	--	+	--	--	--	--	--	--	--	--	--	--	--	--	--	--	--	--	1
Lichstein, 2001	--	--	--	--	--	--	--	--	--	--	--	+	--	+	--	--	--	+	--	--	--	--	--	3
Lick, 1977	--	--	--	--	--	--	--	--	--	--	--	--	--	+	--	--	--	--	--	--	--	--	--	1
Lovato, 2014	--	--	--	--	--	--	--	--	--	--	--	--	--	--	--	--	+	--	--	--	--	--	--	1
Mack, 2013	--	--	+	--	--	--	--	--	--	--	--	--	--	--	--	--	--	--	--	--	--	--	--	1
Margolies, 2013	--	--	+	--	--	--	--	--	--	--	--	--	--	--	--	--	--	--	--	--	--	--	--	1
Matthews, 2010	--	--	--	--	--	--	--	--	--	+	--	--	--	--	--	--	--	--	--	--	--	--	--	1
Matthews, 2014	--	--	--	--	--	--	--	--	--	--	--	--	--	--	+	--	--	--	--	--	--	--	--	1
McCrae, 2007	--	--	--	--	--	--	--	--	--	--	--	--	--	--	--	--	+	--	--	--	--	--	--	1
Mimeault, 1999	+	--	--	--	--	--	--	--	--	--	--	--	--	--	--	--	--	--	--	--	--	--	--	1
Morawetz, 1989	--	--	--	--	--	--	--	--	--	--	--	--	--	+	--	--	--	--	--	--	--	--	--	1
Morin, 1988	--	--	--	--	--	--	--	--	--	--	--	--	--	+	--	--	--	--	--	--	--	--	--	1

Morin, 1993	+	--	--	+	--	--	--	--	--	--	--	--	--	+	--	--	+	+	--	--	--	--	+	6
Morin, 1999	+	--	--	--	+	--	--	--	--	--	--	+	--	+	--	--	+	+	--	--	--	--	--	6
Pallesen, 2003	--	--	--	--	--	--	--	--	--	--	--	--	--	+	--	--	--	--	--	--	--	--	--	1
Quesnel, 2003	--	--	--	--	--	--	--	--	--	+	--	--	--	--	--	--	--	--	--	--	--	--	--	1
Raymond, 2010	--	--	--	--	--	--	--	--	--	+	--	--	--	--	--	--	--	--	--	--	--	--	--	1
Riedel, 1995	--	--	--	--	--	--	--	--	--	--	--	--	--	+	--	--	--	--	--	--	--	--	--	1
Ritterband, 2009	+	+	--	--	--	--	+	--	--	--	--	--	--	--	--	--	--	--	+	--	+	--	--	5
Ritterband, 2012	--	+	--	--	--	--	+	--	--	--	--	--	--	--	+	--	--	--	--	--	+	--	--	4
Rybarczyk, 2002	--	--	--	+	--	--	--	--	--	--	--	--	--	+	--	--	--	--	--	--	--	--	+	3
Rybarczyk, 2005	+	--	--	--	+	--	--	--	--	--	--	--	--	--	--	--	--	--	--	--	--	--	+	3
Sanavio, 1990	--	--	--	--	--	--	--	--	--	--	--	+	--	--	--	--	--	--	--	--	--	--	--	1
Savard, 2005	--	--	--	--	+	--	--	--	--	+	--	--	--	--	+	--	--	--	--	--	--	+	--	4
Savard, 2014[1]	--	--	--	--	--	--	--	--	--	--	--	--	--	--	+	--	--	--	--	--	--	--	--	1
Smith, 2015	+	--	--	--	--	--	--	--	--	--	--	--	--	--	--	--	--	--	--	--	--	--	--	1
Soeffing, 2008	--	--	--	--	--	--	--	--	--	--	--	--	--	--	--	--	+	--	--	--	--	--	--	1
Strom, 2004	+	+	--	--	--	--	+	--	--	--	--	--	--	--	--	--	--	--	+	--	+	--	--	5
Talbot, 2014	--	--	+	--	--	--	--	--	--	--	--	--	--	--	--	--	--	--	--	--	--	--	--	1
Tang, 2012	+	--	--	--	--	--	--	--	--	--	--	--	--	--	--	--	--	--	--	--	--	--	--	1
Taylor, 2014	--	--	--	--	--	--	--	--	--	--	--	--	--	--	--	--	+	--	--	--	--	--	--	1
Turner, 1979	--	--	--	--	--	--	--	--	--	--	--	--	--	+	--	--	--	--	--	--	--	--	--	1

Turner, 1982	--	--	--	--	--	--	--	--	--	--	--	--	--	+	--	--	--	--	--	--	--	--	--	1
Ulmer, 2011	--	--	+	--	--	--	--	--	--	--	--	--	--	--	--	--	--	--	--	--	--	--	--	1
Vincent, 2009	+	+	--	--	--	--	+	--	--	--	--	--	--	--	--	--	--	--	+	--	+	--	--	5
Vitiello, 2009	--	--	--	+	--	--	--	--	--	--	--	--	--	--	--	--	--	--	--	--	--	--	--	1
Waters, 2003	--	--	--	--	--	--	--	--	--	--	--	+	--	--	--	--	+	--	--	--	--	--	--	2

Primary Studies n = 47	**Brasure, 2015* n= 6**	**Ho, 2016* n = 6**	**Koffel, 2015* n = 5**	**Navarro-Bravo, 2015* n = 5**	**van Straten, 2007* n = 10[2]**	**Cheng, 2012* n = 4**	**Bogdanov, 2017 n = 1**	**Venables, 2014 n = 2**	**Buscemi, 2005* n = 2[2]**	**Yang, 2014* n = 3**	**Ho, 2015* n = 4[2]**	**Irwin, 2006* n = 7**	**Okajima, 2011* n = 2[2]**	**Tang, 2015* n = 11**	**van Straten, 2009* n = 7[2]**	**Zachariae, 2016* n = 8**	**Howell, 2014 n = 2**	**McCurry, 2007 n = 4**	**Times Cited**
**Sleep Quality**																			
Arnedt, 2013	+	--	--	--	--	--	--	--	--	--	--	--	--	--	--	--	--	--	1
Barsevick, 2010	--	--	--	--	--	--	--	--	--	--	--	--	--	+	--	--	--	--	1
Berger, 2009	--	--	--	--	--	--	--	+	--	--	--	--	--	+	--	--	+	--	3
Bjorvatn, 2011	+	--	--	--	--	--	--	--	--	--	--	--	--	--	--	--	--	--	1
Chen, 2008	--	--	--	--	--	--	--	--	--	+	--	--	--	--	--	--	--	--	1
Chen, 2011	--	--		+	--	--	--	--	--	+	--	--	--	--	--	--	--	--	2
Currie, 2000	--	--	+	+	--	--	--	--	--	--	--	--	--	+	--	--	--	--	3
Davis, 2007	--	+	--	--	--	--	--	--	--	--	--	--	--	--	--	--	--	--	1
Davis, 2011,	--	+	--	--	--	--	--	--	--	--	--	--	--	--	--	--	--	--	1
Edinger, 2001	--	--	--	--	--	--	--	--	--	--	--	+	--	--	--	--	--	--	1
Edinger, 2003	--	--	--	--	--	--	--	--	--	--	--	+	--	--	--	--	--	--	1
Edinger, 2005	--	--	--	--	--	--	--	--	--	--	--	--	--	+	--	--	--	--	1
Edinger, 2009	+	--	--	--	--	--	--	--	--	--	--	--	--	--	--	--	--	--	1
Epstein and Dirksen, 2007	--	--	+	--	--	--	--	--	--	--	--	--	--	--	--	--	--	--	1
Espie, 2007	+	--	+	+	--	--	--	--	--	--	--	--	--	--	--	--	--	--	3
Espie, 2008	--	--	--	--	--	--	--	--	--	--	--	--	--	+	--	--	--	--	1
Espie, 2012	--	--	--	--	--	--	--	--	--	--	--	--	--	--	--	+	--	--	1
Fiorentino, 2008	--	--	--	--	--	--	--	+	--	--	--	--	--	--	--	--	--	--	1

Fiorentino, 2009	--	--	--	--	--	--	--	--	--	--	--	--	--	--	--	--	+	--	1
Garland, 2014	--	--	--	--	--	--	--	--	--	--	--	--	--	+	--	--	--	--	1
Ho, 2014	--	--	--	--	--	--	--	--	--	--	--	--	--		--	+	--	--	1
Jungquist, 2010	--	--	--	--	--	--	--	--	--	--	--	--	--	+	--	--	--	--	1
Krakow, 2001	--	+	--	--	--	--	--	--	--	--	--	--	--	--	--	--	--	--	1
Lichstein, 2000	--	--	--	--	--	--	--	--	--	--	--	--	--	--	--	--	--	+	1
Lichstein, 2001	--	--	--	--	--	--	--	--	--	--	--	+	--	--	--	--	--	--	1
Lick, 1977	--	--	--	--	--	--	--	--	--	--	--	+	--	--	--	--	--	--	1
Lu, 2016	--	--	--	--	--	--	+	--	--	--	--	--	--	--	--	--	--	--	1
Mack, 2013	--	+	--	--	--	--	--	--	--	--	--	--	--	--	--	--	--	--	1
Margolies, 2013	--	+	--	--	--	--	--	--	--	--	--	--	--	--	--	--	--	--	1
Martinez, 2014	--	--	--	--	--	--	--	--	--	--	--	--	--	+	--	--	--	--	1
McCurry, 1998	--	--	--	--	--	--	--	--	--	--	--	--	--	--	--	--	--	+	1
Mimeault, 1999	+	--	--	--	--	--	--	--	--	--	--	--	--	--	--	--	--	--	1
Miro, 2011	--	--	+	+	--	--	--	--	--	--	--	--	--	+	--	--	--	--	3
Rambod, 2013	--	--	--	--	--	--	--	--	--	+	--	--	--	--	--	--	--	--	1
Riedel, 1995	--	--	--	--	--	--	--	--	--	--	--	+	--	--	--	--	--	--	1
Ritterband, 2009	--	--	--	--	--	+	--	--	--	--	--	--	--	--	--	+	--	--	2
Ritterband, 2012	--	--	--	--	--	+	--	--	--	--	--	--	--	+	--	+	--	--	3
Rybarcyzk, 2002	--	--	+	--	--	--	--	--	--	--	--	--	--	--	--	--	--	+	2
Rybarcyzk, 2005	--	--	--	+	--	--	--	--	--	--	--	--	--	--	--	--	--	+	2
Savard, 2005	--	--	--	--	--	--	--	--	--	--	--	--	--	+	--	--	--	--	1
Strom, 2004	--	--	--	--	--	+	--	--	--	--	--	--	--	--	--	+	--	--	2
Suzuki, 2008	--	--	--	--	--	--	--	--	--	--	--	--	--	--	--	+	--	--	1
Turner, 1979	--	--	--	--	--	--	--	--	--	--	--	+	--	--	--	--	--	--	1
Turner, 1982	--	--	--	--	--	--	--	--	--	--	--	+	--	--	--	--	--	--	1
Ulmer, 2011	--	+	--	--	--	--	--	--	--	--	--	--	--	--	--	--	--	--	1
Van Straten, 2014	+	--	--	--	--	--	--	--	--	--	--	--	--	--	--	+	--	--	2
Vincent, 2009	--	--	--	--	--	+	--	--	--	--	--	--	--	--	--	+	--	--	2

Primary Studies n = 83	Cheng, 2012* n = 4	Ho, 2016* n = 5	Koffel, 2015* n = 6	Navarro-Bravo, 2015* n= 8	van Straten, 2007* n = 10[2]	Ye, 2016* n = 15	Bogdanov, 2017 n = 1	Brooks, 2014 n = 3	Dickerson, 2014 n = 1	Ishak, 2012 n = 1	Wang, 2005 n = 4	Venables, 2014 n = 12	Yang, 2014* n = 2	Ho, 2015* n = 7[2]	Irwin, 2006* n = 8	Johnson, 2016* n = 7	Montgomery, 2003* n = 3	Okajima, 2011* n = 10[2]	Seyffert, 2016* n =	Trauer, 2015* n = 17	van Straten, 2009* n = 7[2]	Zachariae, 2016* n= 11	Howell, 2014 n = 3	Taylor, 2014 n = 13	McCurry, 2007 n = 3	Times Cited
**Sleep Efficiency**																										
Altena, 2008	--	--	--	--	--	--	--	--	--	--	--	--	--	--	--	--	--	--	--	+	--	--	--	--	--	1
Arnedt, 2007	--	--	--	--	--	--	--	+	--	--	--	--	--	--	--	--	--	--	--	--	--	--	--	--	--	1
Arnedt, 2011	--	--	--	--	--	--	--	--	--	--	--	--	--	--	--	--	--	--	--	--	--	--	--	+	--	1
Berger, 2009	--	--	--	--	--	--	--	--	--	--	--	+	--	--	--	--	--	--	--	--	--	--	--	--	--	1
Blom, 2015a	--	--	--	--	--	+	--	--	--	--	--	--	--	--	--	--	--	--	--	--	--	--	--	--	--	1
Blom, 2015b	--	--	--	--	--	+	--	--	--	--	--	--	--	--	--	--	--	--	--	--	--	--	--	--	--	1
Bootzin and Stevens, 2005	--	--	--	--	--	--	--	+	--	--	--	--	--	--	--	--	--	--	--	--	--	--	--	--	--	1
Bothelius, 2013	--	--	--	--	--	--	--	--	--	--	--	--	--	--	--	--	--	--	--	+	--	--	--	--	--	1
Buysse, 2011	--	--	--	--	--	--	--	--	--	--	--	--	--	--	--	--	--	--	--	+	--	--	--	--	--	1
Chen, 2008	--	--	--	--	--	--	--	--	--	--	--	--	+	--	--	--	--	--	--	--	--	--	--	--	--	1
Chen, 2011	--	--	--	+	--	--	--	--	--	--	--	--	--	--	--	--	--	--	--	--	--	--	--	--	--	1
Currie, 2000	--	--	+	+	--	--	--	--	--	--	--	--	--	--	--	--	--	--	--	--	--	--	--	--	--	2

Currie, 2004	--	--	--	--	--	--	--	+	--	--	--	--	--	--	--	--	--	--	--	--	--	--	--	+	--	2
Davidson, 2001	--	--	--	--	--	--	--	--	+	--	--	--	--	--	--	--	--	--	--	--	--	--	--	--	--	1
Dirksen, 2008	--	--	--	--	--	--	--	--	--	--	--	+	--	--	--	--	--	--	--	--	--	--	--	--	--	1
Dixon, 2006	--	--	--	--	--	--	--	--	--	+	--	--	--	--	--	--	--	--	--	--	--	--	--	--	--	1
Edinger and Sampson, 2003	--	--	--	--	--	--	--	--	--	--	+	--	--	--	--	--	--	--	--	+	--	--	--	--	--	2
Edinger, 2001	--	--	--	--	--	--	--	--	--	--	+	--	--	--	+	--	--	--	--	+	--	--	--	--	--	3
Edinger, 2003	--	--	--	--	--	--	--	--	--	--	--	--	--	--	+	--	--	--	--		--	--	--	--	--	1
Edinger, 2007	--	--	--	--	--	--	--	--	--	--	--	--	--	--	--	--	--	--	--	+	--	--	--	--	--	1
Edinger, 2009	--	--	--	--	--	--	--	--	--	--	--	--	--	--	--	--	--	--	--	+	--	--	--	--	--	1
Epstein and Dirksen, 2007	--	--	+	+	--	--	--	--	--	--	--	+	--	--	--	+	--	--	--	--	--	--	--	--	--	4
Epstein, 2012	--	--	--	+	--	--	--	--	--	--	--	--	--	--	--	--	--	--	--	--	--	--	--	--	--	1
Espie, 2006	--	--	--	--	--	--	--	--	--	--	--	+	--	--	--	--	--	--	--	--	--	--	--	--	--	1
Espie, 2007	--	--	+	+	--	--	--	--	--	--	--	--	--	--	--	--	--	--	--	+	--	--	--	--	--	3
Espie, 2008a	--	--	--	--	--	--	--	--	--	--	--	+	--	--	--	+	--	--	--	--	--	--	+	--	--	3
Espie, 2008b	--	--	--	--	--	--	--	--	--	--	--	+	--	--	--	--	--	--	--	--	--	--	--	--	--	1
Espie, 2012	--	--	--	--	--	+	--	--	--	--	--	--	--	--	--	--	--	--	+	--	--	+	--	--	--	3
Fiorentino, 2008	--	--	--	--	--	--	--	--	--	--	--	+	--	--	--	--	--	--	--	--	--	--	--	--	--	1
Garland,	--	--	--	--	--	--	--	--	--	--	--	--	--	--	--	+	--	--	--	--	--	--	--	--	--	1

2014																										
Ho, 2014	--	--	--	--	--	+	--	--	--	--	--	--	--	--	--	--	--	--	+	--	--	+	--	--	--	3
Holmqvist, 2014	--	--	--	--	--	+	--	--	--	--	--	--	--	--	--	--	--	--	--	--	--	--	--	--	--	1
Jacobs, 2004	--	--	--	--	--		--	--	--	--	--	--	--	--	--	--	--	--	--	+	--	--	--	--	--	1
Kaldo, 2015	--	--	--	--	--	+	--	--	--	--	--	--	--	--	--	--	--	--	+	--	--	--	--	--	--	2
Lancee, 2012	--	--	--	--	--	+	--	--	--	--	--	--	--	--	--	--	--	--	+	--	--	+	--	--	--	3
Lancee, 2013a	--	--	--	--	--	+	--	--	--	--	--	--	--	--	--	--	--	--	--	--	--	--	--	--	--	1
Lancee, 2013b	--	--	--	--	--	+	--	--	--	--	--	--	--	--	--	--	--	--	--	--	--	--	--	--	--	1
Lancee, 2015	--	--	--	--	--	+	--	--	--	--	--	--	--	--	--	--	--	--	--	--	--	--	--	--	--	1
Lichstein, 1999	--	--	--	--	--	--	--	--	--	--	--	--	--	--	--	--	--	--	--	--	--	--	--	+	--	1
Lichstein, 2001	--	--	--	--	--	--	--	--	--	--	--	--	--	--	+	--	+	--	--	--	--	--	--	--	--	2
Lichstein, 2013	--	--	--	--	--	--	--	--	--	--	--	--	--	--	--	--	--	--	--	--	--	--	--	+	--	1
Lovato, 2014	--	--	--	--	--	--	--	--	--	--	--	--	--	--	--	--	--	--	--	+	--	--	--	--	--	1
Mack, 2013	--	+	--	--	--	--	--	--	--	--	--	--	--	--	--	--	--	--	--	--	--	--	--	--	--	1
Manber, 2008	--	--	--	--	--	--	--	--	--	--	--	--	--	--	--	--	--	--	--	--	--	--	--	+	--	1
Margolies, 2013	--	+	--	--	--	--	--	--	--	--	--	--	--	--	--	--	--	--	--	--	--	--	--	+	--	2
Matthews, 2010	--	--	--	--	--	--	--	--	--	--	--	+	--	--	--	--	--	--	--	--	--	--	--	--	--	1
Matthews, 2014	--	--	--	--	--	--	--	--	--	--	--	--	--	--	--	+	--	--	--	--	--	--	--	--	--	1
McCrae, 2007	--	--	--	--	--	--	--	--	--	--	--	--	--	--	--	--	--	--	--	+	--	--	--	--	--	1
Mimeault and Morin, 1999	--	--	--	--	--	--	--	--	--	--	+	--	--	--	--	--	--	--	--	--	--	--	--	--	--	1

Morin, 1993	--	--	+	--	--	--	--	--	--	--	--	--	--	--	+	--	+	--	--	+	--	--	--	--	+	4
Morin, 1999	--	--	--	+	--	--	--	--	--	--	--	--	--	--	+	--	+	--	--	+	--	--	--	--	--	4
Morin, 2004	--	--	--	--	--	--	--	--	--	--	--	--	--	--	--	--	--	--	--	--	--	--	--	+	--	1
Morin, 2012	--	--	--	--	--	--	--	--	--	--	+	--	--	--	--	--	--	--	--	--	--	--	--	--	--	1
Okajima, 2013	--	--	--	--	--	--	--	--	--	--	--	--	--	--	--	--	--	--	--	--	--	--	--	+	--	1
Ouellet and Morin, 2007	--	--	--	--	--	--	+	--	--	--	--	--	--	--	--	--	--	--	--	--	--	--	--	--	--	1
Pallesen, 2003	--	--	--	--	--	--	--	--	--	--	--	--	--	--	+	--	--	--	--	--	--	--	--	--	--	1
Quesnel, 2003	--	--	--	--	--	--	--	--	--	--	--	+	--	--	--	--	--	--	--	--	--	--	--	--	--	1
Quesnel, 2003	--	--	--	--	--	--	--	--	--	--	--	+	--	--	--	--	--	--	--	--	--	--	--	--	--	1
Rambod, 2013	--	--	--	--	--	--	--	--	--	--	--	--	+	--	--	--	--	--	--	--	--	--	--	--	--	1
Raymond, 2010	--	--	--	--	--	--	--	--	--	--	--	+	--	--	--	--	--	--	--	--	--	--	--	--	--	1
Riedel, 1995	--	--	--	--	--	--	--	--	--	--	--	--	--	--	+	--	--	--	--	--	--	--	--	--	--	1
Ritterband, 2009	+	--	--	--	--	+	--	--	--	--	--	--	--	--	--	--	--	--	+	--	--	+	--	--	--	4
Ritterband, 2012	+	--	--	--	--	+	--	--	--	--	--	--	--	--	--	+	--	--	--	--	--	+	+	--	--	5
Rybarczyk, 2002	--	--	+	--	--	--	--	--	--	--	--	--	--	--	+	--	--	--	--	--	--	--	--	--	+	3
Rybarczyk, 2005	--	--	--	+	--	--	--	--	--	--	--	--	--	--	--	--	--	--	--	--	--	--	--	--	+	1
Savard, 2005	--	--	--	+	--	--	--	--	--	--	--	+	--	--	--	+	--	--	--	--	--	--	+	--	--	4
Savard, 2014[1]	--	--	--	--	--	--	--	--	--	--	--	--	--	--	--	+	--	--	--	--	--	--	--	--	--	1
Sivertsen, 2006	--	--	--	--	--	--	--	--	--	--	--	--	--	--	--	--	--	--	--	+	--	--	--	--	--	1

Soeffing, 2008	--	--	--	--	--	--	--	--	--	--	--	--	--	--	--	--	--	--	--	+	--	--	--	+	--	2
Strom, 2004	+	--	--	--	--	+	--	--	--	--	--	--	--	--	--	--	--	--	+	--	--	+	--	--	--	4
Suzuki, 2008	--	--	--	--	--	--	--	--	--	--	--	--	--	--	--	--	--	--	--	--	--	+	--	--	--	1
Talbot, 2014	--	+	--	--	--	--	--	--	--	--	--	--	--	--	--	--	--	--	--	--	--	--	--	+	--	2
Taylor, 2012	--	--	--	--	--	--	--	--	--	--	--	--	--	--	--	--	--	--	--	--	--	--	--	+	--	1
Taylor, 2014	--	--	--	--	--	--	--	--	--	--	--	--	--	--	--	--	--	--	--	+	--	--	--	--	--	1
Thiart, 2015	--	--	--	--	--	--	--	--	--	--	--	--	--	--	--	--	--	--	+	--	--	+	--	--	--	2
Ulmer, 2011	--	+	--	--	--	--	--	--	--	--	--	--	--	--	--	--	--	--	--	--	--	--	--	+	--	2
Ustinov, 2013	--	+	--	--	--	--	--	--	--	--	--	--	--	--	--	--	--	--	--	--	--	--	--	--	--	1
Van Straten, 2013	--	--	--	--	--	--	--	--	--	--	--	--	--	--	--	--	--	--	--	--	--	+	--	--	--	1
Van Straten, 2014	--	--	--	--	--	+	--	--	--	--	--	--	--	--	--	--	--	--	+	--	--	--	--	--	--	2
Vincent, 2009	+	--	--	--	--	+	--	--	--	--	--	--	--	--	--	--	--	--	+	--	--	+	--	--	--	4
Vitiello, 2009	--	--	+	--	--	--	--	--	--	--	--	--	--	--	--	--	--	--	--	--	--	--	--	--	--	1
Wagley, 2013	--	--	--	--	--	--	--	--	--	--	--	--	--	--	--	--	--	--	--	--	--	--	--	+	--	1
Wu, 2006	--	--	--	--	--	--	--	--	--	--	--	--	--	--	--	--	--	--	--	+	--	--	--	--	--	1

Primary Studies n = 46	Brasure, 2015* n = 9	Cheng, 2012* n = 2	Ho, 2016* n = 5	Navarro-Bravo, 2015* n = 4	van Straten, 2007* n = 38[2]	Ye, 2016* n = 11	Bogdanov, 2017 n = 2	Dickerson, 2014 n = 2	Ishak, 2012 n = 1	Venables, 2014 n = 10	Johnson, 2016* n = 4	Seyffert, 2016* n = 4	Zachariae, 2016* n = 8	Howell, 2014 n = 4	Times Cited
**Insomnia Severity Index (ISI)**															
Arnedt, 2013	+	--	--	--	--	--	--	--	--	--	--	--	--	--	1
Bothelius, 2013	+	--	--	--	--	--	--	--	--	--	--	--	--	--	1
Byles, 2003	--	--	--	--	--	--	--	--	+	--	--	--	--	--	1
Casault, 2011	--	--	--	--	--	--	--	--		+	--	--	--	--	1
Dirksen and Epstein, 2008	--	--	--	--	--	--	--	+	--	+	--	--	--	--	2
Dirksen, 2008	--	--	--	--	--	--	--	--	--	+	--	--	--	+	2
Epstein and Dirksen, 2007	--	--	--	+	--	--	--	--	--	--	--	--	--	--	1
Epstein, 2012	--	--	--	+	--	--	--	--	--	--	--	--	--	--	1
Espie, 2012	--	--	--	--	--	+	--	--	--	--	--	--	+	--	2
Fiorentino, 2008	--	--	--	--	--	--	--	--	--	+	--	--	--	--	1
Fiorentino, 2009	--	--	--	--	--	--	--	--	--	--	--	--	--	+	1
Garland, 2014	--	--	--	--	--	--	--	--	--	--	+	--	--	--	2
Ho, 2014	--	--	--	--	--	+	--	--	--	--	--	+	+	--	3
Holmqvist, 2014	--	--	--	--	--	+	--	--	--	--	--	--	--	--	1
Jernelov, 2013	+	--	--	--	--	--	--	--	--	--	--	--	--	--	1
Jungquist, 2010	+	--	--	--	--	--	--	--	--	--	--	--	--	--	1
Kaldo, 2015	--	--	--	--	--	--	--	--	--	--	--	+	--	--	1
Lancee 2015	--	--	--	--	--	+	--	--	--	--	--	--	--	--	1
Lancee, 2012	--	--	--	--	--	+	--	--	--	--	--	--	+	--	2
Lancee, 2013a	--	--	--	--	--	+	--	--	--	--	--	--	--	--	1
Lancee, 2013b	--	--	--	--	--	+	--	--	--	--	--	--	--	--	1
Lu, 2016	--	--	--	--	--	--	+	--	--	--	--	--	--	--	1
Mack, 2013	--	--	+	--	--	--	--	--	--	--	--	--	--	--	1
Margolies, 2013	--	--	+	--	--	--	--	--	--	--	--	--	--	--	1
Matthews, 2014	--	--	--	--	--	--	--	--	--	--	+	--	--	--	2
Ouellet and	--	--	--	--	--	--	+	--	--	--	--	--	--	--	1

Morin, 2007															
Pigeon, 2012	+	--	--	--	--	--	--	--	--	--	--	--	--	--	1
Pillai, 2015	--	--	--	--	--	--	--	--	--	--	--	--	+	--	1
Quesnel, 2003	--	--	--	--	--	--	--	+	--	+	--	--	--	--	2
Raymond, 2010	--	--	--	--	--	--	--	--	--	+	--	--	--	--	1
Ritterband, 2009	+	+	--	--	--	+	--	--	--	--	--	--	+	--	4
Ritterband, 2011	--	--	--	--	--		--	--	--	--	--	--		+	1
Ritterband, 2012	--	--	--	--	--	+	--	--	--	+	--	--	+	--	3
Rybarczyk, 2005	--	--	--	+	--	--	--	--	--	--	--	--	--	--	1
Savard, 2005	--	--	--	+	--	--	--	--	--	+	+	--	--	+	4
Savard, 2011	--	--	--	--	--	--	--	--	--	+	--	--	--	--	1
Savard, 2013	--	--	--	--	--	--	--	--	--	+	--	--	--	--	1
Savard, 2014	--	--	--	--	--	--	--	--	--	--	+	--	--	--	2
Smith, 2015	+	--	--	--	--	--	--	--	--	--	--	--	--	--	1
Strom, 2004	--	--	--	--	--	+	--	--	--	--	--	--	--	--	1
Talbot, 2014	--	--	+	--	--	--	--	--	--	--	--	--	--	--	1
Tang, 2012	+	--	--	--	--	--	--	--	--	--	--	--	--	--	1
Thiart, 2015	--	--	--	--	--	--	--	--	--	--	--	+	+	--	2
Ulmer, 2011	--	--	+	--	--	--	--	--	--	--	--	--	--	--	1
Ustinov, 2013	--	--	+	--	--	--	--	--	--	--	--	--	--	--	1
Vincent, 2009	+	+	--	--	--	+	--	--	--	--	--	+	+	--	5

Primary Studies n = 26	**Ballesio, 2017* n = 20[3]**	**Dickerson, 2014 n = 1**	**Yang, 2014* n = 2**	**Tang, 2015* n = 6**	**Howell, 2014 n = 2**	**Times Cited**
			**Fatigue Severity**			
Arendt, 2011	+	--	--	--	--	--
Barsevick, 2010	--	--	--	+	--	1
Berger, 2009	--	--	--	+	--	1
Chen, 2008	+	--	+	--	--	1
Chen, 2011	+	--	+	--	--	1
Davidson, 2001	--	+	--	--	--	1
Dirksen, 2008	+	--	--	--	--	--

Espie, 2008	+	--	--	+	+	2
Ho, 2014	+	--	--	--	--	--
Irwin, 2014	+	--	--	--	--	--
Jernelov, 2012	+	--	--	--	--	--
Kapella, 2011	+	--	--	--	--	--
Lichstein, 2001	+	--	--	--	--	--
Lovato, 2014	+	--	--	--	--	--
Martinez, 2013	+	--	--	--	--	--
Martinez, 2014	--	--	--	+	--	1
Matthews, 2014	+	--	--	--	--	--
Morgan, 2012	+	--	--	--	--	--
Pigeon, 2012	+	--	--	--	--	--
Rios, 2013	+	--	--	+	--	1
Ritterband, 2011	--	--	--	--	+	1
Ritterband, 2012	+	--	--	+	--	1
Savard, 2005	+	--	--	--	--	--
Savard, 2014	+	--	--	--	--	--
Tang, 2012	+	--	--	--	--	--
Vincent, 2009	+	--	--	--	--	--

Primary Studies n = 7	**Brooks, 2014 n = 1**	**Dickerson, 2014 n = 2**	**Ishak, 2012 n = 4**	**Howell, 2014 n = 1**	**Times Cited**
		**Health-related Quality of Life**			
Arnedt, 2007	+	--	--	--	1
Byles, 2003	--	--	+	--	1
Dirksen and	--	+	--	+	2

Epstein, 2008					
Dixon, 2006	--	--	+	--	1
Espie, 2008	--	--	+	--	1
Quesnel, 2003	--	+	--	--	1
Van Houdenhove, 2011	--	--	+	--	1

CBT compared to active controls			
Primary Studies n = 2	**van Straten, 2009*<br>n = 3[2]**	**Seyffert, 2016*<br>n = 2**	**Times Cited**
**Total Sleep Time**			
Holmqvist, 2014	--	+	1
Blom, 2015	--	+	1
Primary Studies n = 4	**van Straten, 2009*<br>n = 3[2]**	**Seyffert, 2016*<br>n = 2**	**Times Cited**
**Sleep Efficiency**			
Holmqvist, 2014	--	+	1
Blom, 2015	--	+	1
Edinger, 2001	--	--	--
Morin, 1999	--	--	--

[1]This study involves two trials: in-person CBT-I vs. control and video-based CBT-I vs. control
[2] Unable to determine some or all of the primary studies associated with this outcome
[3] The authors of the Ballesio, 2017 review did not clearly report which studies were included in the analyses. The listed studies are all of the primary studies related to fatigue, but are not included in the times cited count.
*systematic review with meta-analysis

Table F9. Primary studies across systematic reviews that compared behavioural interventions to inactive controls

Primary Studies	**Brasure, 2015*<br>n = 5**	**Buscemi, 2005*<br>n = 13**	**Hwang, 2016*<br>n = 5**	**Bogdanov, 2017<br>n = 1**	**Brooks, 2014<br>n = 1**	**Hellström, 2011<br>n = 1**	**McCurry, 2007<br>n = 1**	**Miller, 2014<br>n = 4**	**Tamrat, 2013<br>n = 3**	**Wang, 2005<br>n = 1**	**Times Cited**	**Outcome(s)**
Alessi, 2005	--	--	+	--	--	--	--	--	--	--	1	SQ
Bliwise, 1995	--	--	--	--	--	--	--	+	--	--	1	SOL, TST

Primary Studies	**Brasure, 2015* n = 5**	**Buscemi, 2005* n = 13**	**Hwang, 2016* n = 5**	**Bogdanov, 2017 n = 1**	**Brooks, 2014 n = 1**	**Hellström, 2011 n = 1**	**McCurry, 2007 n = 1**	**Miller, 2014 n = 4**	**Tamrat, 2013 n = 3**	**Wang, 2005 n = 1**	**Times Cited**	**Outcome(s)**
Buysse, 2011	+	--	+	--	--	--	--	--	--	--	2	SOL, WASO, SQ
Carr-Kaffashan, 1979	--	+	--	--	--	--	--	--	--	--	1	SOL
Choliz, 1995	--	+	--	--	--	--	--	--	--	--	1	SOL
Edinger, 2001	+	--	--	--	--	--	--	--	--	--	1	TST, WASO
Epstein, 2012	+	--	--	--	--	--	--	+	--	--	2	SOL, TST, WASO, SQ, SE
Espie, 1989	--	+	--	--	--	--	--	--	--	--	1	SOL, TST
Friedman, 2000	--	--	--	--	--	--	+	+	--	--	2	SOL, TST, WASO, SE
Germain, 2006	--	--	+	--	--	--	--	--	--	--	1	SQ
Greef and Conradie, 1998	--	--	--	--	+	--	--	--	--	--	1	SQ
Haynes, 1974	--	+	--	--	--	--	--	--	--	--	1	SOL
Haynes, 1977	--	+	--	--	--	--	--	--	--	--	1	SOL
Haynes, 1977	--	+	--	--	--	--	--	--	--	--	1	SOL
Hong and Kim, 2009	--		+	--	--	--	--	--	--	--	1	SQ
Hughes, 1978	--	+	--	--	--	--	--	--	--	--	1	SOL
Jacobs, 1993	--		--	--	--	--	--	--	--	+	1	SOL
Lacks, 1983	--	+	--	--	--	--	--	--	--	--	1	SOL
Lareau, 2008	--		--	--	--	--	--	--	+	--	1	SQ
Lichstein, 2001	+	+	--	--	--	--	--	--	--	--	2	SOL, TST, WASO, SE
McCrae, 2007	+	--	--	--	--	--	--	--	--	--	1	SOL, WASO
McCurry, 2013	--	--	+	--	--	--	--	--	--	--	1	SQ
McDowell, 1998	--	--	--	--	--	--	--	--	+	--	1	SQ
Mitchell, 1979	--	+	--	--	--	--	--	--	--	--	1	SOL
Nicassio, 1974	--	+	--	--	--	--	--	--	--	--	1	SOL
Richardson, 2003	--	--	--	--	--	+	--	--	--	--	1	SQ
Sanavio, 1990	--	+	--	--	--	--	--	--	--	--	1	SOL, WASO

Primary Studies	**Brasure, 2015* n = 5**	**Buscemi, 2005* n = 13**	**Hwang, 2016* n = 5**	**Bogdanov, 2017 n = 1**	**Brooks, 2014 n = 1**	**Hellström, 2011 n = 1**	**McCurry, 2007 n = 1**	**Miller, 2014 n = 4**	**Tamrat, 2013 n = 3**	**Wang, 2005 n = 1**	**Times Cited**	**Outcome(s)**
Shealy, 1979	--	+	--	--	--	--	--	--	--	--	1	SOL
Soeffing, 2008	+	--	--	--	--	--	--	--	--	--	1	SOL, WASO
Stanton, 1989	--	+	--	--	--	--	--	--	--	--	1	SOL
Taylor, 2010	--	--	--	--	--	--	--	+	--	--	1	SOL, TST, WASO, SE
Toth, 2007	--	--	--	--	--	--	--	--	+	--	1	SQ
Vuletic, 2016	--	--	--	+	--	--	--	--	--	--	1	SQ

*systematic reviews with meta-analysis

Table F10. Primary studies across included systematic reviews that examined mindfulness based interventions

Primary Studies	**Gong, 2016* n = 2**	**Venables, 2014 n = 3**	Times Cited	Outcome(s)
Black, 2015	+	--	1	SQ
Britton, 2012	+	--	1	SOL, TST, SQ, SE
Carlson, 2005	--	+	1	SQ, SE
Lengacher, 2013	--	+	1	SE
Ong, 2014	+	--	1	TST, SE
Shapiro, 2003	--	+	1	SE
Zhang, 2015	+	--	1	SOL, SQ

*systematic review with meta-analysis

Table F11. Primary studies across included systematic reviews that compared combination therapy to inactive controls

Primary Studies (n=4)	**Buscemi, 2005* n = 2**	**Chiesa, 2009 n = 2**	**Times Cited**	**Outcome(s)**

Milby, 1993	+	--	1	TST
Morin, 1999	+	--	1	TST
Heidenreich, 2006	--	+	1	SOL, TST
Ong, 2008	--	+	1	TST, SQ

*systematic review with meta-analysis
